# Supplementary material for: Multimodal impact of copper-silicon hybrid nanotools towards bacterial leaf streak, wheat biochemistry and productivity parameters
Source: PLoS One. 2025 Jul 15;20(7):e0327021. doi: 10.1371/journal.pone.0327021 (PMC12262875; doi:10.1371/journal.pone.0327021)
Supplement: Attachment — Submitted filename: PONE-D-24-41835_R1.pdf [file pone.0327021.s005.pdf]

Manuscript
Click here to access/download;Manuscript;Waqas Ahmad NPs Paper (PLoS One).docx

FORTITUDE OF PARTHENIUM HYSTEROPHORUS MEDIATED COPPER-SILICON HYBRID NANO TOOLS TOWORDS BACTERIAL LEAF STREAK AND BIOCHEMICAL AND AGRONOMIC ATTRIBUTES OF WHEAT

WAQAS AHMAD[1], MUHAMMAD ATIQ[1]*, NASIR AHMED RAJPUT[1], RIZWANA MAQBOOL[2], MUHMMAD JAMIL SHAFI[3], ABDUL JABBAR[4], SOHAIL ASAD[5], MUHAMMAD JAHANZAIB MATLOOB[1], HASSAN ZIA[1], MUHAMMAD USAMA[1]

[1]Department of Plant Pathology, Faculty of Agriculture, University of Agriculture, Faisalabad, Pakistan.
[2]Department of Plant Breeding and Genetics, Faculty of Agriculture, University of Agriculture, Faisalabad, Pakistan.
[3]Department of Plant Pathology, Constituent College Depalpur, Okara, University of Agriculture, Faisalabad, Pakistan.
[4]Department of Agronomy, Faculty of Agriculture, University of Agriculture, Faisalabad, Pakistan.
[5]School of Biology and Chemistry, Pu'er University, Pu'er, China.

* Corresponding author

E-mail: muhammad.atiq@uaf.edu.pk (MA)

Abstract

Wheat production is substantially harmed by biotic and abiotic stresses. Among biotic stresses, bacterial leaf streak (BLS) of wheat which is caused by bacterium *Xanthomonas translucens* pv. *undulosa* (*Xtu*) induces crop yield losses up to 10-40% depending upon inoculum level and susceptibility of host. This multi-step investigation encompassed the evaluation of the antibacterial potency of parthenium-mediated CuNPs and SiNPs. Green synthesized Cu-Si nanoparticles were evaluated under lab and greenhouse conditions employing a Complete Randomized Design (CRD) and under field conditions adopting Randomized Complete Block Design (RCBD) alone and in combination. The scanning electron microscopy and X-ray diffraction showed that CuNPs and SiNPs exhibited semi-spherical and spherical morphology with average particle size of 61.49nm and 14.36nm, respectively. Among the tested nanoparticles, maximal inhibition one was expressed by CuNPs + SiNPs (19.06 mm), followed by CuNPs (14.14 mm) and SiNPs (10.56 mm) as compared to control. The least disease incidence under greenhouse (14.75%) and field-scale evaluation (29.46%) was expressed by CuNPs + SiNPs, followed by CuNPs and SiNPs in comparison with control. Moreover, the execution of most effective dosage of CuNPs + SiNPs enhanced the amounts of enzymatic and non-enzymatic antioxidants like SOD, POD, CAT, H_2O_2, TPC, TSS by 3.09, 3.01, 1.87, 7.35, 3.61 and 1.08 µg/g, respectively in comparison with control. Similarly, most effective dosage of CuNPs + SiNPs increased the yield-related attributes of the wheat plant such as root length (4.52 cm), shoot length (4.063 cm), chlorophyll contents (5.56 SPAD), spike length (15.98 cm), spikelets per spike (19.88) and No. of grains per spike (31.97), 100 grains weight (17.07 g), plant height (81.46 cm), stomatal conductance (288.69 m.mol $m^{-2}s^{-1}$), and root biomass (492.15 kg/ha). Recent findings emphasize the potential of ecological nanotechnology-based tactics in plant disease management. Furthermore, nanoparticles applications are emphasized for the effective management of disease through enhanced enzymatic and non-enzymatic antioxidant defense system and yield-related attributes of wheat. It is presumed that the application of CuNPs and SiNPs was an eco-friendly tactic for managing the bacterial leaf streak of wheat and enhancing the antioxidant defense system and yield-related attributes of wheat.

Introduction

Pakistan ranks eighth in production, yielding 31.4 million tonnes over an area of 9.4 million hectares. Wheat contributes 9.0 % to agricultural value-added and 2.2 % to GDP of Pakistan [16]. It is crucial for diets, furnishing key nutrients such as proteins, carbohydrates, vitamins, fats, minerals and nutrients [10, 29, 64]. It aids in treating heart diseases, constipation, cancer, obesity, and diabetes [10]. Wheat by-products (wheat bran, white flour, bread flour, cake flour, gluten flour, semolina, farina) are used in animal feed, bioethanol, baking, cosmetics, and pharmaceuticals [31].

Different biotic (fungi, bacteria, nematode, virus) and abiotic (drought, temperature, salinity, floods, radiations, pollutants) factors are responsible for the reduced production impacting qunatity and quality of crop [26]. Among the biotic factors, bacterial leaf streak (BLS) which is caused by the gram-negative *Xanthomonas translucens* pv. *undulosa* is a major global threat to wheat production [49, 51]. Optimal growth was noted at 28-30°C, but it terminates at 36°C, particularly when moisture levels are high [34]. The disease can cause up to 10-40% yield losses worldwide, dependent on inoculum levels and host susceptibility [20]. The pathogen also endures on crop debris, soil, and weeds, penetrating the plants through stomata, wounds and hydathodes. Inside the plant, the pathogen multiply in the mesophyll tissues producing exopolysaccharides which lead to biofilm formation [39]. *Xtu* uses type III effectors (T3E) via a type III secretion system (T3SS) to initiate the disease symptoms [62] which appeared as water-soaked lesions that gradually turn yellow, brown, and necrotic. These lesions eventually ooze a honey-like exudate, which spreads under humid conditions and forms a thin, transparent layer [45].

Different management approaches are in practice to cope the bacterial leaf streak of wheat. The most efficient approach for managing the disease is the emloyment of resistant varieties. However, under favorable conditions, even these varieties become susceptible [9].

Excessive use of chemicals for disease management can disrupt plant functions and cause toxicity, leading to a range of health issues and organ impairments [22]. Considering the hazardous effects of chemicals and the limitations of other control measures, advanced strategies such as green nanotechnology are essential for effective disease management [32]. This emerging technique uses nanoparticles to treat plant bacterial disease being preferred over chemical and physical methods due to its cost-effectiveness and environmental friendliness [18, 61]. Nanoparticles (1-100 nm) are used in small doses to effectively inhibit microbial enzymes, damage essential molecules, cause cell wall damage, and stimulate oxidative stress which led to microbial death [23, 36]. Use of weeds for the integration of green-based nanoparticles offer an eco-friendly approach to manage plant diseases [7]. Researchers have developed green-based nanoparticles, using weed extracts to control bacterial plant diseases [30]. Copper nanoparticles (Cu-NPs) demonstrated strong antibacterial activity against *Xanthomonas campestris* [33], and *Xanthomonas campestris* pv. *vesicatoria* [69]. Copper and silver nanoparticles synthesized from *Eucalyptus globulus* leaves were effective against *Xanthomonas citri* [9], and Cu-NPs also inhibited *Ralstonia solanacearum* [41]. Similarly, silicon nanoparticles (Si-NPs) proved effective against *Pectobacterium carotovorum* and *X. campestris* pv. *carotae* in carrots [65, 76].

Nanoparticles (NPs) improve the wheat biochemistry by enhancing the nutrient acquisition, photosynthetic efficacy, and inducing stress resistance [6]. NPs regulate the enzymatic activity of antioxidants activity such as SOD, POD, and CAT that scavenge the ROS and combat the oxidative damage [25]. NPs also regulate the non-enzymatic antioxidants acitvity like H_2O_2, TPC, and TSS that make sure the strengthening of defense responses and reducing oxidative stress, and in result, ensuring the better overall plant health and resilience [27]. NPs not only suppress the harmful effects of pathogens but also promote the plant growth metrics [38]. So, the current research effort was directed to investigate the antibacterial function

of *Parthenium hysterophorus* mediated Cu-Si hybrid nanotools towards bacterial leaf streak of wheat and its effect on the physiology and biochemistry of wheat plant.

Materials and methods

Synthesis of weed-based nanoparticles (NPs)

Parthenium (*Parthenium hysterophorus* L.) was collected from the Agronomy Department Research Area, and was shade-dried for about 4-5 days. After this, it was sun-dried over 3 days. Then, it was kept in a dry oven (101-1AB) to remove remaining moisture at 65 ℃ for 4 hours. After oven drying, pestle and mortar was employed to grind the weed into fine powder. Then, powder was filtered by using muslin cloth to obtain a fine powder without impurities. 20 g of this fine powder was added in a beaker (250 ml) containing 100 ml methanol, covered with an aluminium foil and kept under darkness over a period of 24 hours (for effective extraction, deterrence of photodegradation and facilitates the uniform interaction). After this, the solution was stirred in a magnetic stirrer at 70 ℃ for about 15 minutes (for uniform dissolution and helped in boosting the reaction). Then, filtration of the solution was done through Whatman's Filter Paper No. 41. For the preparation of Silicon and Copper nanoparticles, 17g sodium metasilicate (Na_2SiO_3) and 17g copper sulphate ($CuSO_4$) was added in the filtrate separately and put it on magnetic stirrer (C-MAG HS 4) for mixing of solution and then placed it in ultrasonic cleaner (VEVOR) at 60°C for breaking the bond for 1 hour. To obtain Si and Cu, nanoparticles in powder form, the solution was placed in a furnace oven (Carbolite PF60, higher temp. processing, uniform heating under controlled conditions) [9]. For the preparation of hybrid nanoparticles, both silicon and copper nanoparticles were mixed.

Characterization of parthenium mediated nanoparticles

The characterization of the parthenium mediated nanoparticles was performed by adopting different techniques as performed by [44].

UV–visible spectroscopy

The biological reduction of the solution was pursued by determining its absorbance with a UV-visible spectrophotometer (V-750) over the range of 200-800 nm. 1 ml of the sample was used and 1ml of distilled water was used as control.

X-ray diffraction spectroscopy

The average size of nanoparticles was determined by employing diffractometer (Philips X-Pert MMP). The P Rofit software was employed to control the diffractometer on a computer, with molybdenum radiation (MoK) used as the source (λ = 0.7093 A). The Mo-tube operated at 50 kV and 40 mA to facilitate the bombardment process.

Scanning electron microscopy

The morphological characteristics of the nanoparticles synthesized through parthenium extract was assessed by employing scanning electron microscopy (SEM). For SEM analysis, A 1 mg sample was deposited onto carbon-coated grids and imaged at different magnifications for the assessment of its morphological characters.

In-vitro assessment of green synthesized NPs against *Xanthomonas translucens* pv. *undulosa*

The inhibition zone technique was used for the assessment of green synthesized nanoparticles (copper and silicon) from weed (*Parthenium hysterophorus* L.) against *Xanthomonas translucens* pv. *undulosa* under laboratory conditions. For this purpose, one litre bacterial growth media (NA) was prepared in the Laboratory. To get three concentrations

(0.025, 0.05, and 0.075 %) of NPs, 0.025, 0.05, and 0.075 g of (Cu and Si) powder alone and in combination (Cu + Si) were added in bottle (250 ml) containing 100 ml distilled water separately. After that, NA medium was poured into 9 cm Petri plates and allowed to solidify. Through autoclaved cotton swab, bacterial culture was streaked on Petri plates, and this whole procedure was performed within a laminar flow cabinet (RTVL1312, UK) to prevent the contamination. Circular pieces of the filter paper (1 cm) were cut and sterilized in an autoclave (Robus Technologies, RTA85) at pressure of 15 PSI and 121 °C for 15 minutes. Then, these pieces of filter paper were dipped into formulated concentrations (0.025, 0.05, and 0.075 %) of NPs (Cu and Si) alone and in combination. These dipped filter paper pieces were placed in the centre of the Petri plates having a culture of *Xanthomonas translucens* pv. *undulosa*. In case of control treatment, filter paper pieces were dipped in distilled water rather than in nanoparticle formulations. The plates were wrapped with paraffin and incubated (Heraeus) at 26 ± 2 °C for optimal growth of bacterium.

The inhibition zone was measured by employing digital vernier calliper (500-196, Mitutoyo) after 24, 48, and 72 hours [9]. This trial was designed under Complete Randomized Design (CRD) adopting three replications of each treatment.

In-vivo **evaluation of green synthesized NPs against bacterial leaf streak of wheat**

A field experiment was conducted following the methodology of [9] with trivial adjustments at the Department Research Area. PBG Line 3 was sown employing Randomized Complete Block Design (RCBD) by upholding the P-P and R-R spacing at 10-25 cm and 10-30 cm, respectively for better growth and development. Four treatments (CuNPs + SiNPs, CuNPs, SiNPs, and Control) with three replications of each treatment were evaluated against bacterial leaf streak of wheat. Aqueous suspension of the bacterium *Xtu* was prepared from

culture grown for 48 hours and employing spectrophotometer (Hitachi U-2001, 121003), bacterial concentration @ $1x10^8$ CFU/ml was determined.

The bacterium *Xtu* was artificially introduced in wheat plants by using spray method at the stage of 4-5 leaves. This was done early in the morning when maximum number of stomata were opened in the daytime. After the typical symptom's expression, most effective concentration (0.75 %) of nanoparticles was sprayed in the field while, in the case of control, only distilled water was sprayed on plants. Data regarding the incidence of the bacterial leaf streak was recorded after 7, 14, and 21 days using the formula given below,

$$\text{Disease incidence (\%)} = \frac{No.\ of\ Diseased\ Plants}{Total\ No.\ of\ Plants\ Observed}\ x\ 100 \quad [60]$$

Alterations in the biochemical profiling of wheat leaves after application of nanoparticles

Both treated and untreated wheat plants were collected from the field, and their leaves were cut into small pieces. A 0.5 g leaf sample was ground by employing a sterilized pestle and mortar with potassium phosphate buffer (pH 5). Then, the mixture was centrifuged (TGL-16A) for 5 minutes at 12000 revolutions per minute (rpm). The resulting supernatant was used for advanced biochemical analysis.

Determination of SOD (Superoxide dismutase) from treated and ntreated wheat leaves

For this purpose, a reaction mixture containing 200 µL methionine, 100 µL enzyme extract, 200 µL triton X, 500 µL potassium phosphate buffer (pH 5), and 100 µL NBT (as an indicator for superoxide radicals $O_2^{\bullet-}$) with 800 µL distilled water was prepared. It was kept under UV light for 15 minutes. Absorbance was recorded after the addition of 100 µL riboflavin employing spectrophotometer (BioTek, 800TS) at 560 nm wavelength [15].

Determination of POD (Peroxidase) from treated and untreated wheat leaves

For the determination of POD from treated and untreated wheat leaves, a reaction mixture consisting of 20 mM guaiacol, 40 mM H_2O_2 (100 µL), 100 µL enzyme extract, and 800 µL potassium phosphate buffer (pH 5) was prepared. Absorbance was recorded employing spectrophotometer (BioTek, 800TS) at frequency of 470 nm.

Determination of CAT (Catalase) from treated and untreated wheat leaves

A reaction mixture (100 µL enzyme extract and 100 µL of 5.9 mM H_2O_2) was prepared to determine the concentration of catalase in wheat leaves. A spectrophotometer (BioTek, 800TS) was employed to record the absorbance at 240 nm.

Determination of H_2O_2 (Hydrogen peroxide) from treated and untreated wheat leaves

Leaves of treated and untreated plants were collected and sliced into small-sized pieces. A 50 mg leaf sample was taken along with trichloro acetic acid buffer (enhance extraction efficiency, stabilize and prevent degradation of H_2O_2), grinded, and then centrifuged (TGL-16A) at 4°C for 15 minutes at 12000 rpm (stability of sample and preservation of enzymatic content). Then the solution was filtered and treated with potassium phosphate buffer (pH 7) followed by potassium iodide buffer. The final mixture was placed in a digital incubator (RTI-250) for 5 minutes providing a controlled environment for optimal reaction conditions and by using microplate reader (BioTek, 800TS), absorbance was recorded at frequency of 390 nm.

Determination of TPC (Total phenolic contents) from treated and untreated wheat leaves

100 µL enzyme extract was prepared to determine the TPC from treated and untreated wheat leaves. A reaction mixture was prepared consisting of 800 µL Na_2CO_3 (700 mM) and 200 µL of 10% Folin-Ciocalteu (F-C) reagent and allowed to react for 1 hour. Using microplate

reader (Biotek, 800TS), reading was recorded at frequency of 765 nm.

Determination of TSS (Total soluble sugars) from treated and untreated wheat leaves

The 100 mg sample was subjected to hydrolysis through 5 mL of 2.5 N hydrochloric acid for 3 hours in water bath (HT, XMTD-204), then neutralized with sodium carbonate (Na_2CO_3) until bubbling ceased, and finally cooled at at room temperature. A solution of 100 mL was prepared and processed for centrifugation (TGL-16A). 0.5 µL resultant supernatant along with aliquots @ 1 mL was obtained and used for more studies. Special values were developed by using 0.20, 0.4, 0.6, 0.8, and 1 mL as operational values and 0 represented as a blank. To make the volume more than one mL, distilled water was used, and anthrone reagent @ 4 mL was added. For heating the solution, a water bath (HT, XMTD-204) which allows the consistent and controlled heating during hydrolysis without evaporation was used, and absorbance was measured employing spectrophotometer (BioTek, 800TS) at 630 nm [75].

Assessment of impact of NPs on the agronomic attributes of wheat

Root length, shoot length, spike length and overall plant height were measured by manual method using measuring tape (HV-1455) in cm. SPAD (SPAD-502 Plus) meter was used for the precise determination of chlorophyll contents in the leaves of wheat plant by clamping it gently on the leaf. By detaching the spike from the stem, spikelets were counted manually. Total no. of grains per spike were counted precisely by manual method after the threshing process. For the determination of 100 grains weight, 100 grains were counted and then weighed on a weighing balance (SF-400A) in grams. Porometer (AP4 Porometer) was used to determine the stomatal conductance of the wheat leaves by gently attaching the porometer sensor's head to the leaf. It was recorded in mmol m^2s^{-1}. While, for the determination of the root biomass, root samples were collected washed with tap water to remove soil particles.

After this, these are allowed to air dry and then kept in dry oven (101-1AB) for 65 ℃ for 48-72 hours until all water content is removed, and only dry biomass is left behind. Then these were weighed on a digital weighing balance (NL, 7017 X) and recorded in kg ha^{-1}.

Statistical Analysis

The effects of copper and silicon nanoparticles on the inhibition zone of *Xtu*, BLS disease incidence, alterations in biochemical profile, and agronomic attributes of wheat plants were tested using analysis of variance (ANOVA) with LSD test at $p \leq 0.05$ employing Statistix 8.1 [67]. The graphical representation of the data was performed on Origin 2024b software.

Results

Characterization of nanoparticles

The sub-microscopic images of copper and silicon nanoparticles by scanning electron microscopy (SEM) showed that CuNPs exhibited semi-spherical morphology and SiNPs expressed spherical shape. While X-ray diffraction (XRD) analysis of CuNPs and SiNPs showed that NPs expressed crystalline nature exhibiting average particle size of 61.49 nm and 14.36 nm, respectively as shown in Table 1.

Table 1. Characterization of copper and silicon nanoparticles

Nanoparticles	Shape	Average size
CuNPs	Semi-spherical	61.49 nm
SiNPs	Spherical	14.36 nm

Inhibition zone and Disease incidence of bacterial leaf streak of wheat

Maximum inhibition zone was expressed by combination of CuNPs + SiNPs (19.06 mm), followed by CuNPs (14.14 mm) and SiNPs (10.56 mm) as compared to control. In case of interaction between T×C and T×D, CuNPs + SiNPs was the most effective followed by CuNPs and SiNPs in comparison with control (Fig 1 and 2A). Under greenhouse conditions, minimum disease incidence was expressed by CuNPs + SiNPs (14.75 %), followed by CuNPs (34.92 %) and SiNPs (41.11 %) as compared to control. Interactions between T×C and T×D expressed that CuNPs + SiNPs exhibited minimum disease followed by CuNPs and SiNPs as compared to control (Fig 2B). While under field scale evaluation of nanoparticles, results revealed that CuNPs + SiNPs expressed minimum disease incidence followed by CuNPs and SiNPs as compared to control. T×D interaction with most effective concentration revealed that CuNPs + SiNPs expressed minimum disease incidence, followed by CuNPs and SiNPs (Fig 3).

Fig 1. Impact of copper and silicon nanoparticles on the Inhibition zone (mm) of *Xtu* in relation to different concentrations.

Fig 2. Impact of copper and silicon nanoparticles on Inhibition zone (mm) (A), Disease incidence (%) under greenhouse (B) and Disease incidence (%) under field conditions. Small letters represent the statistical significance using LSD test at $p \leq 0.05$.

Fig 3. Impact of copper and silicon nanoparticles on Disease incidence (%) under field conditions. Small letters represent the statistical significance using LSD test at $p \leq 0.05$.

Estimation of alterations in the biochemical profiling of wheat

All enzymatic and non-enzymatic antioxidants activity was enhanced substantially after the application of copper and silicon nanoparticles alone and in hybrid. Application of most effective concentration of CuNPs + SiNPs enhanced the activity of SOD (3.09), POD (3.01),

CAT (1.87), H_2O_2 (7.35), TPC (3.61), TSS (1.08) µg/g followed by CuNPs (2.56, 2.30, 1.58, 6.53, 3.03, 0.94) µg/g and SiNPs (2.09, 2.1.18, 1.28, 5.60, 2.10, 0.781) µg/g, respectively as compared to control (Fig 4).

Fig 4. Determination of SOD, POD, CAT, H_2O_2, TPC and TSS (µg/g) from treated and untreated wheat leaves after the application of nanoparticles. Small letters represent the statistical significance using LSD test at $p \leq 0.05$.

Assessment of copper and silicon nanoparticles on the agronomic attributes of wheat

All the agronomic attributes of wheat were augmented substantially after the application of copper and silicon nanoparticles alone and in hybrid. Application of most effective concentration of CuNPs + SiNPs expressed the maximum root length (4.52 cm), shoot length (4.063 cm), chlorophyll contents (5.56 SPAD), spike length (15.98 cm), spikelets per spike (19.88) and No. of grains per spike (31.97), followed by CuNPs and SiNPs in comparison with control as shown in Fig 5A. Similarly, CuNPs + SiNPs expressed the maximum 100 grains weight (17.07 g), plant height (81.46 cm), stomatal conductance (288.69 m.mol $m^{-2}s^{-1}$), and root biomass (492.15 kg/ha), followed by CuNPs and SiNPs in comparison with control as shown in Fig 5B. Impact of nanoparticles on the yield-related aspects of plant are shown in Fig 6.

Fig 5A. Impact of copper and silicon nanoparticles on root length (cm), shoot length (cm), chlorophyll contents (SPAD), spike length (cm), spikelets per spike and No. of grains per spike. Small letters correspond to the statistical significance using LSD test at $p \leq 0.05$.

Fig 5B. Impact of copper and silicon nanoparticles on 100 grains weight (g), plant height (cm), stomatal conductance (m.mol $m^{-2}s^{-1}$) and root biomass (kg/ha). Small letters correspond to the statistical significance using LSD test at $p \leq 0.05$.

Fig 6. Impact of nanoparticles on the yield-related attributes of wheat in relation to different concentrations 0.25 (b), 0.50 (c), 0.75% (d) of nanoparticles as compared to control (a).

Discussion

Excessive use of chemicals for the disease management can disrupt plant functions and causes toxicity, leading to a range of health issues and organ impairments [22]. So, considering the hazardous effects of chemicals and the limitations of other control measures, advanced strategies such as green nanotechnology is essential for the effective disease management [32]. The current research efforts were focused on the antibacterial efficacy of *Parthenium hysterophorus* mediated copper and silicon nanoparticles and their impacts on the biochemical profiling and yield-related attributes of the wheat for the effective management of BLS of wheat. The results revealed that hybrid (Cu + Si) nanoparticles expressed maximal inhibition zone under laboratory conditions, while minimum disease incidence in greenhouse and field conditions, followed by CuNPs and SiNPs as compared to control. Findings of the current research are encouraged by the outcomes of [21] who evaluated nanoparticles (Cu, MgO, ZnO) NPs, and concluded that CuNPs have the highest antibacterial potency against *Xanthomonas translucens* pv. *undulosa*. Results of this research are endorsed by the results of [6, 8, 11, 13] who observed the antimicrobial activity of CuNPs. The nanoparticles were characterized by employing UV-visible spectroscopy, scanning electron microscopy, and X-ray diffraction spectroscopy. The results revealed that copper nanoparticles exhibited the light brown to dark green color, semi-spherical shape with average particle size of 61.49 nm. These findings are in line with the work of [28, 44, 47], who performed the characterization of CuNPs synthesized from *Parthenium hysterophorus*. Similarly, silicon nanoparticles exhibited the spherical shape with the average particle size of 14.36 nm and these outcomes are in agreement with the findings of [2, 42], who reported the average size and shape of the SiNPs.

Copper and silicon nanoparticles have unique mechanisms for their uptake by the plants and suppressing the bacterial pathogens. In case of copper nanoparticles (CuNPs), they are accumulated on the bacterial cell membrane and release Cu^{2+} ions that interact with carboxyl as well as sulfhydryl groups in the peptidoglycan layer [70]. CuNPs enter the host plant through endocytosis [73]. Upon entry into the host plant, these Cu^{2+} ions lead to the generation of reactive oxygen species (ROS), lipid peroxidation resulting in the interference with cell biomolecules, the protein functioning [52] and disrupt the ATP production, results in the disruption of the DNA, which leads to death of the bacterium [63].

Plants get SiNPs (5-20 mm) through endocytosis from roots and invaded into the xylem tissues, translocated to different organs of the plant and stored in the shoots [46]. After accumulation, nanoparticles form a second layer of cuticle-Si, which strengthen the physical barriers against pathogenesis [17]. These changes in plants make it more difficult for the pathogens to invade and cause disease. Concurrently, SiNPs directly inhibit the pathogen growth by rupturing cell membranes which led to the spillage of cell contents resulting in cell death [76].

When plants are infected with *Xanthomonas* spp., then, a prominent alteration in the antioxidant defense enzymes like SOD, POD, CAT, and non-enzymatic antioxidants such as TPC, TSS, H_2O_2 was noted [66]. A surge in the induction of reactive oxygen species was experienced after the infection, which leads to the oxidative burst in the wheat plants [59]. Imbalance in the oxidants and antioxidants also resulted in the oxidative stress which potentially damage the plant cells. Higher levels of ROS resulted in the localized cell death and induce substantial tissue damage [12]. These combined consequences, lead to the reduction in plant health and vigor which resulted the limited growth and yield of plant. So, a most pressing need is to regulate the antioxidant defense system of wheat, so that they can exhibited the best growth and development [19]. In this study, the progressive alterations in the enzymatic

antioxidants (SOD, POD, CAT) and non-enzymatic antioxidants (TPC, TSS, H_2O_2) from the treated and untreated wheat leaves after the application of copper and silicon nanoparticles alone and in hybrid was determined. The results revealed that Cu-Si hybrid nanoparticles expressed the maximum amounts of SOD, POD, CAT, TSS, TPC, H_2O_2 followed by copper nanoparticles and silicon nanoparticles in comparison with control. The application of the nanoparticles augmented the level of SOD, POD and CAT through controlled ROS production, directly interacting with enzymes, improving cellular environment, and upregulation of enzymatic genes expression which contribute, to a more vigorous and effective antioxidant response, aiding the plant to survive under oxidative stress and make better its resilience to pathogens [42]. Superoxide dismutase (SOD), the first defense line against ROS like superoxide radicals (O_2^{-}•) was activated in response to application of nanoparticles that converts the O_2^{-}• into hydrogen peroxide and oxygen ($2O_2^{-\bullet}+2H^+ \rightarrow H_2O_2+O_2$) [54, 77]. Hydrogen peroxide (H_2O_2) is an important non-enzymatic antioxidant which acts as a signaling molecule as well as ROS. It is lethal to the pathogen at the optimum level but, when it exceeds, induces the oxidative stress to the plants. Considering this oxidative stress, catalase activity (CAT) was triggered which converts the hydrogen peroxide from the dismutation ROS into water and oxygen ($2H_2O_2 \rightarrow 2H_2O+O_2$). It is capable to detoxify millions of H_2O_2 molecules in seconds [50]. After detoxification of ROS, peroxidases (POD) reinforced the cell walls of the wheat plants acting as a physical barrier against pathogen's penetration into host plant. These results are in line with the study of [68], who reported the increase of antioxidant defense system of wheat plants after the application of nanoparticles. Phenolic compounds such as TPC are rich in hydroxyl groups that provide hydrogen atoms to counteract ROS like superoxide radicals and hydrogen peroxide efficiently and diminished oxidative stress in plants through reinforcing the cell walls, protecting lipid membranes, induction of SAR [40, 43, 72]. Total soluble sugars (TSS) like glucose, sucrose, and fructose are essential sources of energy that is

produced by metabolic processes such as citric acid cycle and glycolysis and is essential for the upregulation of enzymatic antioxidant defense system. These enzymes need ATP under stress where its demand is heightened for its effective activity [3]. The outcomes of the existent study aligned with the work of [1, 71], who demonstrated that nanoparticles upregulated the activity of both enzymatic and non-enzymatic antioxidants of the plants. Additionally, [24, 37, 53, 57-58] concluded that SiNPs exhibited rise in antioxidant defense system and noted significant enhancement in the growth of wheat plant by stimulating the antioxidant defense system through the application of silicon nanoparticles.

Nanoparticles allow precise release of bioactive compounds that not only suppress the negative effects of the phytopathogens but also promote the plant growth by inducing resistance in infected plants [38]. Current research effort was also directed to investigate the impact of *Parthenium hysterophorus* mediated Cu-Si hybrid nanotools towards the growth traits of wheat plant. The results of the study revealed that hybrid (CuNPs + SiNPs) nanoparticles expressed the maximum root length (cm), shoot length (cm), plant height (cm), spike length (cm), No. of grains per spike, spikelets per spike, 100 grains weight (g), chlorophyll contents (SPAD), root biomass (kg/ha) and stomatal conductance (m.mol $m^{-2}s^{-1}$), followed by CuNPs and SiNPs in comparison with control. Nanoparticles improved the chlorophyll contents of wheat by improving the uptake of the essential nutrients and then translocated to the leaves where synthesis of chlorophyll happened. Outcomes of this study are supported by the results of [14], who demonstrated that nanoparticles substantially enhanced the root length, shoot length and chlorophyll contents. Stomatal conductance was enhanced through the application of nanoparticles. Nanoparticles increased the water holding capacity of the wheat by strengthening the cell wall and increased root growth resulting in an increase in turgor pressure within guard cells (control stomatal opening) resulting in the greater stomatal conductance. These findings are corroborated by the outcomes of [4], who revealed that stomatal

conductance is elevated in the wheat plants treated with nanoparticles. The nanoparticles at lower concentrations significantly enhanced the overall plant height by improving the source-sink relationship, efficient nutrient and water uptake resulting in the elevated photosynthetic activity. Similar results were observed by the findings of [5, 56], who demonstrated that nanoparticles posed positive impacts on the plant height of wheat and rice. A surge in the shoot length, root length, no. of grains per spike was observed after the application of nanoparticles to wheat plant. This was due to the efficient nutrient uptake and stimulation of plant growth hormones such as gibberellins, cytokinin, and auxins responsible for the plant growth and these outcomes are endorsed by the results of [74, 48]. At optimum level, nanoparticles improved the agronomic parameters of the wheat like number of grains per spike, root biomass, grains weight, spike length. These results are in line with the work of [55], who expressed that application of nanoparticles enhanced the agronomic parameters of wheat. Moreover, existent results are aligned with outcomes of [35, 58, 68], who reported that nanoparticles significantly enhanced the agronomic attributes of wheat.

Conclusion

Under laboratory conditions, combination of CuNPs and SiNPs showed maximal inhibition zone against *Xanthomonas translucens* pv. *undulosa*. While, under field and greenhouse conditions, hybrid nanoparticles formulation demonstrated the lowest disease incidence compared to the individual applications of copper and silicon nanoparticles. Similarly, enzymatic and non-enzymatic defense system and yield-related attributes of wheat plant. Therefore, it is concluded that the hybrid form of copper and silicon nanoparticles is highly recommended to manage bacterial leaf streak of wheat and enhance antioxidant defense system and yield-related attributes of wheat plant effectively. Future research should focus on

optimizing the application methods and dosages for different environmental conditions to maximize efficacy.

Acknowledgment

We authors extend our gratitude to the Molecular Bacteriology and Plant Pathogen Interaction Laboratory, Department of Plant Pathology UAF, Pakistan for providing the laboratory facilities necessary for conducting research.

References

1. Abdelkader M, Geioushy RA, Fouad OA, Khaled AG, Liudmila PV. Investigation the activities of photosynthetic pigments, antioxidant enzymes and inducing genotoxicity of cucumber seedling exposed to copper oxides nanoparticles stress. Sci Hortic. 2022 Nov 17;305: 111364. doi: https://doi.org/10.1016/j.scienta.2022.111364
2. Abdelrhim AS, Mazrou YS, Nehela Y, Atallah OO, El-Ashmony RM, Dawood MF. Silicon dioxide nanoparticles induce innate immune responses and activate antioxidant machinery in wheat against *Rhizoctonia solani*. Plants. 2021 Dec 14;10(12): 2758. doi: https://doi.org/10.3390/plants10122758
3. Afzal S, Chaudhary N, Singh NK. Role of soluble sugars in metabolism and sensing under abiotic stress. In: Aftab T, Hakeem KR, editors. Plant growth regulators. Springer, Singapore; 2021. pp. 305-334. doi: https://doi.org/10.1007/978-3-030-61153-8
4. Ahmed F, Javed B, Razzaq A, Mashwani ZU. Applications of copper and silver nanoparticles on wheat plants to induce drought tolerance and increase yield. IET Nanobiotech. 2021 Feb;15(1): 68-78. doi: https://doi.org/10.1049/nbt2.12002
5. Ahmed T, Noman M, Manzoor N, Shahid M, Abdullah M, Ali L, et al. Nanoparticle-based amelioration of drought stress and cadmium toxicity in rice via triggering the stress responsive genetic mechanisms and nutrient acquisition. Ecotoxicol Environ Saf. 2021 Feb

1;209: 111829.doi: https://doi.org/10.1016/j.ecoenv.2020.111829

6. Ali S, Ulhassan Z, Shahbaz H, Kaleem Z, Yousaf MA, Ali S, et al. Magnesium oxide nanoparticles as novel sustainable approach in enhancing crop tolerance to abiotic and biotic stresses. Environ Sci: Nano. 2024 Jul 5;11(8): 3250-3267. doi: https://doi.org/10.1039/D4EN00417E

7. Ameta SK, Ameta SC. Eco-friendly approaches of using weeds for sustainable plant growth and production. In: Husen A, editor. Plant performance under environmental stress. Springer, Singapore; 2021. pp. 559-592. doi: https://doi.org/10.1007/978-3-030-78521-5

8. Atiq M, Fatima T, Rajput NA, Usman M, Kachelo GA, Ahmad U, et al. *Moringa olefra* plant based copper and silver nano particles and its antibacterial activity towards leaf spot of chilli caused by *Xanthomonas campestris* pv. *vesicatoria*. Int J Phytopath. 2023 Apr 30;12(1): 63-72. doi: https://doi.org/10.33687/phytopath.012.01.4306

9. Atiq M, Mazhar HM, Rajput NA, Ahmad U, Hameed A, Lodhi A, et al. Green synthesis of silver and copper nanoparticles from leaves of *Eucalyptus globulus* and assessment of its antibacterial potential towards *Xanthomonas citri* pv. *citri* causing citrus canker. Appl Ecol Environ Res. 2022 Jan 1;20(3): 2205-2213. doi: http://dx.doi.org/10.15666/aeer/2003_22052213

10. Awulachew MT. The role of wheat in human nutrition and its medicinal value. Glob Acad J Med Sci. 2020;2(6): 50-54. doi: https://doi.org/10.36348/gajms.2020.v02i06.001

11. Chauhan H, Patel M, Patel P, Tiwari S, Jinal HN, Amaresan N. Assessment of copper (Cu) nanoparticle for their biocontrol activity against *Xanthomonas oryzae* pv. *oryzae*, growth promotion, and physiology of rice (*Oryza sativa* L.) plants. Lett Appl Microbiol. 2023 Jan;76(1): ovac066. doi: https://doi.org/10.1093/lambio/ovac066

12. Choudhary A, Kumar A, Kaur N. ROS and oxidative burst: Roots in plant development. Plant Divers. 2020 Feb 1;42(1): 33-43. doi: https://doi.org/10.1016/j.pld.2019.10.002

13. Chowdhury AR, Kumar R, Mahanty A, Mukherjee K, Kumar S, Tribhuvan KU, et al. Inhibitory role of copper and silver nanocomposite on important bacterial and fungal pathogens in rice (*Oryza sativa*). Sci Rep. 2024 Jan 20;14(1):1779. doi: https://doi.org/10.1038/s41598-023-49918-0

14. Essa HL, Abdelfattah MS, Marzouk AS, Shedeed Z, Guirguis HA, El-Sayed MM. Biogenic copper nanoparticles from *Avicennia marina* leaves: Impact on seed germination, detoxification enzymes, chlorophyll content and uptake by wheat seedlings. PLoS One. 2021 Apr 15;16(4): e0249764. doi: https://doi.org/10.1371/journal.pone.0249764

15. Giannopolitis CN, Ries SK. Superoxide dismutases: I. Occurrence in higher plants. Plant Physiol. 1977 Feb 1;59(2): 309-314. doi: https://doi.org/10.1104/pp.59.2.309

16. GOP. Economic advisor's wing, Finance Division, Government of Pakistan. Pakistan Economic Survey 2023-2024. 2024;2: 24. Available from: https://finance.gov.pk/survey_2024.html

17. Goswami P, Mathur J, Srivastava N. Silica nanoparticles as novel sustainable approach for plant growth and crop protection. Heliyon. 2022 Jul 7;8(7): e09908. doi: https://doi.org/10.1016/j.heliyon.2022.e09908

18. Haidri I, Shahid M, Hussain S, Shahzad T, Mahmood F, Hassan MU, et al. Efficacy of biogenic zinc oxide nanoparticles in treating wastewater for sustainable wheat cultivation. Plants. 2023 Aug 25;12(17): 3058. doi: https://doi.org/10.3390/plants12173058

19. Hameed A, Atiq M, Sahi ST, Rajput NA, Ahmed Z, Alam MW, et al. Biochemical base of resistance in citrus against canker disease. Pak J Agri Sci. 2021 Jan 16;58(6): 1850-1858. doi: 10.21162/PAKJAS/21.11

20. Hangamaisho A, Bleakley BH, Ali S, Yabwalo D, Byamukama E. Aggressiveness of *Xanthomonas translucens* pv. *undulosa* isolates and differential reaction among spring wheat varieties under a controlled environment. Plant Health Prog. 2024 Feb 21;25(1): 33-

36. doi: https://doi.org/10.1094/PHP-03-23-0020-RS

21. Hangamaisho A. Efficacy of nanomaterials and biorationals on the bacterial leaf streak management in wheat. M.Sc. Thesis, South Dakota State University; 2022. Available from: https://openprairie.sdstate.edu/etd2/416

22. Hashimi MH, Hashimi R, Ryan Q. Toxic effects of pesticides on humans, plants, animals, pollinators and beneficial organisms. Asian Plant Res J. 2020 Aug 5;5(4): 37-47. doi: https://doi.org/10.9734/aprj/2020/v5i430114

23. Hernández-Díaz JA, Garza-García JJ, Zamudio-Ojeda A, León-Morales JM, López-Velázquez JC, García-Morales S. Plant-mediated synthesis of nanoparticles and their antimicrobial activity against phytopathogens. J Sci Food Agri. 2021 Mar 15;101(4): 1270-1287. doi: https://doi.org/10.1002/jsfa.10767

24. Huang G, Zuverza-Mena N, White JC, Hu H, Xing B, Dhankher OP. Simultaneous exposure of wheat (*Triticum aestivum* L.) to CuO and S nanoparticles alleviates toxicity by reducing Cu accumulation and modulating antioxidant response. Sci Total Environ. 2022 Sep 15;839: 156285. doi: https://doi.org/10.1016/j.scitotenv.2022.156285

25. Huang Q, Ayyaz A, Farooq MA, Zhang K, Chen W, Hannan F, et al. Silicon dioxide nanoparticles enhance plant growth, photosynthetic performance, and antioxidants defence machinery through suppressing chromium uptake in *Brassica napus* L. Environ Poll. 2024 Feb 1;342: 123013. doi: https://doi.org/10.1016/j.envpol.2023.123013

26. Hussain D, Asrar M, Khalid B, Hafeez F, Saleem M, Akhter M, et al. Insect pests of economic importance attacking wheat crop (*Triticum aestivum* L.) in Punjab, Pakistan. Int J Trop Insect Sci. 2022 Feb;42(1): 9-20. doi: https://doi.org/10.1007/s42690-021-00574-9

27. Ijaz M, Khan F, Ahmed T, Noman M, Zulfiqar F, Rizwan M, et al. Nanobiotechnology to advance stress resilience in plants: Current opportunities and challenges. Mat Today Bio. 2023 Aug 6;22: 100759. doi: https://doi.org/10.1016/j.mtbio.2023.100759

28. Iliger KS, Sofi TA, Bhat NA, Ahanger FA, Sekhar JC, Elhendi AZ, et al. Copper nanoparticles: Green synthesis and managing fruit rot disease of chilli caused by *Colletotrichum capsici*. Saudi J Biol Sci. 2021 Feb 1;28(2): 1477-86. doi: https://doi.org/10.1016/j.sjbs.2020.12.003

29. Iqbal MJ, Shams N, Fatima K. Nutritional quality of wheat. In: Ansari M, editor. Wheat-recent advances. IntechOpen, London, UK; 2022 Aug 23. pp. 1-19. Available from: https://www.intechopen.com/chapters/81564

30. Javaid A, Munir R, Khan IH, Shoaib A. Control of the chickpea blight, *Ascochyta rabiei*, with the weed plant, *Withania somnifera*. Egypt J Biol Pest Cont. 2020 Dec;30: 114. doi: https://doi.org/10.1186/s41938-020-00315-z

31. Kanojia V, Kushwaha NL, Reshi M, Rouf A, Muzaffar H. Products and byproducts of wheat milling process. Int J Chem Stud. 2018;6(4): 990-993.

32. Karamchandani BM, Dalvi SG, Bagayatkar M, Banat IM, Satpute SK. Prospective applications of chitosan and chitosan-based nanoparticles formulations in sustainable agricultural practices. Biocat Agri Biotech. 2024 Jun;58: 103210. doi: https://doi.org/10.1016/j.bcab.2024.103210

33. Kashyap P, Shirkot P, Das R, Pandey H, Singh D. Biosynthesis and characterization of copper nanoparticles from *Stenotrophomonas maltophilia* and its effect on plant pathogens and pesticide degradation. J Agri Food Res. 2023 Sep 1;13: 100654. doi: https://doi.org/10.1016/j.jafr.2023.100654

34. Kaur N. Genotypic characterization and fungicide resistance monitoring for Virginia populations of *Parastagonospora nodorum* in wheat. Doctoral dissertation, Virginia Polytechnic Institute and State University. 2021. Available from: http://hdl.handle.net/10919/112959

35. Kausar H, Mehmood A, Khan RT, Ahmad KS, Hussain S, Nawaz F, et al. Green synthesis

and characterization of copper nanoparticles for investigating their effect on germination and growth of wheat. PLoS One. 2022 Jun 21;17(6): e0269987. doi: https://doi.org/10.1371/journal.pone.0269987

36. Khan I, Saeed K, Khan I. Nanoparticles: Properties, applications and toxicities. Arab J Chem. 2019 Nov 1;12(7): 908-931. doi: https://doi.org/10.1016/j.arabjc.2017.05.011

37. Khan ZS, Rizwan M, Hafeez M, Ali S, Adrees M, Qayyum MF, et al. Effects of silicon nanoparticles on growth and physiology of wheat in cadmium contaminated soil under different soil moisture levels. Environ Sci Poll Res. 2020 Feb;27: 4958-4968. doi: https://doi.org/10.1007/s11356-019-06673-y

38. Kralova K, Jampilek J. Applications of nanomaterials in plant disease management and protection. In: Ingle AP, editor. Nanotechnology in agriculture and agroecosystems. Micro Nano Tech. 2023 Jan 1: 239-296. doi: https://doi.org/10.1016/B978-0-323-99446-0.00013-1

39. Ledman KE, Curland RD, Ishimaru CA, Dill-Macky R. *Xanthomonas translucens* pv. *undulosa* identified on common weedy grasses in naturally infected wheat fields in Minnesota. Phytopathol. 2021 Jul 30;111(7): 1114-1121. doi: https://doi.org/10.1094/PHYTO-08-20-0337-R

40. Liu W, Cui X, Zhong Y, Ma R, Liu B, Xia Y. Phenolic metabolites as therapeutic in inflammation and neoplasms: Molecular pathways explaining their efficacy. Pharm Res. 2023 Jul 1;193: 106812. doi: https://doi.org/10.1016/j.phrs.2023.106812

41. Luong HT, Nguyen CX, Lam TT, Nguyen TH, Dang QL, Lee JH, et al. Antibacterial effect of copper nanoparticles produced in a Shewanella-supported non-external circuit bioelectrical system on bacterial plant pathogens. RSC Adv. 2022;12(7): 4428-4436. doi: https://doi.org/10.1039/D1RA08187J

42. Manzoor N, Ali L, Ahmed T, Rizwan M, Ali S, Shahid MS, et al. Silicon oxide

nanoparticles alleviate chromium toxicity in wheat (*Triticum aestivum* L.). Environ Poll. 2022 Dec 15;315: 120391. doi: https://doi.org/10.1016/j.envpol.2022.120391

43. Martínez-Rubio R, Centeno ML, García-Angulo P, Álvarez JM, Acebes JL, Encina A. The role of cell wall phenolics during the early remodelling of cellulose-deficient maize cells. Phytochem. 2020 Feb 1;170: 112219. doi: https://doi.org/10.1016/j.phytochem.2019.112219

44. Mary RS, Mahesh MK, Pruthvi ML. Biosynthesis of copper nanoparticles using *Parthenium hysterophorus* leaf extract and screening its antimicrobial activity. Asian J Pharm Clinic Res. 2020;13(1): 93-99. doi: http://dx.doi.org/10.22159/ajpcr.2020.v13i1.36076

45. Mehmood A, Fahad A, Amin A, Ullah S, Fayyaz M, Fateh FS. A comprehensive review of significant bacterial diseases affecting wheat. Pak J Phytopathol. 2023 Jun 26;35(1): 191-199. doi: https://doi.org/10.33866/phytopathol.035.01.0868

46. Mittal D, Kaur G, Singh P, Yadav K, Ali SA. Nanoparticle-based sustainable agriculture and food science: Recent advances and future outlook. Front Nanotech. 2020 Dec 4;2: 579954. doi: https://doi.org/10.3389/fnano.2020.579954

47. Nazir A, Rehman SU, Abbas M, Iqbal DN, Ahsraf AR, Iqbal M. Green synthesis and characterization of copper nanoparticles using *Parthenium hysterophorus* extract: Antibacterial and antioxidant activities evaluation. Chem Int. 2022;8(2): 68-76. doi: https://doi.org/10.5281/zenodo.6849915

48. Nazir MA, Hasan M, Mustafa G, Tariq T, Ahmed MM, Golzari Dehno R, et al. Zinc oxide nano-fertilizer differentially effect on morphological and physiological identity of redox-enzymes and biochemical attributes in wheat (*Triticum aestivum* L.). Sci Rep. 2024 Jun 7;14(1): 13091. doi: https://doi.org/10.1038/s41598-024-63987-9

49. Niri MD, Tarighi S, Taheri P. Defense activation in wheat against *Xanthomonas translucens*

via application of biological and chemical inducers. J Plant Pathol. 2023 May;105(2): 493-505. doi: https://doi.org/10.1007/s42161-023-01324-1

50. Orlikowski L, Sas-Paszt L, Wojdyła A, Orlikowska T. The use of hydrogen peroxide and silver nanoparticles in horticulture. J Horti Res. 2023 Dec 29;31(2): 1-22. doi: https://doi.org/10.2478/johr-2023-0037

51. Osdaghi E, Taghavi SM, Aliabadi AA, Khojasteh M, Abachi H, Moallem M, et al. Detection and diagnosis of bacterial leaf streak on small grain cereals: From laboratory to field. Phytopathol. 2023 Nov 27;113(11): 2024-2036. doi: https://doi.org/10.1094/PHYTO-09-22-0343-SA

52. Raffi M, Mehrwan S, Bhatti TM, Akhter JI, Hameed A, Yawar W, et al. Investigations into the antibacterial behavior of copper nanoparticles against *Escherichia coli*. Ann Microbiol. 2010 Mar;60: 75-80. doi: https://doi.org/10.1007/s13213-010-0015-6

53. Rahman SU, Xuebin Q, Yasin G, Cheng H, Mehmood F, Zain M, et al. Role of silicon on root morphological characters of wheat (*Triticum aestivum* L.) plants grown under Cd-contaminated nutrient solution. Acta Physiol Plan. 2021 Mar 16; 43:60. doi: https://doi.org/10.1007/s11738-021-03228-y

54. Rasheed Z. Superoxide dismutase: Challenges, opportunities, and promises for clinical translation. Int J Health Sci. 2024 May;18(3): 1-3. doi: https://pubmed.ncbi.nlm.nih.gov/38721138

55. Rashid MI, Shah GA, Sadiq M, Amin NU, Ali AM, Ondrasek G, et al. Nanobiochar and copper oxide nanoparticles mixture synergistically increases soil nutrient availability and improves wheat production. Plants. 2023 Mar 14;12(6): 1312. doi: https://doi.org/10.3390/plants12061312

56. Raza MA, Aslam MU, Valipour M, Iqbal R, Haider I, Mustafa AE, et al. Seed priming with selenium improves growth and yield of quinoa plants suffering drought. Sci Rep. 2024 Jan

9;14(1): 886. doi: https://doi.org/10.1038/s41598-024-51371-6

57. Raza MA, Zulfiqar B, Iqbal R, Muzamil MN, Aslam MU, Muhammad F, et al. Morpho-physiological and biochemical response of wheat to various treatments of silicon nano-particles under drought stress conditions. Sci Rep. 2023 Feb 15;13(1): 2700. doi: https://doi.org/10.1038/s41598-023-29784-6

58. Riaz M, Zhao S, Kamran M, Rehman NU, Mora-Poblete F, Maldonado C, et al. Effect of nano-silicon on the regulation of ascorbate-glutathione contents, antioxidant defense system and growth of copper stressed wheat (*Triticum aestivum* L.) seedlings. Front Plant Sci. 2022 Oct 13;13: 986991. doi: https://doi.org/10.3389/fpls.2022.986991

59. Rizvi A, Chandrawal R, Khan MH, Ahmed B, Umar S, Khan MS. Microbiological control of *Xanthomonas* induced bacterial leaf streak disease of wheat via phytocompounds and ros processing enzymes produced under biotic stress. J Plant Growth Reg. 2024 Feb;43(2): 601-623. doi: https://doi.org/10.1007/s00344-023-11119-4

60. Seem RC. Disease incidence and severity relationships. Ann Rev Phytopathol. 1984 Sep;22(1): 133-150.

61. Shafqat U, Maqsood A, Ishfaq A, Mustafa S, Rasheed Y, Mahmood F, et al. Green nanotechnology for plant bacterial diseases management in cereal crops: a review on metal-based nanoparticles. Notulae Bot Horti Agrobotan Cluj-Napoca. 2023 Sep 27;51(3): 13333. doi: https://doi.org/10.15835/nbha51313333

62. Shah SM, Haq F, Ma W, Xu X, Wang S, Xu Z, et al. $Tal_{1NXtc01}$ in *Xanthomonas translucens* pv. *cerealis* contributes to virulence in bacterial leaf streak of wheat. Front Microbiol. 2019 Sep 4;10: 2040. doi: https://doi.org/10.3389/fmicb.2019.02040

63. Shende S, Ingle AP, Gade A, Rai M. Green synthesis of copper nanoparticles by *Citrus medica* Linn. (Idilimbu) juice and its antimicrobial activity. World J Microbiol Biotechnol. 2015 Jun;31: 865-873. Doi: https://doi.org/10.1007/s11274-015-1840-3

64. Shewry PR, Hey SJ. The contribution of wheat to human diet and health. Food Ener Sec. 2015 Oct;4(3):178-202. doi: https://doi.org/10.1002/fes3.64

65. Siddiqui ZA, Hashmi A, Khan MR, Parveen A. Management of bacteria *Pectobacterium carotovorum*, *Xanthomonas campestris* pv. *carotae*, and fungi *Rhizoctonia solani*, *Fusarium solani* and *Alternaria dauci* with silicon dioxide nanoparticles on carrot. Int J Veg Sci. 2020 Nov 1;26(6): 547-557. doi: https://doi.org/10.1080/19315260.2019.1675843

66. Silveira PR, Nascimento KJ, Andrade CC, Bispo WM, Oliveira JR, Rodrigues FA. Physiological changes in tomato leaves arising from *Xanthomonas gardneri* infection. Physiol Mol Plant Pathol. 2015 Oct 1;92: 130-138. doi: https://doi.org/10.1016/j.pmpp.2015.10.001

67. Steel, R.G.D., J.H. Torrie and D.A. Dickey. Principles and Procedures of Statistics: A biometrical approach, 3rd Ed. McGraw-Hill., United States of America. 1997.

68. Thakur N, Rana S, Pathak A. Impact of chemically synthesized copper nanoparticles on growth and biochemical profiling of wheat (*Triticum aestivum* L.). Int J Biol, Phar Alli Sci. 2022 Jun 1;11: 2638-2651. doi: https://doi.org/10.31032/IJBPAS/2022/11.6.6127

69. Varympopi A, Dimopoulou A, Papafotis D, Avramidis P, Sarris I, Karamanidou T, et al. Antibacterial activity of copper nanoparticles against *Xanthomonas campestris* pv. *vesicatoria* in tomato plants. Int J Mol Sci. 2022 Apr 7;23(8): 4080. doi: https://doi.org/10.3390/ijms23084080

70. Vasiliev G, Kubo AL, Vija H, Kahru A, Bondar D, Karpichev Y, et al. Synergistic antibacterial effect of copper and silver nanoparticles and their mechanism of action. Sci Rep. 2023 Jun 6;13(1): 9202. doi: https://doi.org/10.1038/s41598-023-36460-2

71. Wang W, Ren Y, He J, Zhang L, Wang X, Cui Z. Impact of copper oxide nanoparticles on the germination, seedling growth, and physiological responses in *Brassica pekinensis* L. Environ Sci Poll Res. 2020 Sep;27: 31505-31515. doi: https://doi.org/10.1007/s11356-020-

09338-3

72. War AF, Nanda SA, Bashir I, Rehmaan S, Sheergojri IA, Rehman IU, et al. Plant phenolics role in bacterial disease stress management in plants. In: Lone R, Khan S, Al-Sadi AM, editors. Plant Phenolics in Biotic Stress Management. Springer, Singapore. 2024 Feb 28. pp. 217-241. doi: https://doi.org/10.1007/978-981-99-3334-1

73. Wasule DL, Shingote PR, Saxena S. Exploitation of functionalized green nanomaterials for plant disease management. Disco Nano. 2024 Jul 18;19(1): 118. doi: https://doi.org/10.1186/s11671-024-04063-z

74. Yasmeen F, Raja NI, Razzaq A, Komatsu S. Proteomic and physiological analyses of wheat seeds exposed to copper and iron nanoparticles. Biochim Biophys Acta (BBA)-Prot Proteo. 2017 Jan 1;1865(1): 28-42. doi: https://doi.org/10.1016/j.bbapap.2016.10.001

75. Yemm EW, Willis A. The estimation of carbohydrates in plant extracts by anthrone. Biochem J. 1954 Jul;57(3): 508-514. doi: https://doi.org/10.1042%2Fbj0570508

76. Yuvaraj M, Priya RS, Jagathjothi N, Saranya M, Suganthi N, Sharmila R, et al. Silicon nanoparticles (SiNPs): Challenges and perspectives for sustainable agriculture. Physiol Mol Plant Pathol. 2023 Oct 5: 102161. doi: https://doi.org/10.1016/j.pmpp.2023.102161

77. Zhao H, Zhang R, Yan X, Fan K. Superoxide dismutase nanozymes: an emerging star for anti-oxidation. J Mat Chem B. 2021;9(35): 6939-57. doi: https://doi.org/10.1039/D1TB00720C

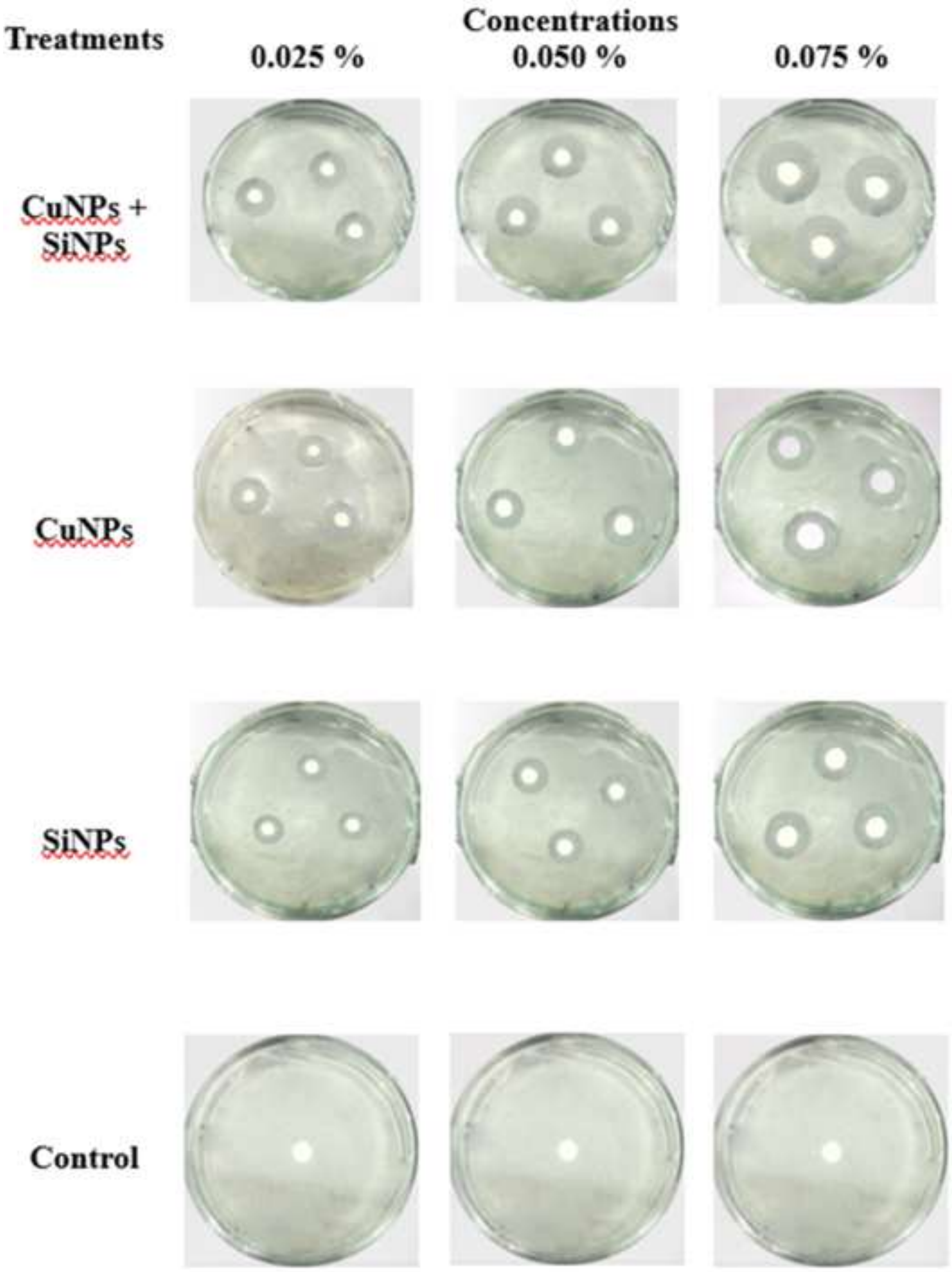
Treatments
Concentrations
0.025 %
0.050 %
0.075 %
CuNPs + SiNPs
CuNPs
SiNPs
Control

Figure2

Click here to access/download;Figure;Fig 2.tif

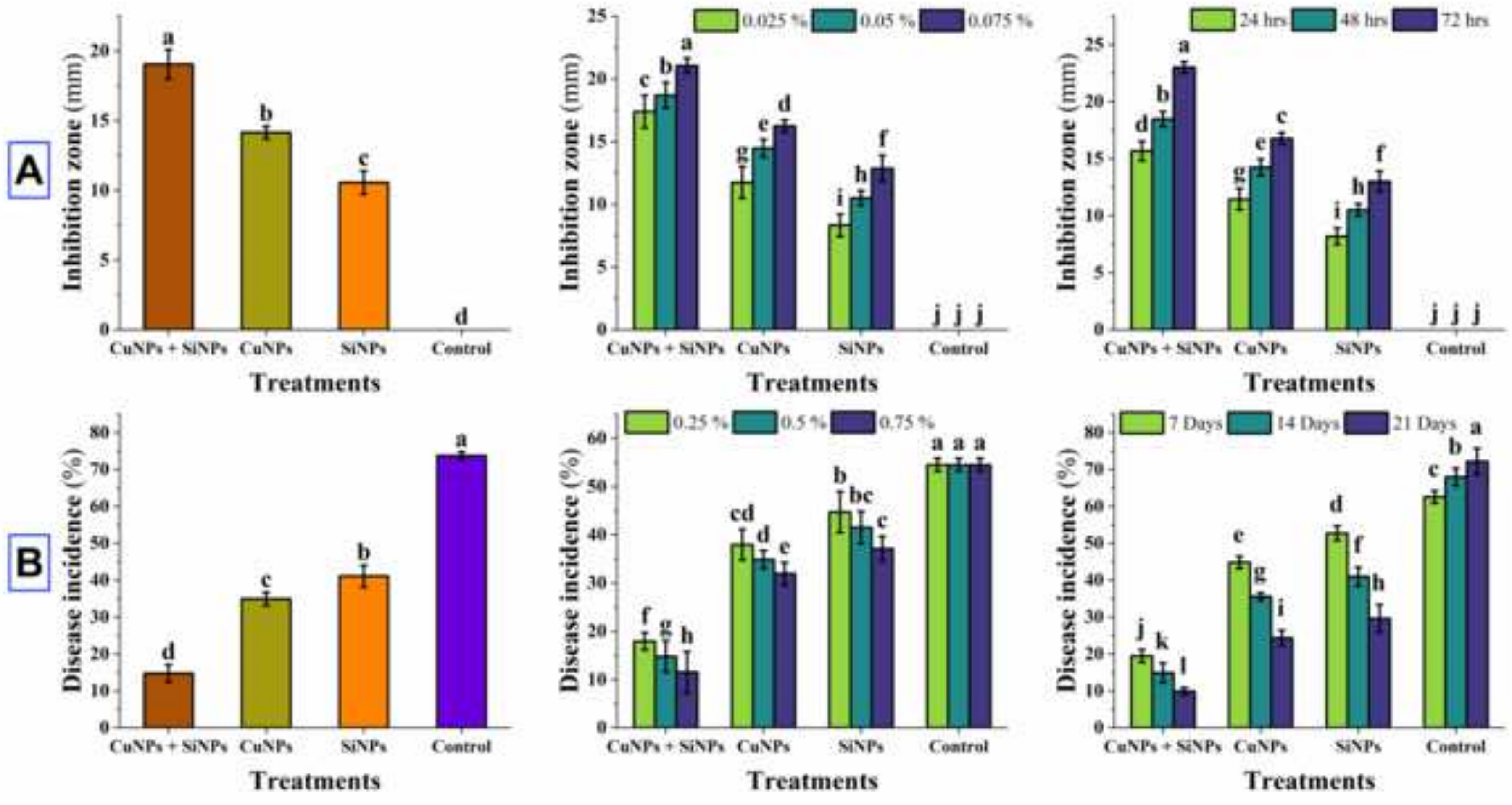

Figure3

Click here to access/download;Figure;Fig 3.tif

C

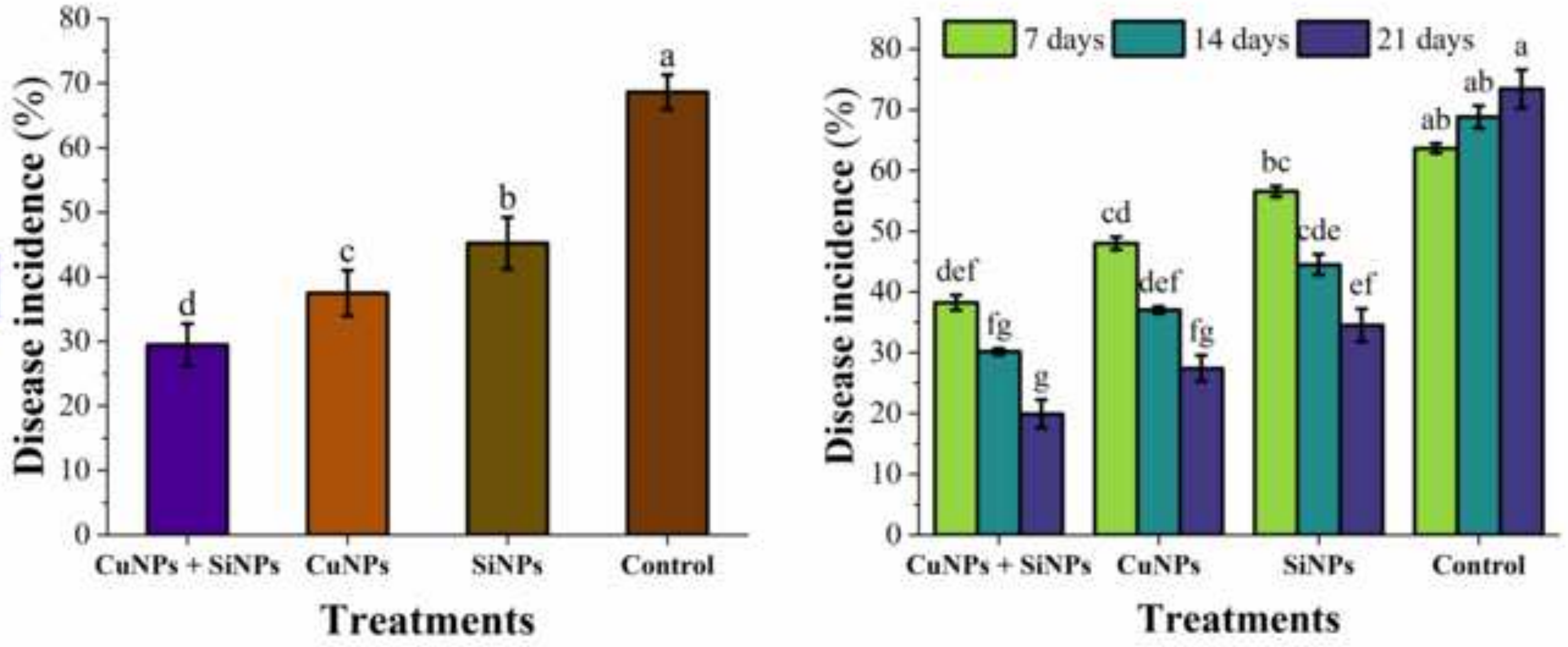

Figure 4

Click here to access/download;Figure;Fig 4.tif

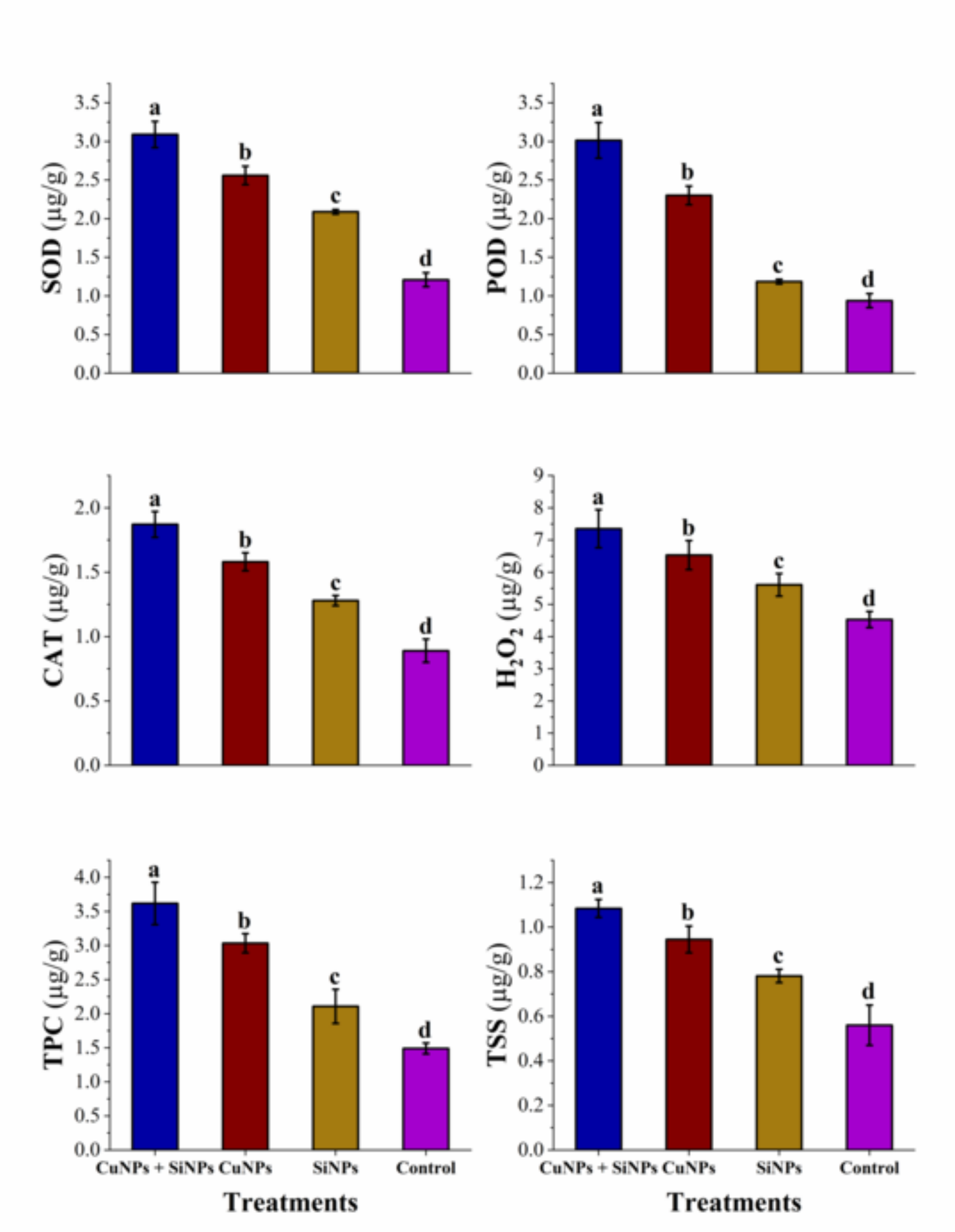

Figure5 A

Click here to access/download;Figure;Fig 5A.tif

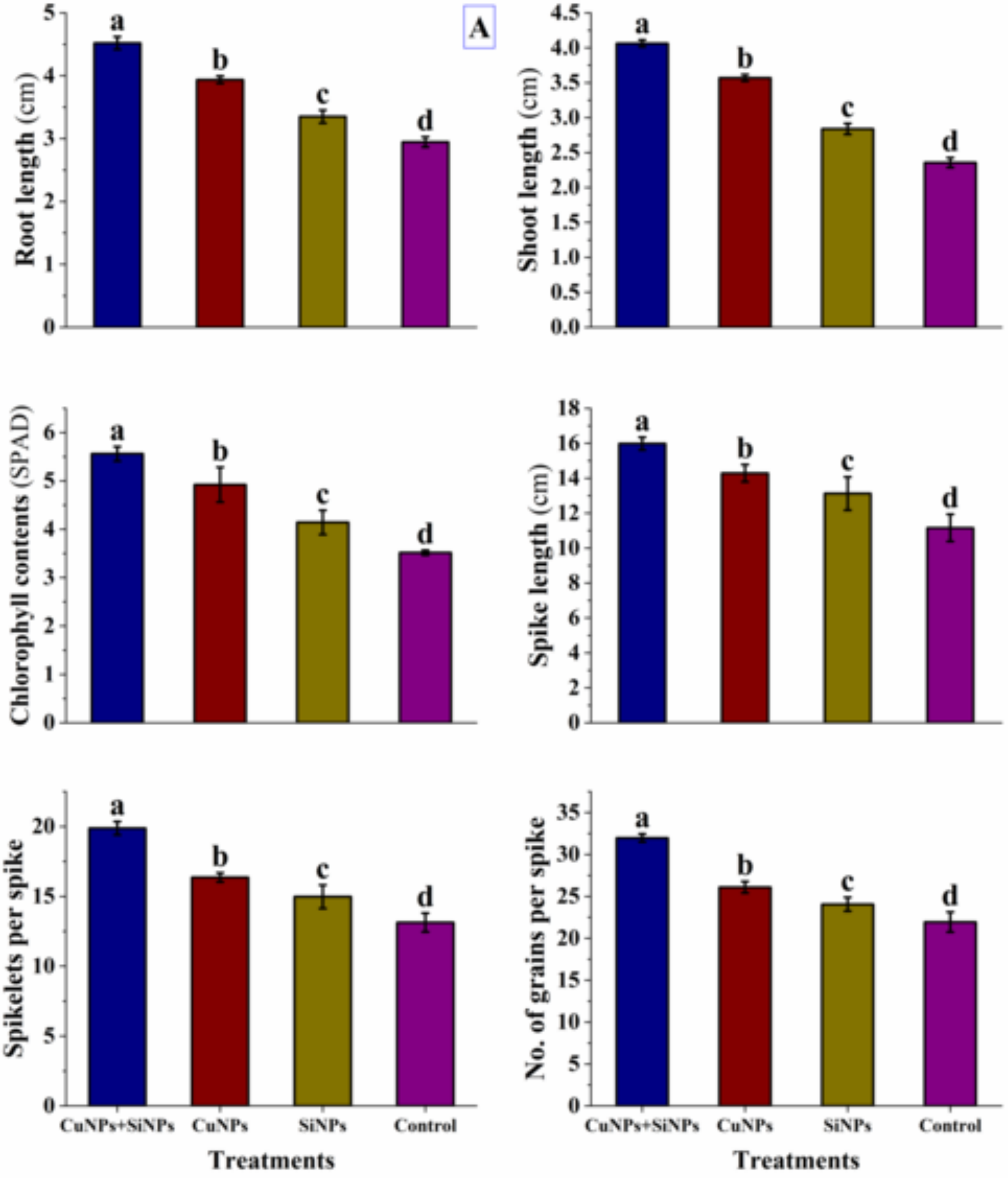

Figure 5B

Click here to access/download;Figure;Fig 5B.tif

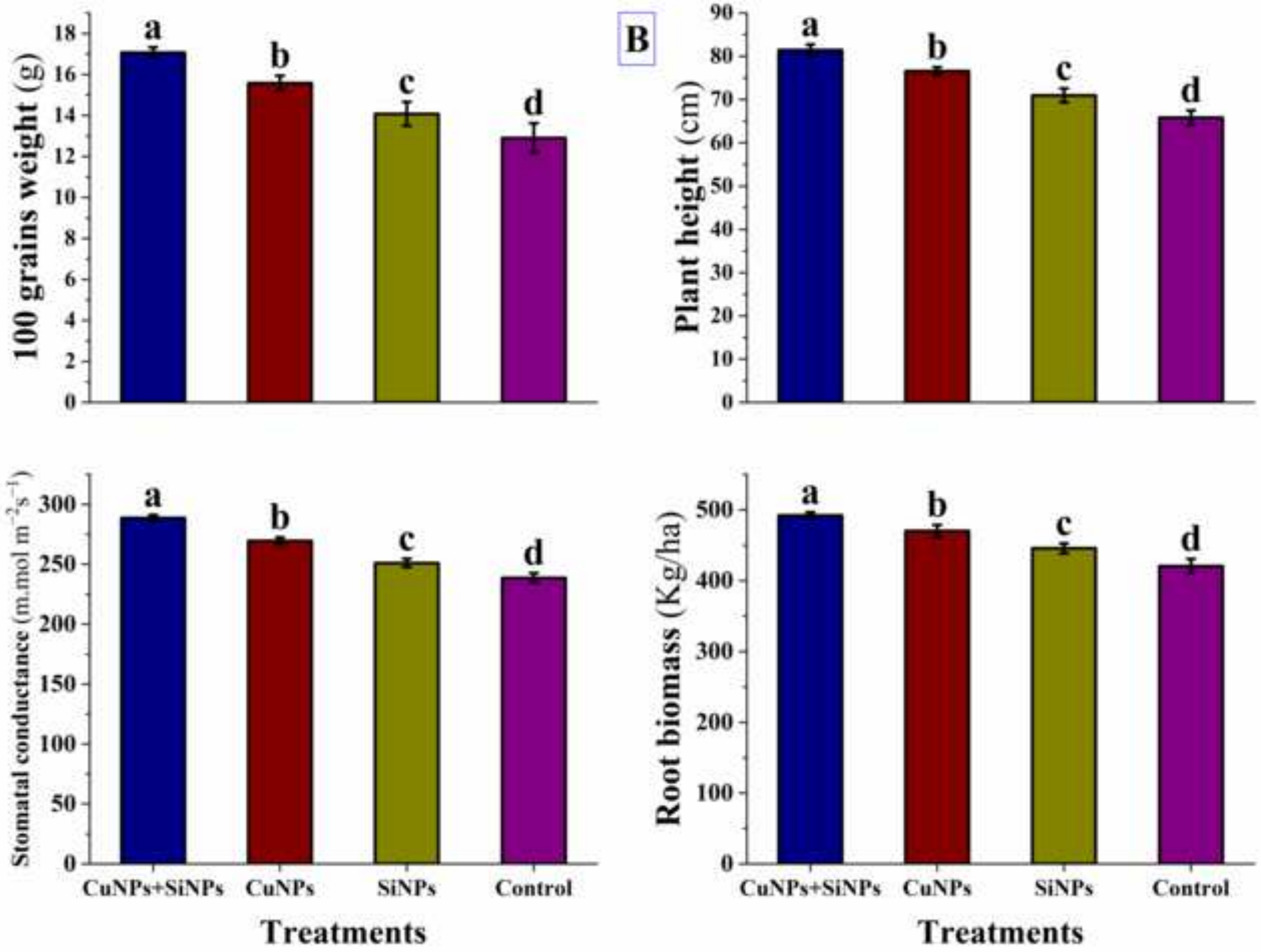

Figure 6

Click here to access/download;Figure;Fig 6.tif

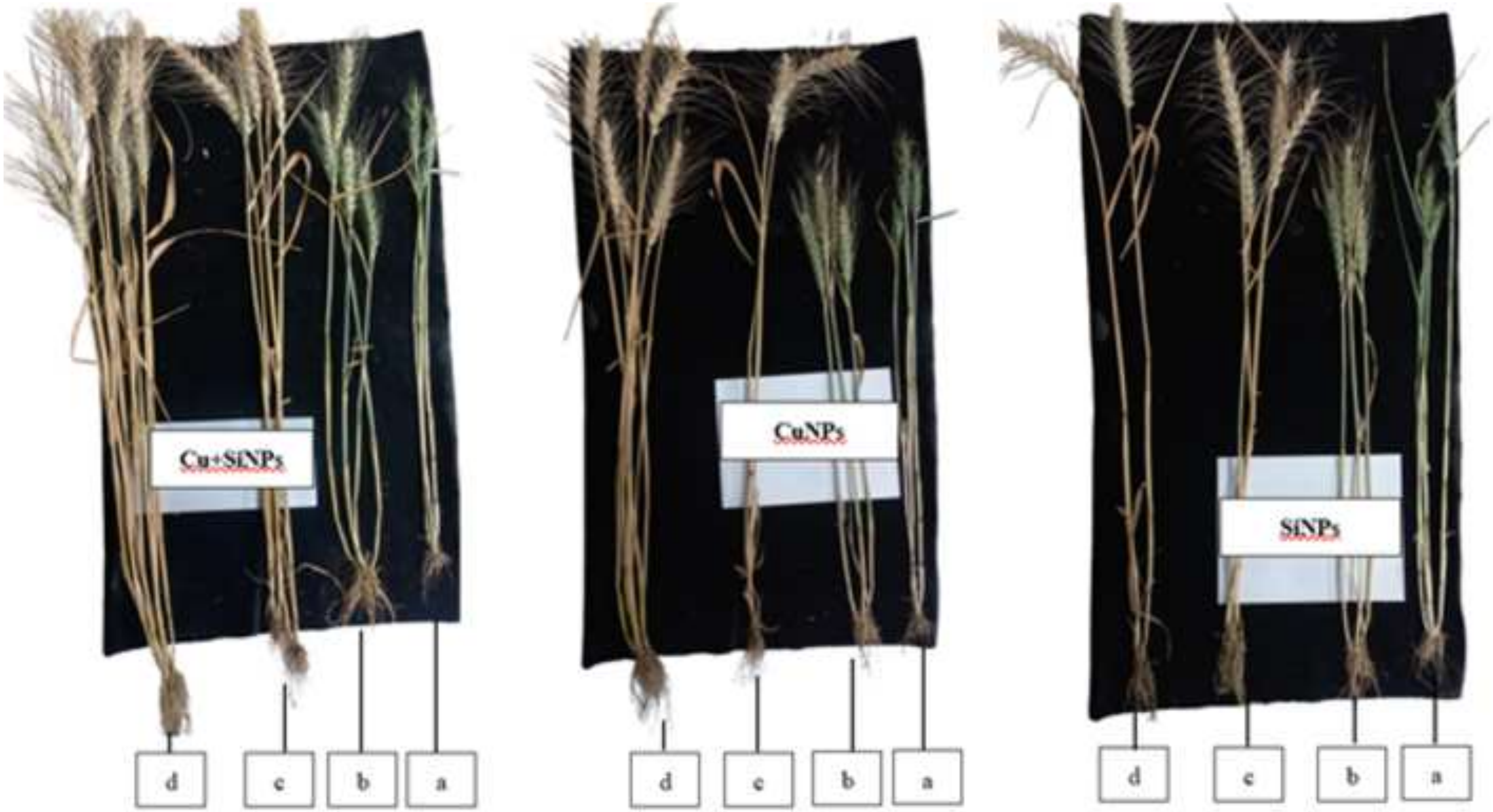

Click here to access/download
Supporting Information
Biochemical data OK.xlsx

Click here to access/download
Supporting Information
Agronomic attributes data OK.xlsx

Click here to access/download

Supporting Information

Evaluation of Nanoparticles OK.xlsx
