# Supplementary material for: Multimodal impact of copper-silicon hybrid nanotools towards bacterial leaf streak, wheat biochemistry and productivity parameters
Source: PLoS One. 2025 Jul 15;20(7):e0327021. doi: 10.1371/journal.pone.0327021 (PMC12262875; doi:10.1371/journal.pone.0327021)
Supplement: Attachment — Submitted filename: PONE-D-24-41835_R3_reviewer.pdf [file pone.0327021.s010.pdf]

PLOS ONE

RESILIENCE OF PARTHENIUM HYSTEROPHORUS-DERIVED COPPER-SILICON HYBRID NANOTOOLS AGAINST BACTERIAL LEAF STREAK AND THEIR IMPACT ON BIOCHEMICAL AND AGRONOMIC TRAITS OF WHEAT

--Manuscript Draft--

**Manuscript Number:**	PONE-D-24-41835R3
**Article Type:**	Research Article
**Full Title:**	RESILIENCE OF PARTHENIUM HYSTEROPHORUS-DERIVED COPPER-SILICON HYBRID NANOTOOLS AGAINST BACTERIAL LEAF STREAK AND THEIR IMPACT ON BIOCHEMICAL AND AGRONOMIC TRAITS OF WHEAT
**Short Title:**	COPPER-SILICON HYBRID NANO TOOLS TOWORDS BACTERIAL LEAF STREAK, BIOCHEMICAL AND AGRONOMIC ATTRIBUTES OF WHEAT
**Corresponding Author:**	Muhammad Atiq, Ph.D<br>University of Agriculture Faisalabad Faculty of Agriculture<br>Faisalabad, Punjab PAKISTAN
**Keywords:**	BLS of wheat, Biochemical profiling, inhibition zone, Xanthomonas translucens pv. undulosa, SOD, POD, Catalase
**Abstract:**	Wheat production is substantially harmed by biotic and abiotic stresses. Among biotic stresses, bacterial leaf streak (BLS) of wheat which is caused by bacterium Xanthomonas translucens pv. undulosa (Xtu) induces crop yield losses up to 10-40% depending upon inoculum level and susceptibility of host. This multi-step investigation encompassed the evaluation of the antibacterial potency of parthenium-mediated CuNPs and SiNPs. Green synthesized Cu-Si nanoparticles were evaluated under lab and greenhouse conditions employing a Complete Randomized Design (CRD) and under field conditions adopting Randomized Complete Block Design (RCBD) alone and in combination. The scanning electron microscopy and X-ray diffraction showed that CuNPs and SiNPs exhibited semi-spherical and spherical morphology with average particle size of 61.49nm and 14.36nm, respectively. Among the tested nanoparticles, maximal inhibition one was expressed by CuNPs + SiNPs (19.06 mm), followed by CuNPs (14.14 mm) and SiNPs (10.56 mm) as compared to control. The least disease incidence under greenhouse (14.75%) and field-scale evaluation (29.46%) was expressed by CuNPs + SiNPs, followed by CuNPs and SiNPs in comparison with control. Moreover, the execution of most effective dosage of CuNPs + SiNPs enhanced the amounts of enzymatic and non-enzymatic antioxidants like SOD, POD, CAT, H2O2, TPC, TSS by 3.09, 3.01, 1.87, 7.35, 3.61 and 1.08 µg/g, respectively in comparison with control. Similarly, most effective dosage of CuNPs + SiNPs increased the yield-related attributes of the wheat plant such as root length (4.52 cm), shoot length (4.063 cm), chlorophyll contents (5.56 SPAD), spike length (15.98 cm), spikelets per spike (19.88) and No. of grains per spike (31.97), 100 grains weight (17.07 g), plant height (81.46 cm), stomatal conductance (288.69 m.mol m−2s−1), and root biomass (492.15 kg/ha). Recent findings emphasize the potential of ecological nanotechnology-based tactics in plant disease management. Furthermore, nanoparticles applications are emphasized for the effective management of disease through enhanced enzymatic and non-enzymatic antioxidant defense system and yield-related attributes of wheat. It is presumed that the application of CuNPs and SiNPs was an eco-friendly tactic for managing the bacterial leaf streak of wheat and enhancing the antioxidant defense system and yield-related attributes of wheat.
**Order of Authors:**	Waqas Ahmad, MSc (Hons)
	Muhammad Atiq, Ph.D
	Nasir Ahmed Rajput
	Rizwana Maqbool, Ph.D
	Muhammad Jamil Shafi, PhD
	Abdul Jabbar

Powered by Editorial Manager® and ProduXion Manager® from Aries Systems Corporation

	Suhail Asad, PhD
	Muhammad Jahanzaib Matloob, MSc (Hons)
	Hassan Jahanzaib Zia, MSc (Hons)
	Muhammad Usama, MSc (Hons)
**Response to Reviewers:**	Reviewer1<br><br>1.The title needs to be clarified.<br><br>A. Incorporated<br><br>2.I highly recommend reviewing the grammar of the paper.<br>A. Grammatical Errors are corrected.<br><br>3. 1.Introduction section;<br>(a)Line 50, start the Intro with reference No. [16], How?<br>(b)The references need to be rearranged.<br><br>A. All the references in the text are rearranged according to your prescription.<br>4. 4.Material and Methods section;<br>(a)Line 106 “ml” change to mL and so on.<br>(b)What specific synthesis techniques are most effective for combining silicon and copper nanoparticles?<br>(c)This method of combination isn’t mentioned.<br>(d)Why did you choose these three concentrations (0.025, 0.05, and 0.075 g).<br>(e)Line 182 “ntreated” changed to untreated<br><br>A. a.Changed to mL and so on.<br>b.Simply the fine powder of both nanoparticles (Cu and Si) was mixed in a beaker and further used for their assessment under laboratory, greenhouse and field conditions.<br>c.It is stated in line 117.<br>d.Nanoparticles cause toxicity to plants at higher concentrations. That’s why lower concentrations were most effective for the best results.<br>e.Incorporated.<br><br>5. 5.Results;<br>Please, add the UV-vis and SEM figures<br><br>A. The SEM figures are attached. UV-vis pictures are not available.<br><br><br>Reviewer 2<br><br>1.Throughout the manuscript check for English typos<br><br>A. Thoroughly proofread was done to remove any English typos errors.<br><br>2.The title is too long, maximum should be 15 words<br><br>A. Short title will not represent the focus of the research paper clearly, that’s why the title is quite longer.<br><br>3. the introduction needs more recent literatures, I suggested some.<br><br>A. As per your suggestions, literature provided is recent. Kindly have a look on it. Most of the references are above the year 2020 despite four references.<br><br>4.the method should be referenced<br>A. Complete methodology is referenced.<br><br>5. the figures and tables presenting the data very well, but the discussion should explain why the clear zone is increasing by the time<br>A. The suggestion is incorporated and explained why the clear zone is increased with

increase in time.

6. Statistical analysis should be done with two ways ANOVA to compare within the time interval

A. The statistical analysis was performed using three ways ANOVA to compare it with time and concentration.
7.The author did response to the comments but all the comments in the attached reviewed manuscript are missing.
Check and address all the comments

8. 1.The author responded partially to the comments, as there are several comments in the attached reviewed manuscript that are not yet addressed in the submitted revised manuscript
A. In this revision, all the relevant comments missing in previous revision are addressed properly with comments section.

Reviewer 3.

1.The authors will need to present the results for the characterization of the nanoparticles before application. The scanning electron microscope for the nanoparticles were not presented.

A. SEM for the characterization of nanoparticles is represented in the figures.

2.Experiments are better presented as three biological replicates rather than three replicates.

A. Suggestions are incorporated.

3. The zone of inhibitions represented on plate was not interpreted statistically.

A. Incorporated.

4. The statistical application presented in the treatment figure 4 and figure 5 should be revisited.

A. Suggestions are incorporated.

A. In this revision, all the relevant comments that were missed in the previous revision are addressed properly with comments section using track changes. Kindly check it.

Reviewer 4.

1.Abstract: The abstract is informative but could benefit from a more concise summary of the results and implications. Abbreviations (CuNPs, SiNPs, Cu-Si, etc.) should be expanded upon first use. Consider highlighting key findings and their significance in a more straightforward manner.

A. Suggestions are incorporated.

2. Introduction: While the introduction provides a solid background, it would be beneficial to include more recent references to strengthen the context of your research. The introduction is not up to date, and proper citations are missing. For example:
(a)Line 48: Pakistan ranks 8th in what? If you are referring to wheat, it is ranked 7th. Confirm this with the USDA and add the latest reference.
(b)Line 50: It appears that your references are not arranged correctly. Citations should start from 1, not 16. Follow the proper rules of citation.
(c)Additionally, clearly stating the research objectives at the end of this section would provide a clearer focus for readers.

A. The introduction is up to date. Kindly have a look on all references. Just 2-3 references are below to year 2020. All the remaining are of year 2020, 2021, 2022,

Powered by Editorial Manager® and ProduXion Manager® from Aries Systems Corporation

2023, and 2024.
a.It is confirmed that Pakistan stands at number 8th with respect to wheat production globally.
b.All the references are rearranged as per their appearance in the text.
c.Objectives are clearly stated at the end of introduction.

3. Methodology: The methods used for synthesizing and testing nanoparticles are well described. However, additional details regarding the controls used in experiments would enhance reproducibility. Clarify how concentrations were determined and provide justification for the selected dosages.

A. The control is referred to as distilled water during the whole experiment. To get three concentrations (0.025, 0.05, and 0.075 %) of NPs, 0.025, 0.05, and 0.075 g of (Cu and Si) powder alone and in combination (Cu + Si) were added in bottle (250 mL) containing 100 mL distilled water separately. The nanoparticles cause toxicity to plants if applied at higher concentrations that's why small dosages are recommended.

4. Results and Discussion: The results are presented clearly, but the discussion could be expanded to include comparisons with existing literature. Discussing potential mechanisms behind observed effects would provide deeper insights into your findings.

A. In the discussion, the current findings are supported by latest studies and compared with the existing literature. The potential mechanism behind the action of both copper and silicon nanoparticles is described very well.

5.Figures: Ensure that all figures and tables are clearly labeled and referenced in the text. Some figures lack sufficient legends (e.g., Fig. 4, 5A, and 4B) explaining their significance. The photo quality is low; please upload high-resolution images (at least 300 dpi).

A. The Fig. 4, 5A and 5B are representing the data very well and their resolution is also at least 300 dpi.

6.Tables: The table format is not suitable for publication. Please use the standard table format.

A. Table is removed from the manuscript emphasizing the formatting of the manuscript.

7. Conclusion: The conclusion summarizes the findings effectively but should also address future research directions or potential applications of your findings in agricultural practices.

A. Future research directions are updated.

8. Statistical Analysis: Please provide more details regarding the statistical methods used for data analysis, including the software and specific tests applied.

A. Statistical analysis is performed by employing ANOVA (Analysis of Variance) with LSD test at $p \leq 0.05$ in software named as "Statistix 8.1". The graphical representation was performed on "Origin 2024b".

9. Side Effects: As parthenium is a causative agent of Parthenium dermatitis, what are the side effects of parthenium on wheat and ultimately on humans?

A. Parthenium dermatitis is one of the main constraints faced by human health as a prolonged exposure to Parthenium hysterophorus L. It can also cause respiratory risks like asthma and bronchitis.

10. 10.The authors have incorporated the suggestions. However, in some comments, the authors did not follow the instructions. For example:
1. Pakistan ranks 7th in wheat production instead of 8th (https://fas.usda.gov/data/production/commodity/0410000).
2. The wheat contribution to agricultural value added and GDP is incorrect. The authors must update their manuscript according to the recent reference (Investigating

	the Impact of Agricultural Credit on Wheat Farming: Evidence from Pakistan, https://doi.org/10.3390/agriculture14122200).<br>I advise the authors to review the above information and verify it with the latest online or published data (if available). The purpose of the revision is to improve and provide globally accepted and authenticated data for publication<br><br>A. All the comments raised during previous review were addressed in a scientific way.<br>1.This comment was also suggested to review and confirm the status of the Pakistan ranking for wheat production. It is confirmed by the data of FAOSTAT-2022 where it is clearly written that Pakistan ranked eighth in wheat production with respect to the globe. You can confirm this with this link of FAOSTAT (https://www.fao.org/faostat/en/#rankings/countries_by_commodity). Kindly check it. It will represent that Pakistan is ranked eighth in production of wheat.<br>2.The agricultural value added by the wheat and its role in GDP of Pakistan is confirmed with the official economic survey of Pakistan under agriculture section. Kindly confirm this using the given link to Economic survey of Pakistan (https://www.finance.gov.pk/survey/chapter_24/2_agriculture.pdf
Additional Information:	
Question	Response
**Financial Disclosure**<br><br>Enter a financial disclosure statement that describes the sources of funding for the work included in this submission. Review the submission guidelines for detailed requirements. View published research articles from *PLOS ONE* for specific examples.<br><br>This statement is required for submission and **will appear in the published article** if the submission is accepted. Please make sure it is accurate.<br><br>**Funded studies**<br>Enter a statement with the following details:<br>• Initials of the authors who received each award<br>• Grant numbers awarded to each author<br>• The full name of each funder<br>• URL of each funder website<br>• Did the sponsors or funders play any role in the study design, data collection and analysis, decision to publish, or preparation of the manuscript?<br><br>Did you receive funding for this work?	The author(s) received no specific funding for this work.
**Competing Interests**<br><br>Use the instructions below to enter a competing interest statement for this	The authors have declared that no competing interests exist.

submission. On behalf of all authors, disclose any competing interests that could be perceived to bias this work—acknowledging all financial support and any other relevant financial or non-financial competing interests.<br><br>This statement is required for submission and **will appear in the published article** if the submission is accepted. Please make sure it is accurate and that any funding sources listed in your Funding Information later in the submission form are also declared in your Financial Disclosure statement.<br><br>View published research articles from *PLOS ONE* for specific examples.<br><br>**NO authors have competing interests**<br><br>Enter: *The authors have declared that no competing interests exist.*<br><br>**Authors with competing interests**<br><br>Enter competing interest details beginning with this statement:<br><br>*I have read the journal's policy and the authors of this manuscript have the following competing interests: [insert competing interests here]*<br><br>* typeset	
**Ethics Statement**<br><br>Enter an ethics statement for this submission. This statement is required if the study involved:<br><br>• Human participants<br>• Human specimens or tissue<br>• Vertebrate animals or cephalopods<br>• Vertebrate embryos or tissues<br>• Field research<br><br>Write "N/A" if the submission does not	NA

Powered by Editorial Manager® and ProduXion Manager® from Aries Systems Corporation

require an ethics statement.<br><br>General guidance is provided below. Consult the submission guidelines for detailed instructions. **Make sure that all information entered here is included in the Methods section of the manuscript.**<br><br>**Format for specific study types**<br><br>**Human Subject Research (involving human participants and/or tissue)**<br>• Give the name of the institutional review board or ethics committee that approved the study<br>• Include the approval number and/or a statement indicating approval of this research<br>• Indicate the form of consent obtained (written/oral) or the reason that consent was not obtained (e.g. the data were analyzed anonymously)<br><br>**Animal Research (involving vertebrate animals, embryos or tissues)**<br>• Provide the name of the Institutional Animal Care and Use Committee (IACUC) or other relevant ethics board that reviewed the study protocol, and indicate whether they approved this research or granted a formal waiver of ethical approval<br>• Include an approval number if one was obtained<br>• If the study involved *non-human primates*, add *additional details* about animal welfare and steps taken to ameliorate suffering<br>• If anesthesia, euthanasia, or any kind of animal sacrifice is part of the study, include briefly which substances and/or methods were applied<br><br>**Field Research**<br><br>Include the following details if this study involves the collection of plant, animal, or other materials from a natural setting:<br>• Field permit number<br>• Name of the institution or relevant body that granted permission	
**Data Availability**	Yes - all data are fully available without restriction

Powered by Editorial Manager® and ProduXion Manager® from Aries Systems Corporation

Authors are required to make all data underlying the findings described fully available, without restriction, and from the time of publication. PLOS allows rare exceptions to address legal and ethical concerns. See the PLOS Data Policy and FAQ for detailed information.<br><br>A Data Availability Statement describing where the data can be found is required at submission. Your answers to this question constitute the Data Availability Statement and **will be published in the article**, if accepted.<br><br>**Important:** Stating 'data available on request from the author' is not sufficient. If your data are only available upon request, select 'No' for the first question and explain your exceptional situation in the text box.<br><br>Do the authors confirm that all data underlying the findings described in their manuscript are fully available without restriction?	
**Describe where the data may be found in full sentences. If you are copying our sample text, replace any instances of XXX with the appropriate details.**<br><br>• If the data are **held or will be held in a public repository**, include URLs, accession numbers or DOIs. If this information will only be available after acceptance, indicate this by ticking the box below. For example: *All XXX files are available from the XXX database (accession number(s) XXX, XXX.*).<br>• If the data are all contained **within the manuscript and/or Supporting Information files**, enter the following: *All relevant data are within the manuscript and its Supporting Information files.*<br>• If neither of these applies but you are able to provide **details of access elsewhere**, with or without limitations, please do so. For example:	All relevant data are<br>within the manuscript and its Supporting<br>Information files.

Data cannot be shared publicly because of [XXX]. Data are available from the XXX Institutional Data Access / Ethics Committee (contact via XXX) for researchers who meet the criteria for access to confidential data.<br><br>*The data underlying the results presented in the study are available from (include the name of the third party and contact information or URL).*<br>• This text is appropriate if the data are owned by a third party and authors do not have permission to share the data.<br><br>* typeset	
Additional data availability information:	

Powered by Editorial Manager® and ProduXion Manager® from Aries Systems Corporation

Manuscript Click here to access/download;Manuscript;Manuscript (5).docx

RESILIENCE OF PARTHENIUM HYSTEROPHORUS-DERIVED COPPER-SILICON HYBRID NANOTOOLS AGAINST BACTERIAL LEAF STREAK AND THEIR IMPACT ON BIOCHEMICAL AND AGRONOMIC TRAITS OF WHEAT

WAQAS AHMAD[1], MUHAMMAD ATIQ[1]*, NASIR AHMED RAJPUT[1], RIZWANA MAQBOOL[2], JAMIL SHAFI[3], ABDUL JABBAR[4], AHMAD NAWAZ[1], MUHAMMAD JAHANZAIB MATLOOB[1], HASSAN ZIA[1], MUHAMMAD USAMA[1]

[1]Department of Plant Pathology, Faculty of Agriculture, University of Agriculture, Faisalabad, Pakistan.
[2]Department of Plant Breedings and Genetics, Faculty of Agriculture, University of Agriculture, Faisalabad, Pakistan.
[3]Department of Plant Pathology, Constituent College Depalpur, Okara, University of Agriculture, Faisalabad, Pakistan.
[4]Department of Agronomy, Faculty of Agriculture, University of Agriculture, Faisalabad, Pakistan.

* Corresponding author

E-mail: muhammad.atiq@uaf.edu.pk (MA)

Abstract

Wheat production is substantially harmed by biotic and abiotic stress. Among biotic stresses, bacterial leaf streak (BLS) of wheat caused by bacterium *Xanthomonas translucens* pv. *undulosa* (*Xtu*) induces crop yield losses up to 10-40%. This multi-step investigation encompassed the evaluation of the antibacterial potency of parthenium-mediated copper nanoparticles (CuNPs) and silicon nanoparticles (SiNPs). Green synthesized Cu-Si nanoparticles were evaluated under lab and greenhouse conditions employing a Complete Randomized Design (CRD) and under field conditions adopting Randomized Complete Block Design (RCBD) alone and in combination. The scanning electron microscopy and X-ray diffraction showed that CuNPs and SiNPs exhibited semi-spherical and spherical morphology with average size of 61.49nm and 14.36nm, respectively. Among the tested nanoparticles, maximal inhibition one was expressed by CuNPs + SiNPs (19.06 mm), followed by CuNPs (14.14 mm) and SiNPs (10.56 mm) as compared to control. The least disease incidence under greenhouse (14.75%) and field-scale evaluation (29.46%) was expressed by combined treatment CuNPs + SiNPs, followed by single treatment CuNPs and SiNPs in comparison with control. Moreover, the execution of the most effective dosage of CuNPs + SiNPs enhanced the amounts of enzymatic and non-enzymatic antioxidants like SOD, POD, CAT, H_2O_2, TPC, TSS by 3.09, 3.01, 1.87, 7.35, 3.61 and 1.08 µg/g, respectively. Similarly, most effective dosage of CuNPs + SiNPs increased the yield-related attributes of the wheat plant such as root length (4.52 cm), shoot length (4.063 cm), chlorophyll contents (5.56 SPAD), spike length (15.98 cm), spikelets per spike (19.88) and Number of grains per spike (31.97), 100 grains weight (17.07 g), plant height (81.46 cm), stomatal conductance (288.69 m.mol $m^{-2}s^{-1}$), and root biomass (492.15 kg/ha). Recent findings emphasize the potential of ecological nanotechnology-based tactics in plant disease management. Furthermore, nanoparticles applications with CuNPs and SiNPs were an eco-friendly tactic for managing the bacterial leaf streak of wheat and enhancing the antioxidant defense system and yield-related attributes of wheat.

Introduction

Pakistan ranks eighth in production, yielding 31.4 million tonnes over an area of 9.4 million hectares. Wheat contributes 9.0 % to agricultural value-added and 2.2 % to GDP of Pakistan [1]. It is crucial for diets, furnishing key nutrients such as proteins, carbohydrates, vitamins, fats, minerals and nutrients [2, 3,]. It aids in treating heart diseases, constipation, cancer, obesity, and diabetes [2]. Wheat by-products (wheat bran, white flour, bread flour, cake flour, gluten flour, semolina, farina) are used in animal feed, bioethanol, baking, cosmetics, and pharmaceuticals [4].

Different biotic (fungi, bacteria, nematode, virus) and abiotic (drought, temperature, salinity, floods, radiations, pollutants) factors are responsible for the reduced production impacting qunatity and quality of crop [5]. Among the biotic factors, bacterial leaf streak (BLS) which is caused by the gram-negative *Xanthomonas translucens* pv. *undulosa* is a major global threat to wheat production [6, 7]. Optimal growth was noted at 28-30°C, but it terminates at 36°C, particularly when moisture levels are high [8]. The disease can cause up to 10-40% yield losses worldwide, dependent on inoculum levels and host susceptibility [9]. The pathogen also endures on crop debris, soil, and weeds, penetrating the plants through stomata, wounds and hydathodes. Inside the plant, the pathogen multiply in the mesophyll tissues producing exopolysaccharides which lead to biofilm formation [10]. *Xtu* uses type III effectors (T3E) via a type III secretion system (T3SS) to initiate the disease symptoms [11] which appeared as water-soaked lesions that gradually turn yellow, brown, and necrotic. These lesions eventually ooze a honey-like exudate, which spreads under humid conditions and forms a thin, transparent layer [12].

Different management approaches are in practice to cope the bacterial leaf streak of wheat. The most efficient approach for managing the disease is the employmentof resistant varieties. However, under favorable conditions, even these varieties become susceptible [13].

Excessive use of chemicals for disease management can disrupt plant functions and cause toxicity, leading to a range of health issues and organ impairments [14]. Considering the hazardous effects of chemicals and the limitations of other control measures, advanced strategies such as green nanotechnology are essential for effective disease management [15]. This emerging technique uses nanoparticles to treat plant bacterial disease being preferred over chemical and physical methods due to its cost-effectiveness and environmental friendliness [16, 1[illegible] Nanoparticles (1-100 nm) are used in small doses to effectively inhibit microbial enzymes, damage essential molecules, cause cell wall damage, and stimulate oxidative stress which led to microbial death [18, 19]. Use of weeds for the integration of green-based nanoparticles offer an eco-friendly approach to manage plant diseases [2[illegible] Researchers have developed green-based nanoparticles, using weed extracts to control bacterial plant diseases [[illegible] Copper nanoparticles (Cu-NPs) demonstrated strong antibacterial activity against *Xanthomonas campestris* [22], and *Xanthomonas campestris* pv. *vesicatoria* [23]. Copper and silver nanoparticles synthesized from *Eucalyptus globulus* leaves were effective against *Xanthomonas citri* [13], and Cu-NPs also inhibited *Ralstonia solanacearum* [24]. Similarly, silicon nanoparticles (Si-NPs) proved effective against *Pectobacterium carotovorum* and *X. campestris* pv. *carotae* in carrots [25, 26].

Nanoparticles (NPs) improve the wheat biochemistry by enhancing the nutrient acquisition, photosynthetic efficacy, and inducing stress resistance [27]. NPs regulate the enzymatic activity of antioxidants activity such as SOD, POD, and CAT that scavenge the ROS and combat the oxidative damage [28]. NPs also regulate the non-enzymatic antioxidants acitvity like H_2O_2, TPC, and TSS that make sure the strengthening of defense responses and reducing oxidative stress, and in result, ensuring the better overall plant health and resilience [29]. NPs not only suppress the harmful effects of pathogens but also promote the plant growth metrics [30]. So, the current research effort was directed to investigate the antibacterial function

of *Parthenium hysterophorus* mediated Cu-Si hybrid nanotools towards bacterial leaf streak of wheat and its effect on the physiology and biochemistry of wheat plant.

Materials and methods

Synthesis of weed-based nanoparticles (NPs)

Parthenium (*Parthenium hysterophorus* L.) was collected from the Agronomy Department Research Area, and was shade-dried for about 4-5 days. After this, it was sun-dried over 3 days. Then, it was kept in a dry oven (101-1AB) to remove remaining moisture at 65 ℃ for 4 hours. After oven drying, pestle and mortar was employed to grind the weed into fine powder. Then, powder was filtered by using muslin cloth to obtain a fine powder without impurities. 20 g of this fine powder was added in a beaker (250 mL) containing 100 mL methanol, covered with an aluminium foil and kept under darkness over a period of 24 hours (for effective extraction, deterrence of photodegradation and facilitates the uniform interaction). After this, the solution was stirred in a magnetic stirrer at 70 ℃ for about 15 minutes (for uniform dissolution and helped in boosting the reaction). Then, filtration of the solution was done through Whatman's Filter Paper No. 41. For the preparation of Silicon and Copper nanoparticles, 17g sodium metasilicate (Na_2SiO_3) and 17g copper sulphate ($CuSO_4$) was added in the filtrate separately and put it on magnetic stirrer (C-MAG HS 4) for mixing of solution and then placed it in ultrasonic cleaner (VEVOR) at 60°C for breaking the bond for 1 hour. To obtain Si and Cu, nanoparticles in powder form, the solution was placed in a furnace oven (Carbolite PF60, higher temp. processing, uniform heating under controlled conditions) [13]. For the preparation of hybrid nanoparticles, both silicon and copper nanoparticles were mixed.

Characterization of parthenium mediated nanoparticles

The characterization of the parthenium mediated nanoparticles was performed by adopting different techniques as performed by [31].

UV–visible spectroscopy

The biological reduction of the solution was pursued by determining its absorbance with a UV-visible spectrophotometer (V-750) over the range of 200-800 nm. 1 mL of the sample was used and 1mL of distilled water was used as control.

X-ray diffraction spectroscopy

The average size of nanoparticles was determined by employing diffractometer (Philips X-Pert MMP). The P Rofit software was employed to control the diffractometer on a computer, with molybdenum radiation (MoK) used as the source (λ = 0.7093 A). The Mo-tube operated at 50 kV and 40 mA to facilitate the bombardment process.

Scanning electron microscopy

The morphological characteristics of the nanoparticles synthesized through parthenium extract was assessed by employing scanning electron microscopy (SEM). For SEM analysis, A 1 mg sample was deposited onto carbon-coated grids and imaged at different magnifications for the assessment of its morphological characters.

In-vitro assessment of green synthesized NPs against *Xanthomonas translucens* pv. *undulosa*

The inhibition zone technique was used for the assessment of green synthesized nanoparticles (copper and silicon) from weed (*Parthenium hysterophorus* L.) against *Xanthomonas translucens* pv. *undulosa* under laboratory conditions. For this purpose, one litre bacterial growth media (NA) was prepared in the Laboratory. To get three concentrations

(0.025, 0.05, and 0.075 %) of NPs, 0.025, 0.05, and 0.075 g of (Cu and Si) powder alone and in combination (Cu + Si) were added in bottle (250 mL) containing 100 mL distilled water separately. After that, NA medium was poured into 9 cm Petri plates and allowed to solidify. Through autoclaved cotton swab, bacterial culture was streaked on Petri plates, and this whole procedure was performed within a laminar flow cabinet (RTVL1312, UK) to prevent the contamination. Circular pieces of the filter paper (1 cm) were cut and sterilized in an autoclave (Robus Technologies, RTA85) at pressure of 15 PSI and 121 °C for 15 minutes. Then, these pieces of filter paper were dipped into formulated concentrations (0.025, 0.05, and 0.075 %) of NPs (Cu and Si) alone and in combination. These dipped filter paper pieces were placed in the centre of the Petri plates having a culture of *Xanthomonas translucens* pv. *undulosa*. In case of control treatment, filter paper pieces were dipped in distilled water rather than in nanoparticle formulations. The plates were wrapped with paraffin and incubated (Heraeus) at 26 ± 2 °C for optimal growth of bacterium.

The inhibition zone was measured by employing digital vernier calliper (500-196, Mitutoyo) after 24, 48, and 72 hours [13]. This trial was designed under Complete Randomized Design (CRD) adopting three replications of each treatment.

In-vivo **evaluation of green synthesized NPs against bacterial leaf streak of wheat**

A field experiment was conducted following the methodology of [13] with trivial adjustments at the Department Research Area. PBG Line 3 was sown employing Randomized Complete Block Design (RCBD) by upholding the P-P and R-R spacing at 10-25 cm and 10-30 cm, respectively for better growth and development. Four treatments (CuNPs + SiNPs, CuNPs, SiNPs, and Control) with three replications of each treatment were evaluated against bacterial leaf streak of wheat. Aqueous suspension of the bacterium *Xtu* was prepared from

culture grown for 48 hours and employing spectrophotometer (Hitachi U-2001, 121003), bacterial concentration @ $1x10^8$ CFU/mL was determined.

The bacterium *Xtu* was artificially introduced in wheat plants by using spray method at the stage of 4-5 leaves. This was done early in the morning when maximum number of stomata were opened in the daytime. After the typical symptom's expression, most effective concentration (0.75 %) of nanoparticles was sprayed in the field while, in the case of control, only distilled water was sprayed on plants. Data regarding the incidence of the bacterial leaf streak was recorded after 7, 14, and 21 days using the formula given below,

$$\text{Disease incidence (\%)} = \frac{No.\ of\ Diseased\ Plants}{Total\ No.\ of\ Plants\ Observed}\ x\ 100 \quad [32]$$

Alterations in the biochemical profiling of wheat leaves after application of nanoparticles

Both treated and untreated wheat plants were collected from the field, and their leaves were cut into small pieces. A 0.5 g leaf sample was ground by employing a sterilized pestle and mortar with potassium phosphate buffer (pH 5). Then, the mixture was centrifuged (TGL-16A) for 5 minutes at 12000 revolutions per minute (rpm). The resulting supernatant was used for advanced biochemical analysis.

Determination of SOD (Superoxide dismutase) from treated and untreated wheat leaves

For this purpose, a reaction mixture containing 200 µL methionine, 100 µL enzyme extract, 200 µL triton X, 500 µL potassium phosphate buffer (pH 5), and 100 µL NBT (as an indicator for superoxide radicals $O_2^{\bullet-}$) with 800 µL distilled water was prepared. It was kept under UV light for 15 minutes. Absorbance was recorded after the addition of 100 µL riboflavin employing spectrophotometer (BioTek, 800TS) at 560 nm wavelength [33].

Determination of POD (Peroxidase) from treated and untreated wheat leaves

For the determination of POD from treated and untreated wheat leaves, a reaction mixture consisting of 20 mM guaiacol, 40 mM H_2O_2 (100 µL), 100 µL enzyme extract, and 800 µL potassium phosphate buffer (pH 5) was prepared. Absorbance was recorded employing spectrophotometer (BioTek, 800TS) at frequency of 470 nm.

Determination of CAT (Catalase) from treated and untreated wheat leaves

A reaction mixture (100 µL enzyme extract and 100 µL of 5.9 mM H_2O_2) was prepared to determine the concentration of catalase in wheat leaves. A spectrophotometer (BioTek, 800TS) was employed to record the absorbance at 240 nm.

Determination of H_2O_2 (Hydrogen peroxide) from treated and untreated wheat leaves

Leaves of treated and untreated plants were collected and sliced into small-sized pieces. A 50 mg leaf sample was taken along with trichloro acetic acid buffer (enhance extraction efficiency, stabilize and prevent degradation of H_2O_2), grinded, and then centrifuged (TGL-16A) at 4°C for 15 minutes at 12000 rpm (stability of sample and preservation of enzymatic content). Then the solution was filtered and treated with potassium phosphate buffer (pH 7) followed by potassium iodide buffer. The final mixture was placed in a digital incubator (RTI-250) for 5 minutes providing a controlled environment for optimal reaction conditions and by using microplate reader (BioTek, 800TS), absorbance was recorded at frequency of 390 nm.

Determination of TPC (Total phenolic contents) from treated and untreated wheat leaves

100 µL enzyme extract was prepared to determine the TPC from treated and untreated wheat leaves. A reaction mixture was prepared consisting of 800 µL Na_2CO_3 (700 mM) and 200 µL of 10% Folin-Ciocalteu (F-C) reagent and allowed to react for 1 hour. Using microplate

reader (Biotek, 800TS), reading was recorded at frequency of 765 nm.

Determination of TSS (Total soluble sugars) from treated and untreated wheat leaves

The 100 mg sample was subjected to hydrolysis through 5 mL of 2.5 N hydrochloric acid for 3 hours in water bath (HT, XMTD-204), then neutralized with sodium carbonate (Na_2CO_3) until bubbling ceased, and finally cooled at at room temperature. A solution of 100 mL was prepared and processed for centrifugation (TGL-16A). 0.5 µL resultant supernatant along with aliquots @ 1 mL was obtained and used for more studies. Special values were developed by using 0.20, 0.4, 0.6, 0.8, and 1 mL as operational values and 0 represented as a blank. To make the volume more than one mL, distilled water was used, and anthrone reagent @ 4 mL was added. For heating the solution, a water bath (HT, XMTD-204) which allows the consistent and controlled heating during hydrolysis without evaporation was used, and absorbance was measured employing spectrophotometer (BioTek, 800TS) at 630 nm [34].

Assessment of impact of NPs on the agronomic attributes of wheat

Root length, shoot length, spike length and overall plant height were measured by manual method using measuring tape (HV-1455) in cm. SPAD (SPAD-502 Plus) meter was used for the precise determination of chlorophyll contents in the leaves of wheat plant by clamping it gently on the leaf. By detaching the spike from the stem, spikelets were counted manually. Total no. of grains per spike were counted precisely by manual method after the threshing process. For the determination of 100 grains weight, 100 grains were counted and then weighed on a weighing balance (SF-400A) in grams. The porometer (AP4 Porometer) was used to determine the stomatal conductance of the wheat leaves by gently attaching the porometer sensor's head to the leaf. It was recorded in mmol m^2s^{-1}. While, for the determination of the root biomass, root samples were collected washed with tap water to remove soil particles.

After this, these are allowed to air dry and then kept in dry oven (101-1AB) for 65 ℃ for 48-72 hours until all water content is removed, and only dry biomass is left behind. Then these were weighed on a digital weighing balance (NL, 7017 X) and recorded in kg ha^{-1}.

Statistical Analysis

The effects of copper and silicon nanoparticles on the inhibition zone of *Xtu*, BLS disease incidence, alterations in biochemical profile, and agronomic attributes of wheat plants were tested using analysis of variance (ANOVA) with LSD test at $p \leq 0.05$ employing Statistix 8.1 [35]. The graphical representation of the data was performed on Origin 2024b software.

Results

Characterization of nanoparticles

The sub-microscopic images of copper and silicon nanoparticles by scanning electron microscopy (SEM) showed that CuNPs exhibited semi-spherical morphology and SiNPs expressed spherical shape. While X-ray diffraction (XRD) analysis of CuNPs and SiNPs showed that NPs expressed crystalline nature exhibiting average particle size less than 100 nm as shown in Fig 1.

Fig 1. SEM of green-synthesized copper and silicon nanoparticles

Inhibition zone and Disease incidence of bacterial leaf streak of wheat

Maximum inhibition zone was expressed by combination of CuNPs + SiNPs (19.06 mm), followed by CuNPs (14.14 mm) and SiNPs (10.56 mm) as compared to control. In case of interaction between T×C and T×D, CuNPs + SiNPs was the most effective followed by CuNPs and SiNPs in comparison with control (Fig 2 and 3A). Under greenhouse conditions,

minimum disease incidence was expressed by CuNPs + SiNPs (14.75 %), followed by CuNPs (34.92 %) and SiNPs (41.11 %) as compared to control. Interactions between T×C and T×D expressed that CuNPs + SiNPs exhibited minimum disease followed by CuNPs and SiNPs as compared to control (Fig 3B). While under field scale evaluation of nanoparticles, results revealed that CuNPs + SiNPs expressed minimum disease incidence followed by CuNPs and SiNPs as compared to control. T×D interaction with most effective concentration revealed that CuNPs + SiNPs expressed minimum disease incidence, followed by CuNPs and SiNPs (Fig 4).

Fig 2. Impact of copper and silicon nanoparticles on the Inhibition zone (mm) of *Xtu* in relation to different concentrations.

Fig 3. Impact of copper and silicon nanoparticles on Inhibition zone (mm) (A), Disease incidence (%) under greenhouse (B). Small letters represent the statistical significance using LSD test at $p \leq 0.05$.

Fig 4. Impact of copper and silicon nanoparticles on Disease incidence (%) under field conditions. Small letters represent the statistical significance using LSD test at $p \leq 0.05$.

Estimation of alterations in the biochemical profiling of wheat

All enzymatic and non-enzymatic antioxidants activity was enhanced substantially after the application of copper and silicon nanoparticles alone and in hybrid. Application of most effective concentration of CuNPs + SiNPs enhanced the activity of SOD (3.09), POD (3.01), CAT (1.87), H_2O_2 (7.35), TPC (3.61), TSS (1.08) µg/g followed by CuNPs (2.56, 2.30, 1.58, 6.53, 3.03, 0.94) µg/g and SiNPs (2.09, 2.1.18, 1.28, 5.60, 2.10, 0.781) µg/g, respectively as compared to control (Fig 5).

Fig 5. Determination of SOD, POD, CAT, H_2O_2, TPC and TSS (µg/g) from treated and untreated wheat leaves after the application of nanoparticles. Small letters represent the statistical significance using LSD test at $p \leq 0.05$.

Assessment of copper and silicon nanoparticles on the agronomic attributes of wheat

All the agronomic attributes of wheat were augmented substantially after the application of copper and silicon nanoparticles alone and in hybrid. Application of most effective concentration of CuNPs + SiNPs expressed the maximum root length (4.52 cm), shoot length (4.063 cm), chlorophyll contents (5.56 SPAD), spike length (15.98 cm), spikelets per spike (19.88) and No. of grains per spike (31.97), followed by CuNPs and SiNPs in comparison with control as shown in Fig 6A. Similarly, CuNPs + SiNPs expressed the maximum 100 grains weight (17.07 g), plant height (81.46 cm), stomatal conductance (288.69 m.mol $m^{-2}s^{-1}$), and root biomass (492.15 kg/ha), followed by CuNPs and SiNPs in comparison with control as shown in Fig 6B. Impact of nanoparticles on the yield-related aspects of plant are shown in Fig 7.

Fig 6A. Impact of copper and silicon nanoparticles on root length (cm), shoot length (cm), chlorophyll contents (SPAD), spike length (cm), spikelets per spike and No. of grains per spike. Small letters correspond to the statistical significance using LSD test at $p \leq 0.05$.

Fig 6B. Impact of copper and silicon nanoparticles on 100 grains weight (g), plant height (cm), stomatal conductance (m.mol $m^{-2}s^{-1}$) and root biomass (kg/ha). Small letters correspond to the statistical significance using LSD test at $p \leq 0.05$.

Fig 7. Impact of nanoparticles on the yield-related attributes of wheat in relation to different concentrations 0.25 (b), 0.50 (c), 0.75% (d) of nanoparticles as compared to control (a).

Discussion

Excessive use of chemicals for disease management can disrupt plant functions and causes toxicity, leading to a range of health issues and organ impairments [14]. So, considering

the hazardous effects of chemicals and the limitations of other control measures, advanced strategies such as green nanotechnology are essential for effective disease management [15]. The current research efforts were focused on the antibacterial efficacy of *Parthenium hysterophorus* mediated copper and silicon nanoparticles and their impacts on the biochemical profiling and yield-related attributes of the wheat for the effective management of BLS of wheat. The results revealed that hybrid (Cu + Si) nanoparticles expressed maximal inhibition zone under laboratory conditions, while minimum disease incidence in greenhouse and field conditions, followed by CuNPs and SiNPs as compared to control. Findings of the current research are encouraged by the outcomes of [36] who evaluated nanoparticles (Cu, MgO, ZnO) NPs, and concluded that CuNPs have the highest antibacterial potency against *Xanthomonas translucens* pv. *undulosa*. Results of this research are endorsed by the results of [27, 37, 38, 39] who observed the antimicrobial activity of CuNPs. The expansion in the clear zone was observed in the petri plates by increasing time. This was due to the sustained release of active agents (Cu^{2+}), diffusion and interaction of NPs within the medium. The nanoparticles were characterized by employing UV-visible spectroscopy, scanning electron microscopy, and X-ray diffraction spectroscopy. The results revealed that copper nanoparticles exhibited the light brown to dark green color, semi-spherical shape with average particle size of 61.49 nm. These findings are in line with the work of [40, 31, 41], who performed the characterization of CuNPs synthesized from *Parthenium hysterophorus*. Similarly, silicon nanoparticles exhibited the spherical shape with the average particle size of 14.36 nm and these outcomes are in agreement with the findings of [42, 43], who reported the average size and shape of the SiNPs.

Copper and silicon nanoparticles have unique mechanisms for their uptake by the plants and suppressing the bacterial pathogens. In case of copper nanoparticles (CuNPs), they accumulate on the bacterial cell membrane and release Cu^{2+} ions that interact with carboxyl as well as sulfhydryl groups in the peptidoglycan layer [44]. CuNPs enter the host plant through

endocytosis [45]. Upon entry into the host plant, these Cu^{2+} ions lead to the generation of reactive oxygen species (ROS), lipid peroxidation resulting in the interference with cell biomolecules, the protein functioning [46] and disrupt the ATP production, results in the disruption of the DNA, which leads to death of the bacterium [47].

Plants get SiNPs (5-20 mm) through endocytosis from roots and invade into the xylem tissues, translocated to different organs of the plant and stored in the shoots [48]. After accumulation, nanoparticles form a second layer of cuticle-Si, which strengthen the physical barriers against pathogenesis [49]. These changes in plants make it more difficult for the pathogens to invade and cause disease. Concurrently, SiNPs directly inhibit the pathogen growth by rupturing cell membranes which led to the spillage of cell contents resulting in cell death [26].

When plants are infected with *Xanthomonas* spp., then, a prominent alteration in the antioxidant defense enzymes like SOD, POD, CAT, and non-enzymatic antioxidants such as TPC, TSS, H_2O_2 was noted [50]. A surge in the induction of reactive oxygen species was experienced after the infection, which leads to an oxidative burst in the wheat plants [51]. Imbalance in the oxidants and antioxidants also resulted in oxidative stress which potentially damaged the plant cells. Higher levels of ROS resulted in the localized cell death and induce substantial tissue damage [52]. These combined consequences lead to the reduction in plant health and vigor which resulted in the limited growth and yield of plants. So, a most pressing need is to regulate the antioxidant defense system of wheat, so that they can exhibit the best growth and development [53]. In this study, the progressive alterations in the enzymatic antioxidants (SOD, POD, CAT) and non-enzymatic antioxidants (TPC, TSS, H_2O_2) from the treated and untreated wheat leaves after the application of copper and silicon nanoparticles alone and in hybrid was determined. The results revealed that Cu-Si hybrid nanoparticles expressed the maximum amounts of SOD, POD, CAT, TSS, TPC, H_2O_2 followed by copper

nanoparticles and silicon nanoparticles in comparison with control. The application of the nanoparticles augmented the level of SOD, POD and CAT through controlled ROS production, directly interacting with enzymes, improving cellular environment, and upregulation of enzymatic genes expression which contributed to a more vigorous and effective antioxidant response, aiding the plants to survive under oxidative stress and make better its resilience to pathogens [43]. Superoxide dismutase (SOD), the first defense line against ROS like superoxide radicals ($O_2^{-}\bullet$) was activated in response to application of nanoparticles that converts the $O_2^{-}\bullet$ into hydrogen peroxide and oxygen ($2O_2^{-\bullet}+2H^{+}\rightarrow H_2O_2+O_2$) [54, 55]. Hydrogen peroxide (H_2O_2) is an important non-enzymatic antioxidant which acts as a signaling molecule as well as ROS. It is lethal to the pathogen at the optimum level but, when it exceeds, induces oxidative stress to the plants. Considering this oxidative stress, catalase activity (CAT) was triggered which converts the hydrogen peroxide from the dismutation ROS into water and oxygen ($2H_2O_2\rightarrow 2H_2O+O_2$). It is capable of detoxifying millions of H_2O_2 molecules in seconds [56]. After detoxification of ROS, peroxidases (POD) reinforced the cell walls of the wheat plants acting as a physical barrier against pathogen's penetration into host plant. These results are in line with the study of [57], who reported the increase of antioxidant defense system of wheat plants after the application of nanoparticles. Phenolic compounds such as TPC are rich in hydroxyl groups that provide hydrogen atoms to counteract ROS like superoxide radicals and hydrogen peroxide efficiently and diminish oxidative stress in plants through reinforcing the cell walls, protecting lipid membranes, and induction of SAR [58, 59, 60]. Total soluble sugars (TSS) like glucose, sucrose, and fructose are essential sources of energy that are produced by metabolic processes such as citric acid cycle and glycolysis and are essential for the upregulation of enzymatic antioxidant defense system. These enzymes need ATP under stress where its demand is heightened for its effective activity [61]. The outcomes of the existent study aligned with the work of [62, 63], who demonstrated that nanoparticles

upregulated the activity of both enzymatic and non-enzymatic antioxidants of the plants. Additionally, [64, 65, 66, 67-68] concluded that SiNPs exhibited rise in antioxidant defense system and noted significant enhancement in the growth of wheat plant by stimulating the antioxidant defense system through the application of silicon nanoparticles.

Nanoparticles allow precise release of bioactive compounds that not only suppress the negative effects of the phytopathogens but also promote the plant growth by inducing resistance in infected plants [30]. Current research effort was also directed to investigate the impact of *Parthenium hysterophorus* mediated Cu-Si hybrid nanotools towards the growth traits of wheat plant. The results of the study revealed that hybrid (CuNPs + SiNPs) nanoparticles expressed the maximum root length (cm), shoot length (cm), plant height (cm), spike length (cm), No. of grains per spike, spikelets per spike, 100 grains weight (g), chlorophyll contents (SPAD), root biomass (kg/ha) and stomatal conductance (m.mol $m^{-2}s^{-1}$), followed by CuNPs and SiNPs in comparison with control. Nanoparticles improved the chlorophyll contents of wheat by improving the uptake of the essential nutrients and then translocated to the leaves where synthesis of chlorophyll happened. Outcomes of this study are supported by the results of [69], who demonstrated that nanoparticles substantially enhanced the root length, shoot length and chlorophyll contents. Stomatal conductance was enhanced through the application of nanoparticles. Nanoparticles increased the water holding capacity of the wheat by strengthening the cell wall and increased root growth resulting in an increase in turgor pressure within guard cells (control stomatal opening) resulting in greater stomatal conductance. These findings are corroborated by the outcomes of [70], who revealed that stomatal conductance is elevated in the wheat plants treated with nanoparticles. The nanoparticles at lower concentrations significantly enhanced the overall plant height by improving the source-sink relationship, efficient nutrient and water uptake resulting in elevated photosynthetic activity. Similar results were observed by the findings of [71, 72], who demonstrated that nanoparticles

posed positive impacts on the plant height of wheat and rice. A surge in the shoot length, root length, no. of grains per spike was observed after the application of nanoparticles to wheat plants. This was due to the efficient nutrient uptake and stimulation of plant growth hormones such as gibberellins, cytokinin, and auxins responsible for the plant growth and these outcomes are endorsed by the results of [73, 74]. At an optimum level, nanoparticles improved the agronomic parameters of wheat like number of grains per spike, root biomass, grains weight, spike length. These results are in line with the work of [75], who expressed that the application of nanoparticles enhanced the agronomic parameters of wheat. Moreover, existent results are aligned with outcomes of [76,, 68, 57], who reported that nanoparticles significantly enhanced the agronomic attributes of wheat.

Conclusion

Under laboratory conditions, the combination of CuNPs and SiNPs showed maximal inhibition zone against *Xanthomonas translucens* pv. *undulosa*. While, under field and greenhouse conditions, hybrid nanoparticles formulation demonstrated the lowest disease incidence compared to the individual applications of copper and silicon nanoparticles. Similarly, enzymatic and non-enzymatic defense system and yield-related attributes of wheat plant. Therefore, it is concluded that the hybrid form of copper and silicon nanoparticles is highly recommended to manage bacterial leaf streak of wheat and enhance antioxidant defense system and yield-related attributes of wheat plant effectively. Future research should focus on optimizing the hybrid nanoparticles, exploring the action mechanisms, assess impacts, and expand their application to crops and agroclimatic zones.

Acknowledgment

We authors extend our gratitude to the Molecular Bacteriology and Plant Pathogen Interaction Laboratory, Department of Plant Pathology UAF, Pakistan for providing the laboratory facilities necessary for conducting research.

References

1. GOP. Economic advisor's wing, Finance Division, Government of Pakistan. Pakistan Economic Survey 2023-2024. 2024;2: 24. Available from: https://finance.gov.pk/survey_2024.html
2. Awulachew MT. The role of wheat in human nutrition and its medicinal value. Glob Acad J Med Sci. 2020;2(6): 50-54. doi: https://doi.org/10.36348/gajms.2020.v02i06.001
3. Iqbal MJ, Shams N, Fatima K. Nutritional quality of wheat. In: Ansari M, editor. Wheat-recent advances. IntechOpen, London, UK; 2022 Aug 23. pp. 1-19. Available from: https://www.intechopen.com/chapters/81564
4. Kanojia V, Kushwaha NL, Reshi M, Rouf A, Muzaffar H. Products and byproducts of wheat milling process. Int J Chem Stud. 2018;6(4): 990-993.
5. Hussain D, Asrar M, Khalid B, Hafeez F, Saleem M, Akhter M, et al. Insect pests of economic importance attacking wheat crop (*Triticum aestivum* L.) in Punjab, Pakistan. Int J Trop Insect Sci. 2022 Feb;42(1): 9-20. doi: https://doi.org/10.1007/s42690-021-00574-9
6. Niri MD, Tarighi S, Taheri P. Defense activation in wheat against *Xanthomonas translucens* via application of biological and chemical inducers. J Plant Pathol. 2023 May;105(2): 493-505. doi: https://doi.org/10.1007/s42161-023-01324-1
7. Osdaghi E, Taghavi SM, Aliabadi AA, Khojasteh M, Abachi H, Moallem M, et al. Detection and diagnosis of bacterial leaf streak on small grain cereals: From laboratory to field. Phytopathol. 2023 Nov 27;113(11): 2024-2036. doi: https://doi.org/10.1094/PHYTO-09-

22-0343-SA

8. Kaur N. Genotypic characterization and fungicide resistance monitoring for Virginia populations of *Parastagonospora nodorum* in wheat. Doctoral dissertation, Virginia Polytechnic Institute and State University. 2021. Available from: http://hdl.handle.net/10919/112959

9. Hangamaisho A, Bleakley BH, Ali S, Yabwalo D, Byamukama E. Aggressiveness of *Xanthomonas translucens* pv. *undulosa* isolates and differential reaction among spring wheat varieties under a controlled environment. Plant Health Prog. 2024 Feb 21;25(1): 33-36. doi: https://doi.org/10.1094/PHP-03-23-0020-RS

10. Ledman KE, Curland RD, Ishimaru CA, Dill-Macky R. *Xanthomonas translucens* pv. *undulosa* identified on common weedy grasses in naturally infected wheat fields in Minnesota. Phytopathol. 2021 Jul 30;111(7): 1114-1121. doi: https://doi.org/10.1094/PHYTO-08-20-0337-R

11. Shah SM, Haq F, Ma W, Xu X, Wang S, Xu Z, et al. $Tal_{1NXtc01}$ in *Xanthomonas translucens* pv. *cerealis* contributes to virulence in bacterial leaf streak of wheat. Front Microbiol. 2019 Sep 4;10: 2040. doi: https://doi.org/10.3389/fmicb.2019.02040

12. Mehmood A, Fahad A, Amin A, Ullah S, Fayyaz M, Fateh FS. A comprehensive review of significant bacterial diseases affecting wheat. Pak J Phytopathol. 2023 Jun 26;35(1): 191-199. doi: https://doi.org/10.33866/phytopathol.035.01.0868

13. Atiq M, Mazhar HM, Rajput NA, Ahmad U, Hameed A, Lodhi A, et al. Green synthesis of silver and copper nanoparticles from leaves of *Eucalyptus globulus* and assessment of its antibacterial potential towards *Xanthomonas citri* pv. *citri* causing citrus canker. Appl Ecol Environ Res. 2022 Jan 1;20(3): 2205-2213. doi: http://dx.doi.org/10.15666/aeer/2003_22052213

14. Hashimi MH, Hashimi R, Ryan Q. Toxic effects of pesticides on humans, plants, animals,

pollinators and beneficial organisms. Asian Plant Res J. 2020 Aug 5;5(4): 37-47. doi: https://doi.org/10.9734/aprj/2020/v5i430114

15. Karamchandani BM, Dalvi SG, Bagayatkar M, Banat IM, Satpute SK. Prospective applications of chitosan and chitosan-based nanoparticles formulations in sustainable agricultural practices. Biocat Agri Biotech. 2024 Jun;58: 103210. doi: https://doi.org/10.1016/j.bcab.2024.103210

16. Haidri I, Shahid M, Hussain S, Shahzad T, Mahmood F, Hassan MU, et al. Efficacy of biogenic zinc oxide nanoparticles in treating wastewater for sustainable wheat cultivation. Plants. 2023 Aug 25;12(17): 3058. doi: https://doi.org/10.3390/plants12173058

17. Shafqat U, Maqsood A, Ishfaq A, Mustafa S, Rasheed Y, Mahmood F, et al. Green nanotechnology for plant bacterial diseases management in cereal crops: a review on metal-based nanoparticles. Notulae Bot Horti Agrobotan Cluj-Napoca. 2023 Sep 27;51(3): 13333. doi: https://doi.org/10.15835/nbha51313333

18. Hernández-Díaz JA, Garza-García JJ, Zamudio-Ojeda A, León-Morales JM, López-Velázquez JC, García-Morales S. Plant-mediated synthesis of nanoparticles and their antimicrobial activity against phytopathogens. J Sci Food Agri. 2021 Mar 15;101(4): 1270-1287. doi: https://doi.org/10.1002/jsfa.10767

19. Khan I, Saeed K, Khan I. Nanoparticles: Properties, applications and toxicities. Arab J Chem. 2019 Nov 1;12(7): 908-931. doi: https://doi.org/10.1016/j.arabjc.2017.05.011

20. Ameta SK, Ameta SC. Eco-friendly approaches of using weeds for sustainable plant growth and production. In: Husen A, editor. Plant performance under environmental stress. Springer, Singapore; 2021. pp. 559-592. doi: https://doi.org/10.1007/978-3-030-78521-5

21. Javaid A, Munir R, Khan IH, Shoaib A. Control of the chickpea blight, *Ascochyta rabiei*, with the weed plant, *Withania somnifera*. Egypt J Biol Pest Cont. 2020 Dec;30: 114. doi: https://doi.org/10.1186/s41938-020-00315-z

22. Kashyap P, Shirkot P, Das R, Pandey H, Singh D. Biosynthesis and characterization of copper nanoparticles from *Stenotrophomonas maltophilia* and its effect on plant pathogens and pesticide degradation. J Agri Food Res. 2023 Sep 1;13: 100654. doi: https://doi.org/10.1016/j.jafr.2023.100654

23. Varympopi A, Dimopoulou A, Papafotis D, Avramidis P, Sarris I, Karamanidou T, et al. Antibacterial activity of copper nanoparticles against *Xanthomonas campestris* pv. *vesicatoria* in tomato plants. Int J Mol Sci. 2022 Apr 7;23(8): 4080. doi: https://doi.org/10.3390/ijms23084080

24. Luong HT, Nguyen CX, Lam TT, Nguyen TH, Dang QL, Lee JH, et al. Antibacterial effect of copper nanoparticles produced in a Shewanella-supported non-external circuit bioelectrical system on bacterial plant pathogens. RSC Adv. 2022;12(7): 4428-4436. doi: https://doi.org/10.1039/D1RA08187J

25. Siddiqui ZA, Hashmi A, Khan MR, Parveen A. Management of bacteria *Pectobacterium carotovorum*, *Xanthomonas campestris* pv. *carotae*, and fungi *Rhizoctonia solani*, *Fusarium solani* and *Alternaria dauci* with silicon dioxide nanoparticles on carrot. Int J Veg Sci. 2020 Nov 1;26(6): 547-557. doi: https://doi.org/10.1080/19315260.2019.1675843

26. Yuvaraj M, Priya RS, Jagathjothi N, Saranya M, Suganthi N, Sharmila R, et al. Silicon nanoparticles (SiNPs): Challenges and perspectives for sustainable agriculture. Physiol Mol Plant Pathol. 2023 Oct 5: 102161. doi: https://doi.org/10.1016/j.pmpp.2023.102161

27. Ali S, Ulhassan Z, Shahbaz H, Kaleem Z, Yousaf MA, Ali S, et al. Magnesium oxide nanoparticles as novel sustainable approach in enhancing crop tolerance to abiotic and biotic stresses. Environ Sci: Nano. 2024 Jul 5;11(8): 3250-3267. doi: https://doi.org/10.1039/D4EN00417E

28. Huang Q, Ayyaz A, Farooq MA, Zhang K, Chen W, Hannan F, et al. Silicon dioxide nanoparticles enhance plant growth, photosynthetic performance, and antioxidants defence

machinery through suppressing chromium uptake in *Brassica napus* L. Environ Poll. 2024 Feb 1;342: 123013. doi: https://doi.org/10.1016/j.envpol.2023.123013

29. Ijaz M, Khan F, Ahmed T, Noman M, Zulfiqar F, Rizwan M, et al. Nanobiotechnology to advance stress resilience in plants: Current opportunities and challenges. Mat Today Bio. 2023 Aug 6;22: 100759. doi: https://doi.org/10.1016/j.mtbio.2023.100759

30. Kralova K, Jampilek J. Applications of nanomaterials in plant disease management and protection. In: Ingle AP, editor. Nanotechnology in agriculture and agroecosystems. Micro Nano Tech. 2023 Jan 1: 239-296. doi: https://doi.org/10.1016/B978-0-323-99446-0.00013-1

31. Mary RS, Mahesh MK, Pruthvi ML. Biosynthesis of copper nanoparticles using *Parthenium hysterophorus* leaf extract and screening its antimicrobial activity. Asian J Pharm Clinic Res. 2020;13(1): 93-99. doi: http://dx.doi.org/10.22159/ajpcr.2020.v13i1.36076

32. Seem RC. Disease incidence and severity relationships. Ann Rev Phytopathol. 1984 Sep;22(1): 133-150.

33. Giannopolitis CN, Ries SK. Superoxide dismutases: I. Occurrence in higher plants. Plant Physiol. 1977 Feb 1;59(2): 309-314. doi: https://doi.org/10.1104/pp.59.2.309

34. Yemm EW, Willis A. The estimation of carbohydrates in plant extracts by anthrone. Biochem J. 1954 Jul;57(3): 508-514. doi: https://doi.org/10.1042%2Fbj0570508

35. Steel, R.G.D., J.H. Torrie and D.A. Dickey. Principles and Procedures of Statistics: A biometrical approach, 3rd Ed. McGraw-Hill., United States of America. 1997.

36. Hangamaisho A. Efficacy of nanomaterials and biorationals on the bacterial leaf streak management in wheat. M.Sc. Thesis, South Dakota State University; 2022. Available from: https://openprairie.sdstate.edu/etd2/416

37. Atiq M, Fatima T, Rajput NA, Usman M, Kachelo GA, Ahmad U, et al. *Moringa olefra*

plant based copper and silver nano particles and its antibacterial activity towards leaf spot of chilli caused by *Xanthomonas campestris* pv. *vesicatoria*. Int J Phytopath. 2023 Apr 30;12(1): 63-72. doi: https://doi.org/10.33687/phytopath.012.01.4306

38. Chauhan H, Patel M, Patel P, Tiwari S, Jinal HN, Amaresan N. Assessment of copper (Cu) nanoparticle for their biocontrol activity against *Xanthomonas oryzae* pv. *oryzae*, growth promotion, and physiology of rice (*Oryza sativa* L.) plants. Lett Appl Microbiol. 2023 Jan;76(1): ovac066. doi: https://doi.org/10.1093/lambio/ovac066

39. Chowdhury AR, Kumar R, Mahanty A, Mukherjee K, Kumar S, Tribhuvan KU, et al. Inhibitory role of copper and silver nanocomposite on important bacterial and fungal pathogens in rice (*Oryza sativa*). Sci Rep. 2024 Jan 20;14(1):1779. doi: https://doi.org/10.1038/s41598-023-49918-0

40. Iliger KS, Sofi TA, Bhat NA, Ahanger FA, Sekhar JC, Elhendi AZ, et al. Copper nanoparticles: Green synthesis and managing fruit rot disease of chilli caused by *Colletotrichum capsici*. Saudi J Biol Sci. 2021 Feb 1;28(2): 1477-86. doi: https://doi.org/10.1016/j.sjbs.2020.12.003

41. Nazir A, Rehman SU, Abbas M, Iqbal DN, Ahsraf AR, Iqbal M. Green synthesis and characterization of copper nanoparticles using *Parthenium hysterophorus* extract: Antibacterial and antioxidant activities evaluation. Chem Int. 2022;8(2): 68-76. doi: https://doi.org/10.5281/zenodo.6849915

42. Abdelrhim AS, Mazrou YS, Nehela Y, Atallah OO, El-Ashmony RM, Dawood MF. Silicon dioxide nanoparticles induce innate immune responses and activate antioxidant machinery in wheat against *Rhizoctonia solani*. Plants. 2021 Dec 14;10(12): 2758. doi: https://doi.org/10.3390/plants10122758

43. Manzoor N, Ali L, Ahmed T, Rizwan M, Ali S, Shahid MS, et al. Silicon oxide nanoparticles alleviate chromium toxicity in wheat (*Triticum aestivum* L.). Environ Poll.

2022 Dec 15;315: 120391. doi: https://doi.org/10.1016/j.envpol.2022.120391

44. Vasiliev G, Kubo AL, Vija H, Kahru A, Bondar D, Karpichev Y, et al. Synergistic antibacterial effect of copper and silver nanoparticles and their mechanism of action. Sci Rep. 2023 Jun 6;13(1): 9202. doi: https://doi.org/10.1038/s41598-023-36460-2

45. Wasule DL, Shingote PR, Saxena S. Exploitation of functionalized green nanomaterials for plant disease management. Disco Nano. 2024 Jul 18;19(1): 118. doi: https://doi.org/10.1186/s11671-024-04063-z

46. Raffi M, Mehrwan S, Bhatti TM, Akhter JI, Hameed A, Yawar W, et al. Investigations into the antibacterial behavior of copper nanoparticles against *Escherichia coli*. Ann Microbiol. 2010 Mar;60: 75-80. doi: https://doi.org/10.1007/s13213-010-0015-6

47. Shende S, Ingle AP, Gade A, Rai M. Green synthesis of copper nanoparticles by *Citrus medica* Linn. (Idilimbu) juice and its antimicrobial activity. World J Microbiol Biotechnol. 2015 Jun;31: 865-873. Doi: https://doi.org/10.1007/s11274-015-1840-3

48. Mittal D, Kaur G, Singh P, Yadav K, Ali SA. Nanoparticle-based sustainable agriculture and food science: Recent advances and future outlook. Front Nanotech. 2020 Dec 4;2: 579954. doi: https://doi.org/10.3389/fnano.2020.579954

49. Goswami P, Mathur J, Srivastava N. Silica nanoparticles as novel sustainable approach for plant growth and crop protection. Heliyon. 2022 Jul 7;8(7): e09908. doi: https://doi.org/10.1016/j.heliyon.2022.e09908

50. Silveira PR, Nascimento KJ, Andrade CC, Bispo WM, Oliveira JR, Rodrigues FA. Physiological changes in tomato leaves arising from *Xanthomonas gardneri* infection. Physiol Mol Plant Pathol. 2015 Oct 1;92: 130-138. doi: https://doi.org/10.1016/j.pmpp.2015.10.001

51. Rizvi A, Chandrawal R, Khan MH, Ahmed B, Umar S, Khan MS. Microbiological control of *Xanthomonas* induced bacterial leaf streak disease of wheat via phytocompounds and

ros processing enzymes produced under biotic stress. J Plant Growth Reg. 2024 Feb;43(2): 601-623. doi: https://doi.org/10.1007/s00344-023-11119-4

52. Choudhary A, Kumar A, Kaur N. ROS and oxidative burst: Roots in plant development. Plant Divers. 2020 Feb 1;42(1): 33-43. doi: https://doi.org/10.1016/j.pld.2019.10.002

53. Hameed A, Atiq M, Sahi ST, Rajput NA, Ahmed Z, Alam MW, et al. Biochemical base of resistance in citrus against canker disease. Pak J Agri Sci. 2021 Jan 16;58(6): 1850-1858. doi: 10.21162/PAKJAS/21.11

54. Rasheed Z. Superoxide dismutase: Challenges, opportunities, and promises for clinical translation. Int J Health Sci. 2024 May;18(3): 1-3. doi: https://pubmed.ncbi.nlm.nih.gov/38721138

55. Zhao H, Zhang R, Yan X, Fan K. Superoxide dismutase nanozymes: an emerging star for anti-oxidation. J Mat Chem B. 2021;9(35): 6939-57. doi: https://doi.org/10.1039/D1TB00720C

56. Orlikowski L, Sas-Paszt L, Wojdyła A, Orlikowska T. The use of hydrogen peroxide and silver nanoparticles in horticulture. J Horti Res. 2023 Dec 29;31(2): 1-22. doi: https://doi.org/10.2478/johr-2023-0037

57. Thakur N, Rana S, Pathak A. Impact of chemically synthesized copper nanoparticles on growth and biochemical profiling of wheat (*Triticum aestivum* L.). Int J Biol, Phar Alli Sci. 2022 Jun 1;11: 2638-2651. doi: https://doi.org/10.31032/IJBPAS/2022/11.6.6127

58. Liu W, Cui X, Zhong Y, Ma R, Liu B, Xia Y. Phenolic metabolites as therapeutic in inflammation and neoplasms: Molecular pathways explaining their efficacy. Pharm Res. 2023 Jul 1;193: 106812. doi: https://doi.org/10.1016/j.phrs.2023.106812

59. Martínez-Rubio R, Centeno ML, García-Angulo P, Álvarez JM, Acebes JL, Encina A. The role of cell wall phenolics during the early remodelling of cellulose-deficient maize cells. Phytochem. 2020 Feb 1;170: 112219. doi:

https://doi.org/10.1016/j.phytochem.2019.112219
60. War AF, Nanda SA, Bashir I, Rehmaan S, Sheergojri IA, Rehman IU, et al. Plant phenolics role in bacterial disease stress management in plants. In: Lone R, Khan S, Al-Sadi AM, editors. Plant Phenolics in Biotic Stress Management. Springer, Singapore. 2024 Feb 28. pp. 217-241. doi: https://doi.org/10.1007/978-981-99-3334-1
61. Afzal S, Chaudhary N, Singh NK. Role of soluble sugars in metabolism and sensing under abiotic stress. In: Aftab T, Hakeem KR, editors. Plant growth regulators. Springer, Singapore; 2021. pp. 305-334. doi: https://doi.org/10.1007/978-3-030-61153-8
62. Abdelkader M, Geioushy RA, Fouad OA, Khaled AG, Liudmila PV. Investigation the activities of photosynthetic pigments, antioxidant enzymes and inducing genotoxicity of cucumber seedling exposed to copper oxides nanoparticles stress. Sci Hortic. 2022 Nov 17;305: 111364. doi: https://doi.org/10.1016/j.scienta.2022.111364
63. Wang W, Ren Y, He J, Zhang L, Wang X, Cui Z. Impact of copper oxide nanoparticles on the germination, seedling growth, and physiological responses in *Brassica pekinensis* L. Environ Sci Poll Res. 2020 Sep;27: 31505-31515. doi: https://doi.org/10.1007/s11356-020-09338-3
64. Huang G, Zuverza-Mena N, White JC, Hu H, Xing B, Dhankher OP. Simultaneous exposure of wheat (*Triticum aestivum* L.) to CuO and S nanoparticles alleviates toxicity by reducing Cu accumulation and modulating antioxidant response. Sci Total Environ. 2022 Sep 15;839: 156285. doi: https://doi.org/10.1016/j.scitotenv.2022.156285
65. Khan ZS, Rizwan M, Hafeez M, Ali S, Adrees M, Qayyum MF, et al. Effects of silicon nanoparticles on growth and physiology of wheat in cadmium contaminated soil under different soil moisture levels. Environ Sci Poll Res. 2020 Feb;27: 4958-4968. doi: https://doi.org/10.1007/s11356-019-06673-y
66. Rahman SU, Xuebin Q, Yasin G, Cheng H, Mehmood F, Zain M, et al. Role of silicon on

root morphological characters of wheat (*Triticum aestivum* L.) plants grown under Cd-contaminated nutrient solution. Acta Physiol Plan. 2021 Mar 16; 43:60. doi: https://doi.org/10.1007/s11738-021-03228-y

67. Raza MA, Zulfiqar B, Iqbal R, Muzamil MN, Aslam MU, Muhammad F, et al. Morpho-physiological and biochemical response of wheat to various treatments of silicon nano-particles under drought stress conditions. Sci Rep. 2023 Feb 15;13(1): 2700. doi: https://doi.org/10.1038/s41598-023-29784-6

68. Riaz M, Zhao S, Kamran M, Rehman NU, Mora-Poblete F, Maldonado C, et al. Effect of nano-silicon on the regulation of ascorbate-glutathione contents, antioxidant defense system and growth of copper stressed wheat (*Triticum aestivum* L.) seedlings. Front Plant Sci. 2022 Oct 13;13: 986991. doi: https://doi.org/10.3389/fpls.2022.986991

69. Essa HL, Abdelfattah MS, Marzouk AS, Shedeed Z, Guirguis HA, El-Sayed MM. Biogenic copper nanoparticles from *Avicennia marina* leaves: Impact on seed germination, detoxification enzymes, chlorophyll content and uptake by wheat seedlings. PLoS One. 2021 Apr 15;16(4): e0249764. doi: https://doi.org/10.1371/journal.pone.0249764

70. Ahmed F, Javed B, Razzaq A, Mashwani ZU. Applications of copper and silver nanoparticles on wheat plants to induce drought tolerance and increase yield. IET Nanobiotech. 2021 Feb;15(1): 68-78. doi: https://doi.org/10.1049/nbt2.12002

71. Ahmed T, Noman M, Manzoor N, Shahid M, Abdullah M, Ali L, et al. Nanoparticle-based amelioration of drought stress and cadmium toxicity in rice via triggering the stress responsive genetic mechanisms and nutrient acquisition. Ecotoxicol Environ Saf. 2021 Feb 1;209: 111829.doi: https://doi.org/10.1016/j.ecoenv.2020.111829

72. Raza MA, Aslam MU, Valipour M, Iqbal R, Haider I, Mustafa AE, et al. Seed priming with selenium improves growth and yield of quinoa plants suffering drought. Sci Rep. 2024 Jan 9;14(1): 886. doi: https://doi.org/10.1038/s41598-024-51371-6

73. Yasmeen F, Raja NI, Razzaq A, Komatsu S. Proteomic and physiological analyses of wheat seeds exposed to copper and iron nanoparticles. Biochim Biophys Acta (BBA)-Prot Proteo. 2017 Jan 1;1865(1): 28-42. doi: https://doi.org/10.1016/j.bbapap.2016.10.001

74. Nazir MA, Hasan M, Mustafa G, Tariq T, Ahmed MM, Golzari Dehno R, et al. Zinc oxide nano-fertilizer differentially effect on morphological and physiological identity of redox-enzymes and biochemical attributes in wheat (*Triticum aestivum* L.). Sci Rep. 2024 Jun 7;14(1): 13091. doi: https://doi.org/10.1038/s41598-024-63987-9

75. Rashid MI, Shah GA, Sadiq M, Amin NU, Ali AM, Ondrasek G, et al. Nanobiochar and copper oxide nanoparticles mixture synergistically increases soil nutrient availability and improves wheat production. Plants. 2023 Mar 14;12(6): 1312. doi: https://doi.org/10.3390/plants12061312

76. Kausar H, Mehmood A, Khan RT, Ahmad KS, Hussain S, Nawaz F, et al. Green synthesis and characterization of copper nanoparticles for investigating their effect on germination and growth of wheat. PLoS One. 2022 Jun 21;17(6): e0269987. doi: https://doi.org/10.1371/journal.pone.0269987

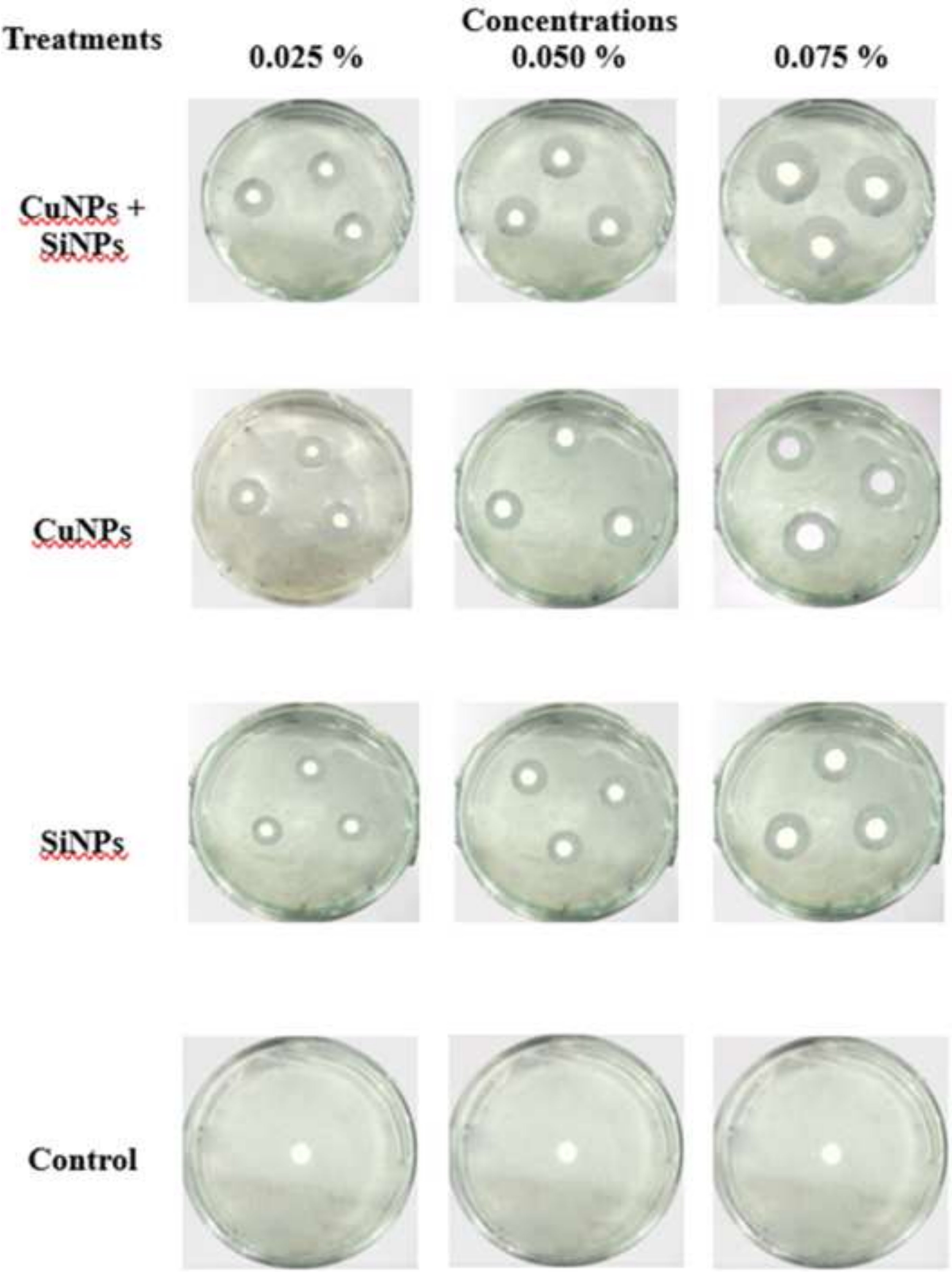
Treatments
Concentrations
0.025 %
0.050 %
0.075 %
CuNPs + SiNPs
CuNPs
SiNPs
Control

Figure2

Click here to access/download;Figure;Fig 2.tif

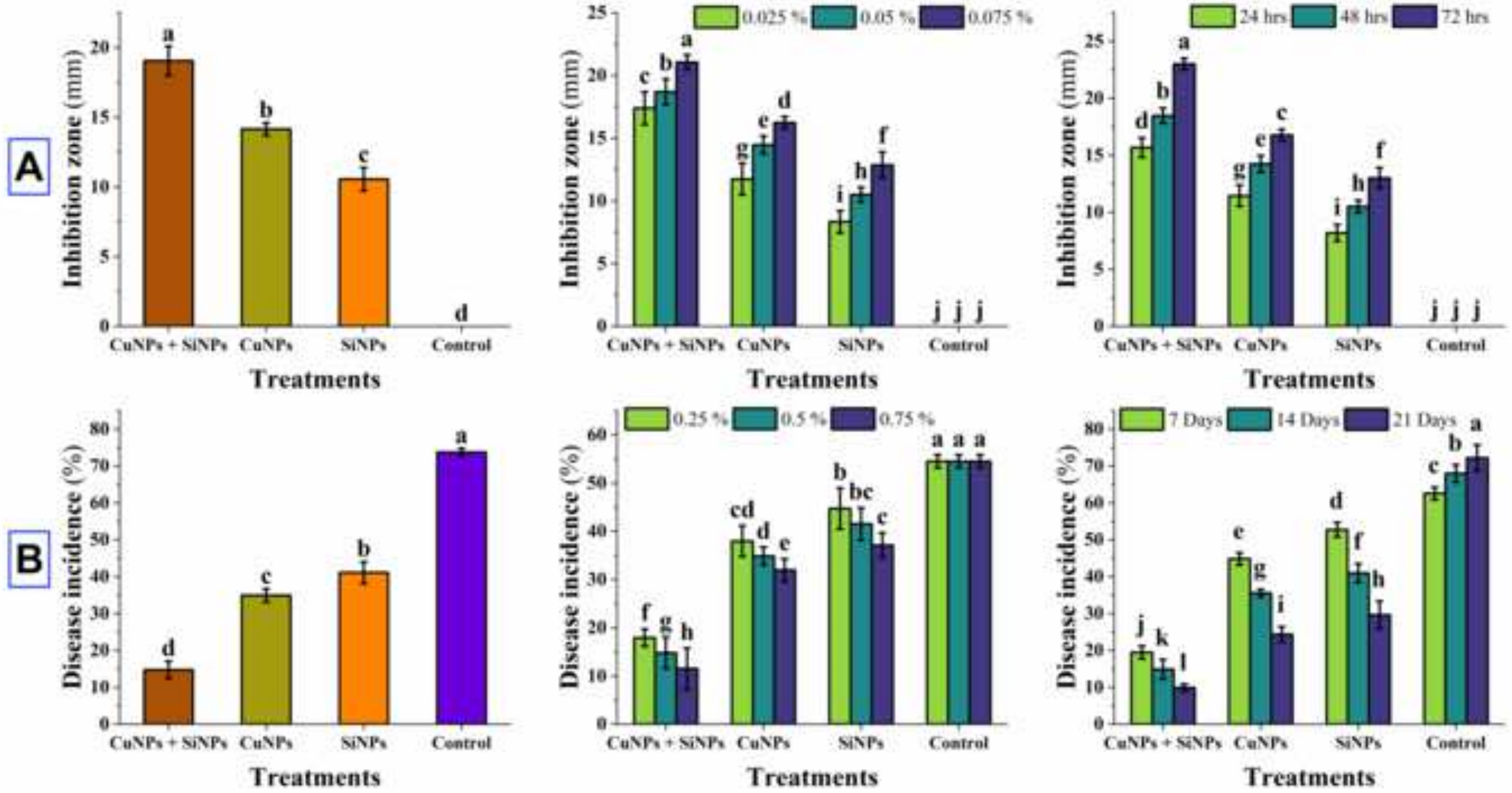

Figure 3

Click here to access/download;Figure;Fig 3.tif

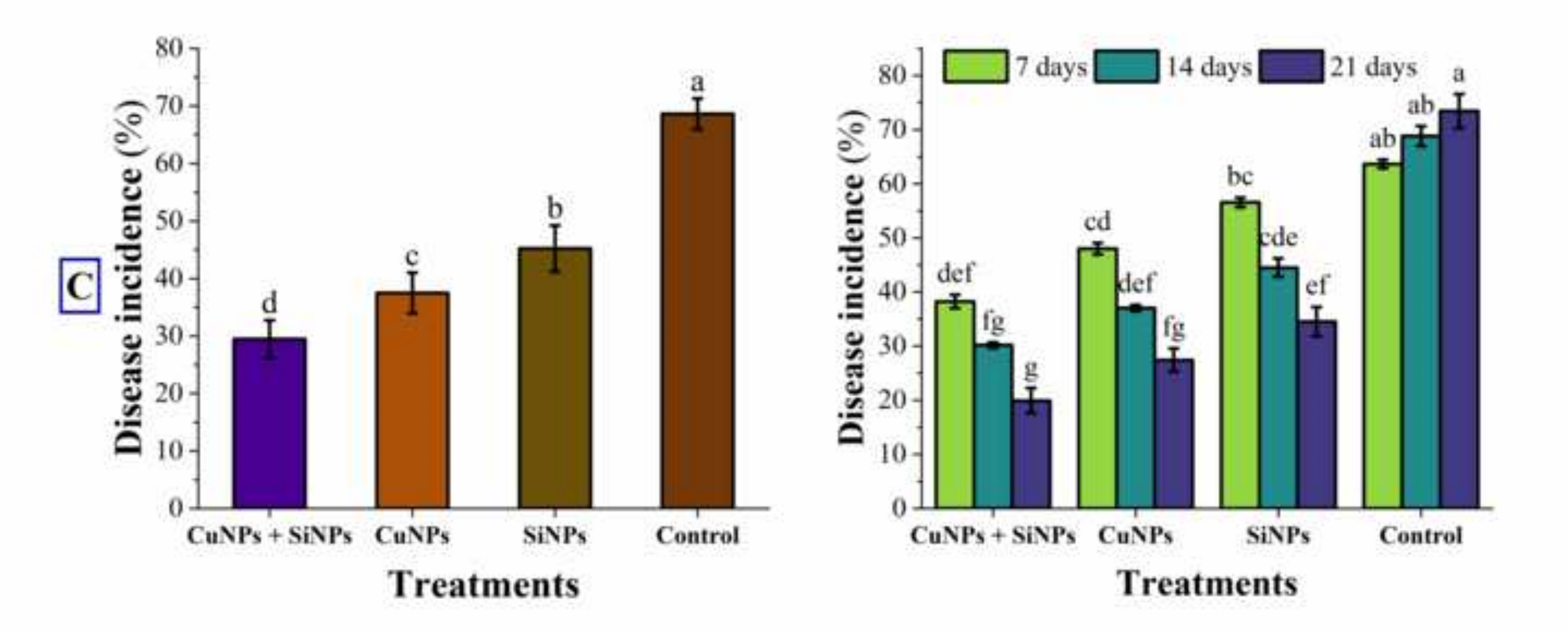

Figure 4

Click here to access/download;Figure;Fig 4.tif

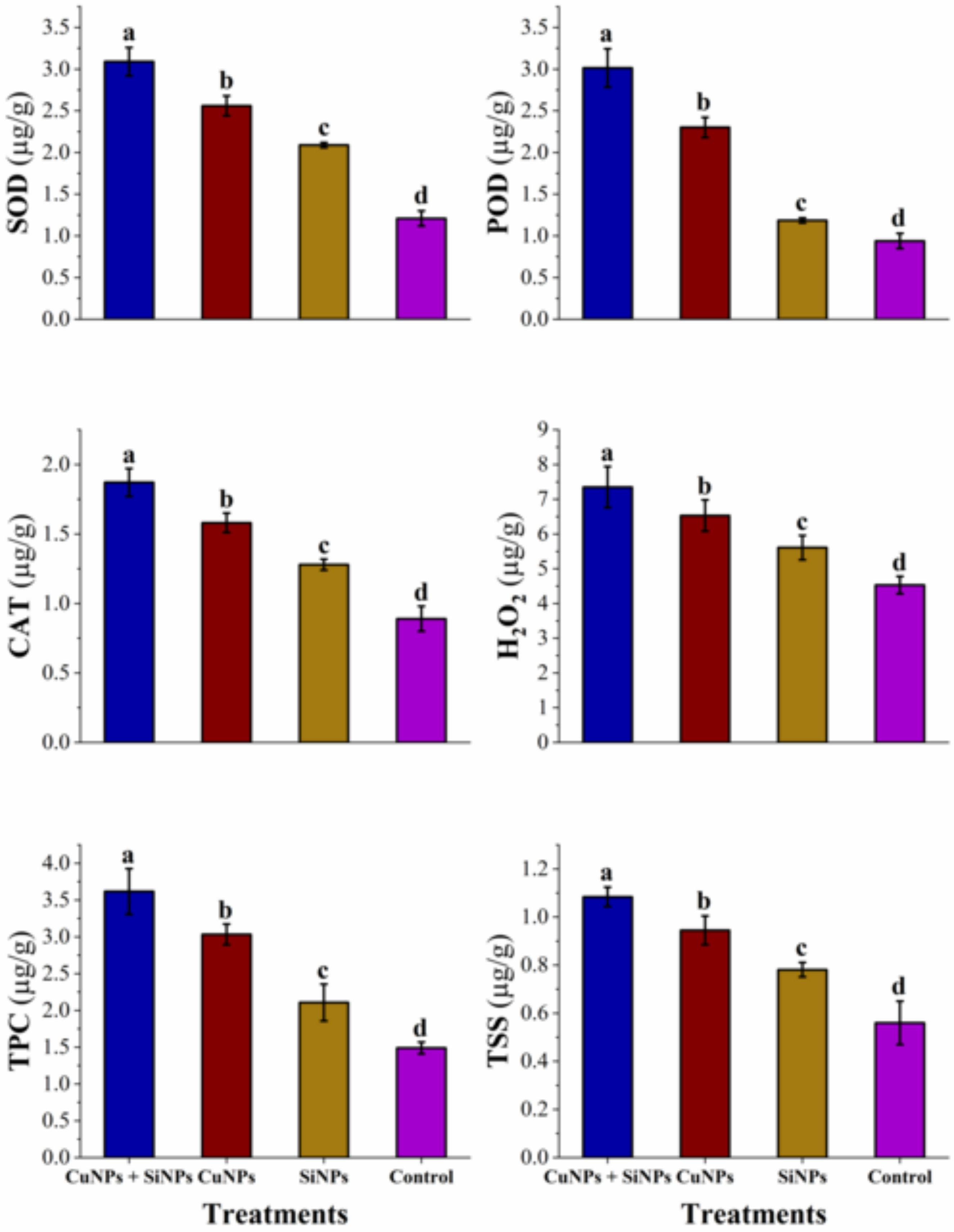

Figure 5B

Click here to access/download;Figure;Fig 5A.tif

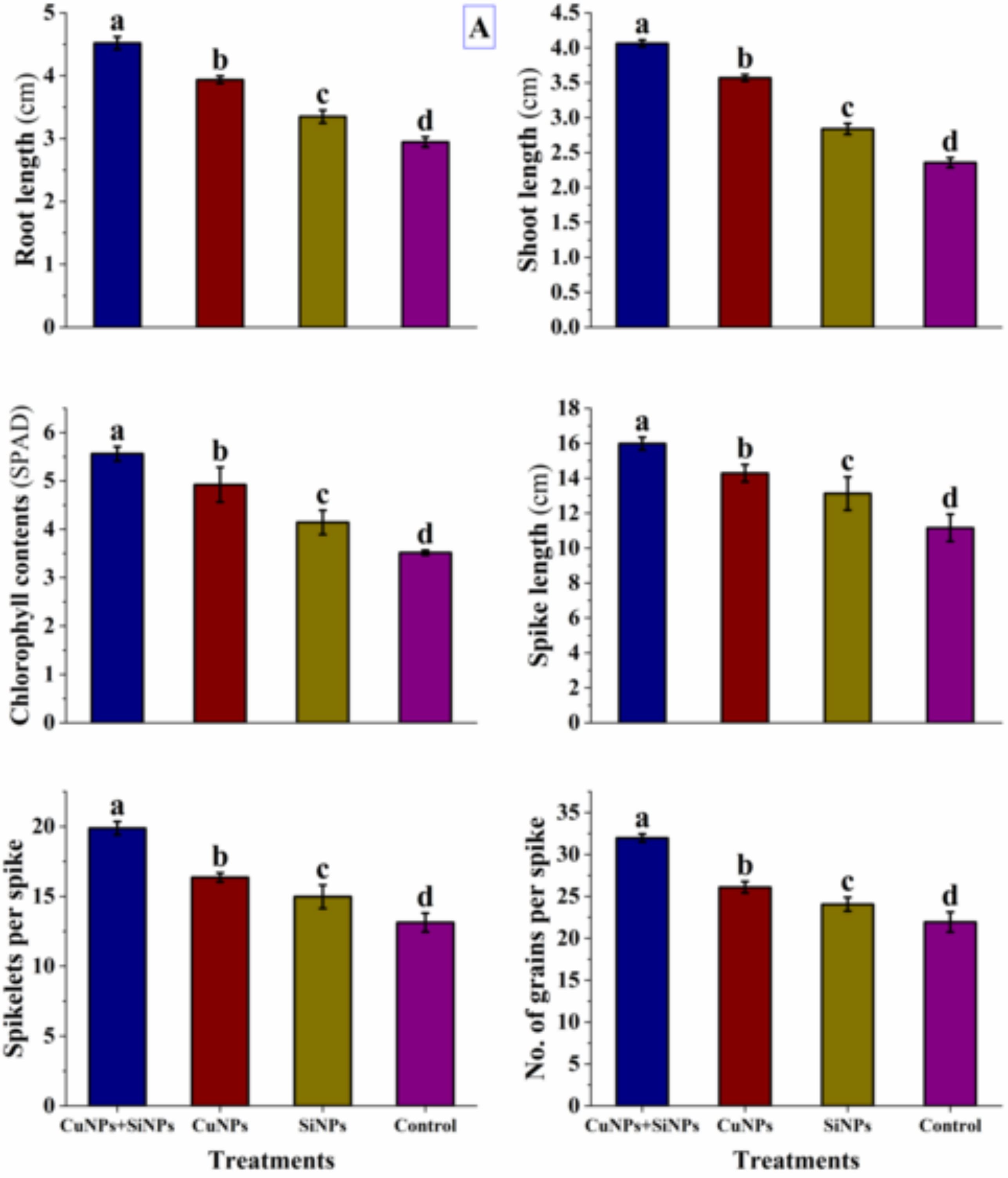

Figure 5B

Click here to access/download;Figure;Fig 5B.tif

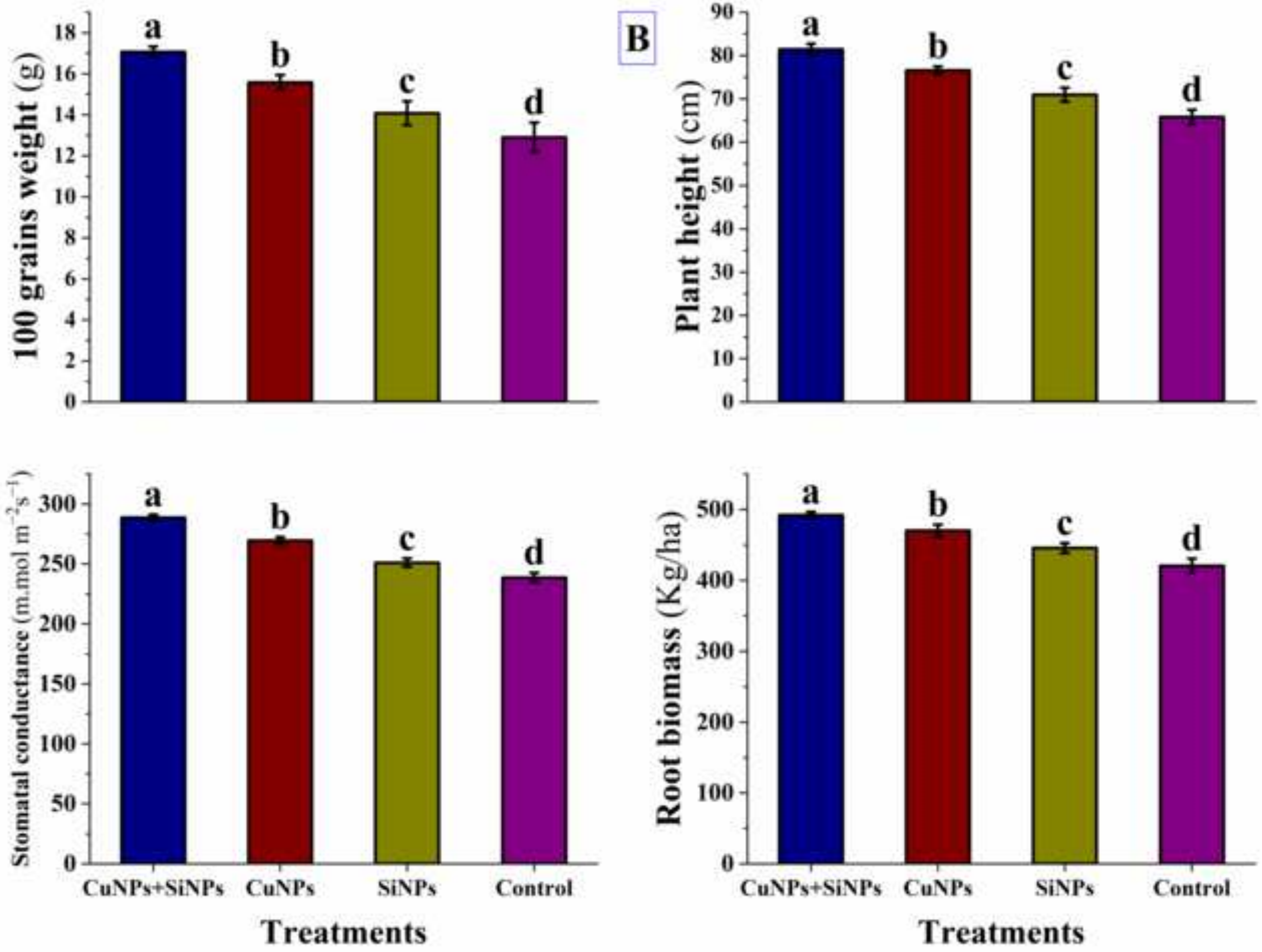

Figure 6

Click here to access/download;Figure;Fig 6.tif

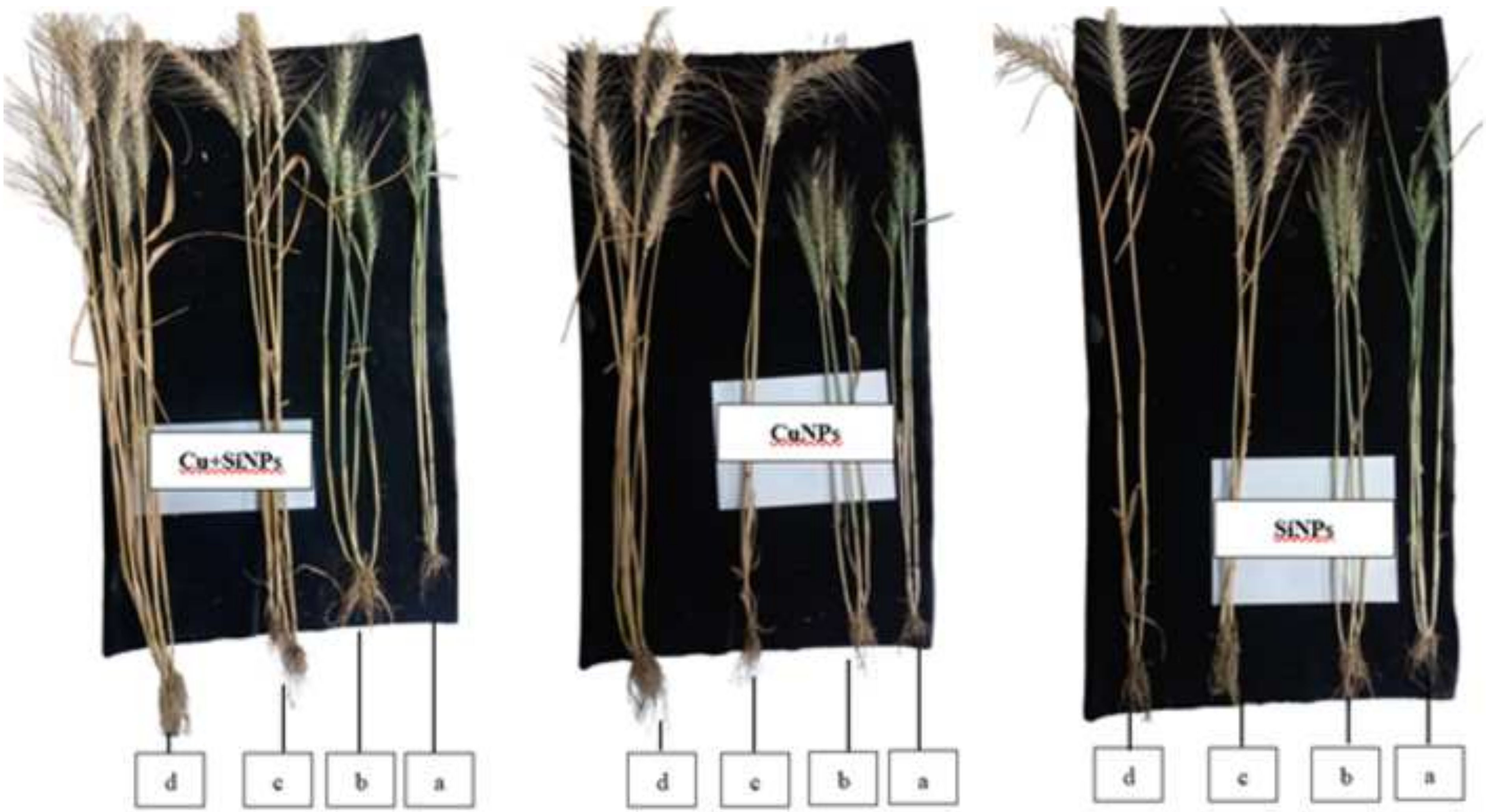

Click here to access/download
Supporting Information
S1. Evaluation of Nanoparticles (1).xlsx

Click here to access/download
Supporting Information
S2. Biochemical data (1).xlsx

Click here to access/download
Supporting Information
S3. Agronomic attributes data (1).xlsx

Important declarations

Please remove this info from manuscript text if it is also present there.

Associated Data

Data supplied by the author:
The raw measurements are available in the supplementary file.

Required Statements

Competing Interest statement:
The authors declare that they have no competing interests.

Funding statement:
Supported by Researchers Supporting Project Number (RSP2023R410), King Saud University, Riyadh, Saudi Arabia.

Epidemiology and management of Fusarium wilt of *Eucalyptus camaldulensis* through systemic acquired resistance

Irfan Ahmad Corresp., Equal first author, 1 , **Komal Mazhar** Equal first author, 1 , **Muhammad Atiq** 2 , **Amna Kahtan Khalaf** 3 , **Muhammad Haroon U Rashid** Corresp., 1 , **Muhammad Asif** 1 , **Salman Ahmed** 4 , **Zoha Adil** 2 , **Amna Fayyaz** 5 , **Mohammad Khalid Al- Sadoon** 6 , **Hammad Al-Otaibi** 6

1 Department of Forestry and Range Management, University of Agriculture Faisalabad, Faisalabad, Punjab, Pakistan
2 Department of Plant Pathology, University of Agriculture Faisalabad, Faisalabad, Punjab, Pakistan
3 College of Dentistry, Al-Bayan University, Baghdad, Iraq
4 Department of Plant Pathology, University of Sargodha, Sargodha, Punjab, Pakistan
5 Department of Plant Pathology, University of California, Davis, California, California, United States
6 Department of Zoology, King Saud University, Riyadh, Saudi Arabia

Corresponding Authors: Irfan Ahmad, Muhammad Haroon U Rashid
Email address: irfanahmad@uaf.edu.pk, haroon.r@uaf.edu.pk

Eucalyptus camaldulensis is a multifunctional tree and is globally used for the reclamation of problematic lands. *Eucalyptus camaldulensis* is prone to attack by a number of pathogens, but the most important threat is the Fusarium wilt (*Fusarium oxysporum*). Keeping in view the importance of *E. camaldulensis* and to manage this disease, five plant activators, i.e., Salicylic acid ($C_7H_6O_3$), Benzoic acid ($C_7H_6O_{23}$), Citric acid ($C_6H_8O_7$), Dipotassium phosphate (K_2HPO_4), Monopotassium phosphate (KH_2PO_4) and nutritional mixture namely Compound (NPK) and nutriotop (Fe, Zn, Cu, B, Mn) were evaluated in the Fusarium infested field under RCBD in the Research Area, Department of Forestry and Range Management, University of Agriculture, Faisalabad (UAF). Among plant activators, salicylic acid and a combination of compound + nutriotop exhibited the lowest disease incidence and enhanced fresh and dry weight of leaves compared to other treatments and control. Results of the environmental study indicated maximum disease incidence between 35 34˚C (max. T), 6-25˚C (mini. T), 70-80% relative humidity and 1.5-2.5 km/h wind speed while pan evaporation expressed week correlation with disease development. It was concluded that Fusarium wilt of *Eucalyptus camaldulensis* could be managed through activation of the basal defense system of the host plant with provision of salicylic acid and balanced nutrition by considering environmental factors. Recent exploration is expected to be helpful for future research efforts on epidemiology and ecologically sound intervention of Fusarium wilt of *Eucalyptus camaldulensis*.

Commented [A1]: C7H6O2 CORRECTED

Epidemiology and management of Fusarium wilt of *Eucalyptus camaldulensis* through systemic acquired resistance

Irfan Ahmad[1†*], Komal Mazhar[1†], Muhammad Atiq[2], Amna Kahtan Khalaf[3], Muhammad Haroon U. Rashid[1*], Muhammad Asif[1], Salman Ahmed[4], Zoha Adil[2], Amna Fayyaz[5], Mohammad Khalid Al-Sadoon[6], Hammad Al-Otaibi[6]

[1] Department of Forestry and Range Management, University of Agriculture, Faisalabad, 38000, Pakistan.
[2] Department of Plant Pathology, University of Agriculture, Faisalabad, 38000, Pakistan.
[3] College of Dentistry, Al-Bayan University, Baghdad 10059, Iraq.
[4] Department of Plant Pathology, University of Sargodha, 40100, Pakistan.
[5] Department of Plant Pathology, University of California Davis, USA.
[6] Department of Zoology, College of Science, King Saud University, PO Box 2455, Riyadh,11451, Saudi Arabia.

† These authors contributed equally to this work.

*Corresponding Author-1:
Irfan Ahmad[1]
Department of Forestry and Range Management, University of Agriculture, Faisalabad, 38000, Pakistan
Email address: irfanahmad@uaf.edu.pk

*Corresponding Author-2:
Muhammad Haroon U. Rashid[1]
Department of Forestry and Range Management, University of Agriculture, Faisalabad, 38000, Pakistan
Email address: haroon.r@uaf.edu.pk

Abstract

Eucalyptus camaldulensis is a multifunctional tree and is globally used for the reclamation of problematic lands. *Eucalyptus camaldulensis* is prone to attack by a number of pathogens, but the most important threat is the Fusarium wilt (*Fusarium oxysporum*). Keeping in view the importance of *E. camaldulensis* and to manage this disease, five plant activators, i.e., Salicylic acid ($C_7H_6O_3$), Benzoic acid ($C_7H_6O_3$), Citric acid ($C_6H_8O_7$), Dipotassium phosphate (K_2HPO_4), Monopotassium phosphate (KH_2PO_4) and nutritional mixture namely Compound (NPK) and nutriotop (Fe, Zn, Cu, B, Mn) were evaluated in the Fusarium infested field under RCBD in the Research Area, Department of Forestry and Range Management, University of Agriculture, Faisalabad (UAF). Among plant activators, salicylic acid and a combination of compound + nutriotop exhibited the lowest disease incidence and enhanced fresh and dry weight of leaves compared to other treatments and control. Results of the environmental study indicated maximum disease incidence between 35 – 40 °C (max. T), 6-25°C (mini. T), 70-80% relative humidity and 1.5-2.5 km/h wind speed while pan evaporation expressed week correlation with disease development. It was concluded that Fusarium wilt of *Eucalyptus camaldulensis* could be managed through activation of the basal defense system of the host plant with provision of salicylic acid and balanced nutrition by considering environmental factors. Recent exploration is expected to be helpful for future research efforts on epidemiology and ecologically sound intervention of Fusarium wilt of *Eucalyptus camaldulensis*.

Keywords: *Eucalyptus camaldulensis*, SAR, epidemiology, *Fusarium oxysporum*, Plant nutrition

Commented [A2]: C7H6O2 CORRECTED

Commented [A3]: Weak (correction of spelling weak has been done)

Introduction

Eucalyptus camaldulensis Dehnh. (Sufaida) belongs to the family Myrtaceae, found in tropical and subtropical regions. It is a fast-growing species, native to Australia that can grow under a wide range of climate and edaphic conditions and also known as river red gum [1-3]. It is native to Australia and is known as river red gum. Pakistan, India, Algeria, East Africa, Sudan, Malaysia, Philippines, and Ethiopia are Eucalyptus growing countries of the world, and according to an estimate, its plantation has reached more than 20 million hectares [4]. Globally, Eucalyptus is used in agroforestry for reclamation of problematic soils [5]. Commonly grown regions of Eucalyptus are Sindh and Punjab in Pakistan. This multipurpose tree is a rich source of byproducts like honey, medicinal extracts and perfumes. Essential oils extracted from Eucalyptus leaves have shown a remarkable antiseptic activity against a wide range of infectious bacteria, viruses, and fungi [6]. Eucalyptus is prone to be attacked by a number of fungal, bacterial, and viral pathogens in different regions, but *Fusarium oxysporum* is responsible for Fusarium wilt disease poses significant threat [7]. *F. oxysporum* is a soil-born pathogen that attacks roots and colonizes vascular tissue, causing wilts in Eucalyptus. Chlorosis, wilting, necrosis, immature leaf dropping, stunting, damping off, and browning of the vascular system are the characteristic symptoms of Fusarium wilt of Eucalyptus [7-8]. The first disease symptom can be observed on the plants in the form of pale green streaks on the base of the petiole. *F. oxysporum* is a soil-born pathogen that attacks roots and colonizes vascular tissue, causing wilts in Eucalyptus [8].

The pathogen population and their attack on the host plant are interlinked with the environment [9]. Thus, it is necessary to study the effect of all the environmental factors involved in the development of the pathogen's population dynamics and disease development [9-10]. It is of prime importance for the management of Fusarium wilt of eucalyptus, as climate has a major role in the distribution of Fusarium species which are thermophilic in nature and are significantly correlated with soil and environmental factors [10]. High temperature and moisture are favorable for the disease occurrence. Reduction in soil moisture is a significant strategy for managing this disease [11]. Land et al. [12] and Larkin and Fravel [13] reported that 17-24°C air and 15-25°C soil temperature played a crucial role in developing Fusarium wilt disease. Therefore, sudden fluctuations in environmental conditions can increase wilt disease incidence. In various regions researchers are now compelled to investigate the role of biotic and abiotic factors facilitating disease emergence.

In the light of the above-mentioned facts the study investigated how soil and ambient environmental conditions affect the growth of Eucalyptus Fusarium wilt.

Different control measures like cultural practices, chemical, and biological approaches are in practice against Fusarium wilt [14] but activation resistance through resistant cultivars is likely the most appropriate one [15]. If the disease appears in epidemic form, then farmers mostly use chemicals due to their quick response and easy availability, but excessive use chemicals cause environmental pollution which affects human health due to their residual effects. On the other hand, continuous use of chemicals causes resistance in pathogens, which may lead to the development of more virulent strains of pathogen due to mutation. This issue forces scientists, researchers, and farmers to search for some alternatives to chemicals. In this scenario, In this scenario, use of plant defense activators is likely the best strategy for plant disease management [16-17] because plant defense activators have the least deleterious effects on the environment, humans, and plants [18]. They activate the plant defense system through the production of phenolic antioxidants in the plant [19]. So, in the current study, different plant defense activators at different concentrations were evaluated towards the Fusarium wilt of eucalyptus.

The use of macro and micro nutrients is another strategy to manage the Fusarium wilt of eucalyptus [20]. Deficiency or excessive use of nutrients is responsible for causing ill effects on the plants by causing a disturbance in the metabolism and physiology of the host plants, which makes the plants vulnerable to pathogens [20-21]. The deficiency of nutrients in plants is the prime cause of the deteriorating defense system of the plants, which makes the plants susceptible to various diseases [22]. The application of a balanced amount of nutrients acts as the first line of defense against invading pathogens by activating the cross-protection mechanism of the host plant through production of different biochemical enzymes and phenolic antioxidants [23].

Due to the detrimental effects of the mentioned disease, it is the need of hour to investigate the role of nutrients, plant defense activators and climatic factors in disease management.

Materials & Methods

Identification and purification of pathogen

Plant parts showing typical symptoms of the disease were collected from Shorkot (Punjab) Irrigated Forest Plantation using the rectangular survey method. The principal species of this plantation (*E. camaldulensis*) suffered from various diseases, but the most dominant one was

Fusarium wilt. Five diseased samples from ten different sites were collected and brought to the Plant Pathology laboratory to isolate pathogens associated with Eucalyptus's wilt. Initially, infected roots were washed with distilled water and then surfaced sterilized (1% Sodium hypochlorite) followed by two washings of distilled water. Then samples were cut into small pieces (0.5-1 cm). Pieces of root were dried on a watch glass with the help of sterilized filter paper and were placed in sterilized Petri plates containing potato dextrose agar medium (PDA) (Water = 1000 mL, Potato = 200 g, Dextrose = 20 g, Agar = 20 g autoclaved at 121 ^{o}C and 15 PSI for 15 minutes) and 48-72 hrs. of incubation at 30at ^{o}C to promote fungal development [24]. The pathogen was then purified by transferring the fungal colony into another Petri plate containing PDA. After five days, the pathogen was identified based on morphological and taxonomical characteristics (colony color, pattern of fungal growth, shape, and size of spore) through microscopy and pathogenicity test [25].

Pathogenicity test

A pathogenicity test of *F. oxysporum* inoculum was conducted to fulfill Koch's postulates. For this purpose, seedlings of *E. camaldulensis* (10-16 cm height) were collected from the nursery of Department of Forestry and Range Management, UAF and transferred into pots (30 cm diameter) containing sandy loam soil, @ two seedlings/pot under Completely Randomized Design (CRD) and were kept under strict and careful observation in the greenhouse, situated in the Research Area, Department of Forestry and Range Management. 12 plants of 60 days' age were used for the pathogenicity test. Among these plants, 9 plants were treated with inoculum, while 3 plants were only drenched and sprayed with distilled water. Plants were inoculated through soil drenching and spraying method [26] by using $1×10^6$ spores/ mL of water, measured with the help of a hemocytometer (ZNC-30). Symptoms appeared after ten days of inoculation. Then the pathogen was re-isolated from artificially inoculated eucalyptus plants to fulfill Koch's postulates [27].

Assessment of nutrients against Fusarium wilt of *E. camaldulensis* under greenhouse conditions

Earthen pots (30 cm) were filled with homogeneous sterilized soil with a 1:1:1 ratio (sand, silt and farmyard manure) and six-month-old seedlings of *E. camaldulensis* were transferred into these earthen pots. Seedlings were arranged under Completely Randomized Design (CRD) with five replications under greenhouse conditions, situated in the Research Area Department of Forestry and Range Management. Seedlings were inoculated (only one time) with a spore suspension of

Commented [A4]: Added full stop after word management

Fusarium oxysporum @1×10^6 spores/mL of H_2O, which was adjusted by using a hemocytometer (ZNC-30) through soil drenching and spraying method. Four treatments namely T_1 [compound (N=20%, P=20% and K= 20%)], T_2 [Nutriotop (Fe= 30%, B = 10%, Cu=, 10% Zn= 40%, Mn= 10%)] and T_3 [combination of T_1+ T_2] while fourth treatment (T_4) was distilled water only. All the nutrients were applied in liquid form under controlled conditions to induce resistance towards Fusarium wilt of *E. camaldulensis* under greenhouse conditions (FW). T1 @ 5g, 7.5g, 10 g/L, T_2@ 0.25, 0.5, 0.75g/L and combination of T_1+ T_2 = (T_3) along with control (T_4) were evaluated towards wilt of Eucalyptus. Data regarding incidence was noted throughout the season with one-week intervals [28] by using the following formula.

Disease incidence (%) = [(No. of infected plants) / (No. of total plants)] × 100

Establishment of Fusarium infested field in the Research Area Department of Forestry and Range Management

Fusarium infested field was established for the evaluation of different nutrients and plant defense activators as a management approach toward wilt. For this purpose, an area of 400 m^2 was selected in the Research Area, Department of Forestry and Range Management, which was continuously cultivated with Eucalyptus seedlings for the last five years. Two sprays of inoculum were done on the selected area consecutively to enhance the population pressure of Fusarium in the soil when assessing the efficacy of plant defense activators and nutrients towards Fusarium wilt of eucalyptus. Seedlings of eucalyptus were planted in the field and again infused with spore suspension @ 1×10^6 spores/mL of H_2O. This spore concentration was measured by using a hemocytometer. The spores in this solution were made by mixing 3-4 mL of distilled water in a Petri plate ~~having~~has a 7–10-days old culture of inoculum. It was poured into a beaker containing 1000 mL of water and shaken well and drenched near the seedlings' root zone to enhance the amount of inoculum for disease establishment.

Assessment of plant activators against Fusarium wilt of *E. camaldulensis* under field conditions

Five plant activators, i.e., Salicylic acid ($C_7H_6O_3$), Benzoic acid (C_7H_6O~~$_3$)~~$_2$), Citric acid ($C_6H_8O_7$), Dipotassium phosphate (K_2HPO_4), Monopotassium phosphate (KH_2PO_4) along with control @ 0.25, 0.75 and 1% with three replications were applied against Fusarium wilt. For this purpose, surface sterilized seeds of Eucalyptus with a 1% solution of sodium hypochlorite were sown under field conditions attributed by Randomized Complete Block Design (RCBD) in the sick field. When

Commented [A5]: C7H6O2

the seedlings were six months old, all the plant activators at three different concentrations were applied to create resistance in Eucalyptus plants through soil drenching and spray methods. To enhance inoculum pressure, an amount of $1x10^6$ spores/ mL of H_2O was applied to the plants for the development of wilt disease. Upon appearance of disease symptoms, data regarding disease incidence was recorded by using the following formula with one-week intervals throughout the season.

$$\text{Disease incidence (\%)} = (\text{No. of infected plants}) / (\text{No. of total plants}) \times 100$$

Impact of plant activators and nutrients on the number of leaves, fresh and dry weight

Data regarding no. of leaves and fresh and dry weight of leaves (g) was recorded with the help of electric balance (AEL-223) after the application of plant activators (i.e., Salicylic acid, Benzoic acid, Citric acid, Dipotassium phosphate Monopotassium phosphate). These activators along with control at three different concentrations were evaluated @ 0.25, 0.75 and 1% and nutrients (Compound and Nutriotop) alone and in combination at three concentrations while control plants were treated only with distilled water. Data regarding the increase in the number of leaves and their fresh and dry weight were recorded at the end of the season. Data regarding number of leaves, fresh and dry weight was recorded after 10, 20, 30 days of intervals.

Characterization of climatic factors favoring Fusarium wilt of *Eucalyptus camaldulensis*

To determine the impact of epidemiological factors on the development of *Fusarium wilt*, seedling of *Eucalyptus camaldulensis* were transplanted in the Research Area, Department of Forestry and Range Management under natural field conditions and data regarding disease incidence was recorded for the whole season. No artificial inoculation was done on seedlings as these were planted in an already infested field. Data regarding environmental factors like maximum and minimum temperature (^{o}C), wind speed (Km/h), rainfall (mm), relative humidity (%) and pan evaporation (%) was collected from the metrological observatory situated at the Research Area, Department of Agronomy, University of Agriculture, Faisalabad. The influence of these environmental factors was observed on the disease development through correlation [29]. Regression analysis identified the most conducive environments for disease progression.

Data analysis

All statistical tests regarding the impact of epidemiological factors like temperature (max. and mini), relative humidity, rainfall, wind speed, pan evaporation, different nutrients, and plant defense activators on Fusarium wilt of *Eucalyptus camadulensis* were performed by using

Formatted: Justified, Indent: Hanging: 0.43"

Formatted: Justified, Indent: Hanging: 0.43", Line spacing: 1.5 lines

Formatted: Font: 12 pt

Formatted: Normal, Justified, Indent: Left: 0.11", Line spacing: 1.5 lines, No bullets or numbering

Commented [A6]: NEW line added here

SAS/STAT statistical software (SAS Institute, 1990). A greenhouse experiment was conducted under CRD, while field experiments were conducted under RCBD. Means were separated using Fisher's protected Least Significant Difference (LSD) procedure by taking a 5% probability level [29]. Analysis of variance (ANOVA), the interaction of different treatments and their combinations were developed by using SAS/STAT software package (SAS Institute, 1990).

Results

Symptomology and development of Fusarium wilt of *E. camaldulensis* under field conditions

Younger leaves showed vein clearing, marginal tissue necrosis, and yellowing, whereas older plants showed withering of leaves followed by falling and eventually mortality. The soil-borne disease invaded the plants via vascular systems. The disease started to appear during the last week of April and reached its peak during June-July (Figure. 1 a, b, c).

<<Insert Figure. 1 a, b, c here>>

Effect of Compound and Nutriotop at different concentrations towards wilt, dry wight, fresh weight and no. of leaves of *E. camaldulensis* under field conditions

Nutrients were evaluated alone and in combination against the fusarium wilt of Eucalyptus. Minimum disease incidence was exhibited by nutrients provision in combination, i.e., compound+ nutriotop (9.567%) followed by compound (16.88 %) and nutriotop (16.88%) as compared to control (Figure.2). In the case of interaction between treatments and their concentrations, minimum disease incidence was expressed by nutrients provision in integration, i.e., compound + nutriotop (10.20, 9.40, 9.10%) followed by compound (20,17,14%), nutriotop (22,19,14%) as compared to control (30.20%) (Figure. 1c & 2). Maximum dry weight of leaves was observed when nutrients were applied in combination (4.677 g) followed by nutriotop (3.167 g) and compound (3.012 g) as compared to control. While in case of interaction b/w treatment and concentration combination of compound + nutriotop exhibited maximum dry weight followed by nutriotop and compound as compared to control, similar observations were noted in the case of fresh weight and number of leaves after application of nutrients alone, in combination and interaction between treatments and concentrations (Figure. 3).

<<Insert Figure. 2 here>>

<<Insert Figure. 3 here>>

Effect of plant activators on fusarium wilt occurrence, fresh, dry weight, and number of leaves of *E. camaldulensis* under field conditions

Among all plant activators, the minimum incidence of fusarium wilt was 11.383% when Salicylic acid was used, followed by Dipotassium Hydrogen Phosphate, Potassium Dihydrogen Phosphate, Benzoic acid, and Citric acid (15.11, 17.556, 17.773 and 18. 567%) respectively as compared to control (Figure 4). While in the case of interaction b/w treatments and concentrations maximum incidence of disease was expressed by Benzoic acid (24.567, 16,13%) @ 0.5, 0.75 and 1%, respectively, while minimum disease incidence was exhibited by Salicylic acid (13.66,12.33 and 8%) at three concentrations respectively (Fig.4) as compared to control. The maximum no. of leaves, fresh and dry weight of leaves expressed by Salicylic acid, followed by Dipotassium Hydrogen Phosphate, Potassium Dihydrogen Phosphate, Benzoic acid, and Citric acid alone and in interaction with all concentrations, respectively (Figure. 5) as compared to control.

Commented [A7]: % added

<<Insert Figure. 4 here>>

<<Insert Figure. 5 here>>

Characterization of environmental conditions conducive for the development of Fusarium wilt of *E. camaldulensis* under field conditions

Disease symptoms started to appear at 25°C and it continuously increased up to 42 ^{o}C (maximum temperature) as indicated by R^2= 0.6488. Maximum disease incidence was observed b/w 35 – 40 °C. The regression equation indicated that with increase in one-unit temperature, the disease increased by 1.373 units. A significant positive relationship was noted in the case of minimum temperature (R^2=0.5694). Increased in disease incidence was noted b/w 6-28°C, and maximum disease was noted b/w 6-12 ^{o}C under minimum temperature. Relative humidity and wind speed likewise expressed positive relation with the Fusarium wilt with R^2= 0.8258 and 0.5867, respectively. Progress in disease was noted b/w 60 -90 % relative humidity and 1-3.5 mm rainfall. Maximum disease in case of relative humidity was observed b/w 70-80% and 1.5 -2.5 km/h wind speed. While pan evaporation expressed a very weak relationship with disease development, as shown in regression models (Figure.6) and scattered plots of maximum, and minimum temperature, relative humidity, rainfall, wind speed and pan evaporation (Figure. 7). Maximum disease was noted during the first week of September (Figure 8).

<<Insert Figure. 6 here>>

<<Insert Figure. 7 here>>

<<Insert Figure. 8 here>>

Evaluation of nutrients at different concentrations towards wilt, dry wight, fresh weight and no. of leaves of *E. camaldulensis* under greenhouse condition.

Nutrients were evaluated alone and in combination against the fusarium wilt of Eucalyptus. Minimum disease incidence was exhibited by nutrients provision in combination, i.e., compound+nutriotop followed by compound and nutriotop as compared to control (Figure. 9). In the case of interaction between treatments and their concentrations, minimum disease incidence was expressed by nutrients provision in integration, i.e., compound + nutriotop followed by compound, ~~nutriotop as~~nutriotop as compared to control (Figure. 9 & 10). Maximum dry weight of leaves was observed when nutrients were applied in combination followed by nutriotop and compound as compared to control. While in case of interaction b/w treatment and concentration combination of compound + nutriotop exhibited maximum dry weight followed by nutriotop and compound as compared to control, similar observations were noted in the case of fresh weight and number of leaves after application of nutrients alone, in combination and interaction between treatments and concentrations (Figure. 10). A Fisher's least significant difference (LSD) .05 was calculated to determine differences among the treatment means (P<0.05) for each variable.

<< Insert Figure. 9 here >>

<< Insert Figure. 10 here >>

Discussion

Fusarium wilt is a major issue in Eucalyptus growing areas in Pakistan. Main characteristic symptoms of this disease are vascular discoloration, chlorosis, and wilting [30]. There are many factors that affect disease development such as inoculum density, population, host range and time of infection [31]. Temperature, pH, carbon, and nitrogen availability also influence the dissemination of disease symptoms [32]. Similarly, several other factors like environment, infectious pathogen and host range are involved in the occurrence of disease [33]. The most effective and practical strategy for lowering the prevalence of soil-borne diseases is the use of resistance sources [33-34]. Furthermore, the use of a resistance source decreases the incidence of Eucalyptus wilt and avoids fungicide toxicity [34].

Evaluation of nutrition towards incidence of fusarium wilt, number of leaves along with fresh and dry weight

Physiological functions of plants are destroyed by the effect of pathogen, such as movement of important nutrients, their absorption, translocation, and utilization from plant roots towards shoot system [35-36] as nutrients play a vital role in plants. The most important purpose of nutrients is to maintain plant health by regulating different cellular functions through the activation of metabolism, making plants more resistant to pathogen attack [37]. So, the suitable accessibility of micro (Zn, Mn and Fe, Cu and B) and macronutrients (NPK) decrease the incidence of plant diseases because nutrients structurally stabilize the protein molecule, whereas majority of them are catalytically active co-factor in enzymes [38, 39]. Nutrient availability in plants is reduced due to

Formatted: Font: 12 pt
Formatted: Font: 12 pt
Formatted: Font: (Default) Times New Roman, 12 pt
Formatted: Font: 12 pt
Formatted: Font: 12 pt
Formatted: Font: (Default) Times New Roman, 12 pt
Formatted: Font: 12 pt
Formatted: Font: 12 pt
Formatted: Font: 12 pt
Formatted: Font: (Default) Times New Roman, 12 pt
Formatted: Font: 12 pt
Formatted: Font: 12 pt
Formatted: Font: (Default) Times New Roman, 12 pt
Formatted: Font: 12 pt
Formatted: Font: (Default) Times New Roman, 12 pt
Formatted: Font: 12 pt
Formatted: Font: 12 pt
Formatted: Font: (Default) Times New Roman, 12 pt
Formatted: Font: 12 pt
Formatted: Font: 12 pt
Formatted: Font: (Default) Times New Roman, 12 pt
Formatted: Font: 12 pt
Formatted: Font: (Default) Times New Roman, 12 pt
Formatted: Font: 12 pt
Formatted: Font: (Default) Times New Roman, 12 pt
Formatted: Font: 12 pt
Formatted: Font: (Default) Times New Roman, 12 pt
Formatted: Font: 12 pt
Formatted: Font: (Default) Times New Roman, 12 pt
Formatted: Font: 12 pt
Commented [A8]: Greenhouse experiment has been added here
Formatted: Font: 12 pt
Formatted: Font: 12 pt
Formatted: Font: 12 pt
Formatted: Font: 12 pt
Formatted: Justified

the attack of pathogen, so this nutrient deficiency deteriorates the plant's growth, development, and defense system [40]. In present studies, nutriotop (Fe, Cu, Zn, B and Mn) solo and in grouping with the composite mixture (NPK) was assessed against Fusarium wilt disease of Eucalyptus. Results demonstrated that compounds with nutriotop amplified resistance in plants, ultimately decreasing the incidence of Fusarium wilt.

Nitrogen is the most important part of plant nutrients as it is an integral part of different molecules such as protein, amino acid, nucleic acid, chlorophyll cytosine and auxin [41], plants fulfill their nitrogen deficiency from nitrate and ammonium ions (soil). When a pathogen attacks Eucalyptus seedlings, it snatches nitrogen from the plant. Consequently, plant growth is inhibited by the attack of *Fusarium oxysporum* [42]. Similarly, some other nutrients like Zinc (Zn), Boron (B), and

Phosphorus (P) have a critical role in maintaining plant health. Phosphorus is important component of ATP, DNA, RNA, and cell membrane [39]. Zinc is also a significant element that performs a fundamental responsibility in the production of nitrogen and carbon-based components. Sugar levels and amino acid pro-duction in plant tissue are reduced by the low level of Zinc. It also detoxifies superoxide radicals, which protects the cell membrane from oxidative damage. [43]. Critical damage to the cell membrane caused by free radicals can collapse the cell membrane, ultimately facilitating food delivery to the pathogen [44].

In the current study, the application of Zinc reduced disease and increased the amount of foliage and their dry and fresh weights, which is supported by the work of [45]. Current investigation was in line with previous study in which the rate of nitrogen application significantly impacted on plant biomass and Fusarium wilt severity. [46]. Recent findings were advocated by previous study in which minimum disease incidence was observed by the application of micronutrients in combination and significant increase in plant biomass was also noticed [47].

Impact of plant activators on Fusarium wilt of Eucalyptus, number of leaves along with their fresh and dry weights

The defense systems of plants become inactivated by the attack of pathogens, and the chance of disease enhancement increases. For the initiation of defense system in plants, resistance genes play crucial role towards pathogen. Use of growth regulators activate the basal defense system of the host plant [48, 49], and susceptible plant become resistant [50]. Accumulation of indole acetic acid and salicylic acid enhanced the growth and resistance of plants [51]. Moreover, the action of resistance genes was increased by the adequate quantity of salicylic acid [52].

In the present study, the role of five different plant activators, Salicylic acid ($C_7H_6O_3$), Benzoic acid ($C_7H_6O_3$), Citric acid ($C_6H_8O_7$), Dipotassium phosphate (K_2HPO_4), Monopotassium phosphate (KH_2PO_4) was investigated towards the occurrence of Fusarium wilt in Eucalyptus trees and least disease incidence was expressed by applying salicylic acid. The recent study agreed with [53-54, 24], who estimated different growth regulators against Fusarium wilt, i.e., Salicylic acid, Ferric chloride, Hydrogen peroxide, Dipotassium hydrogen orthophosphate, Indole acetic acid and Calcium chloride, but Salicylic acid revealed prominent findings in response to Fusarium wilt. Therefore, by the activation of reactive oxygen species (ROS) and fluctuation in cell wall components, salicylic acid is activated in plant defense through this process [55-57], and it increases the production of various proteins [58-60]. Hence, plants expressed minimum disease

with a maximum number of leaves and maximum amount of fresh and dry weights, as observed in the contemporary study. Salicylic acid-induced production of Hydrogen peroxide (H_2O_2) is vital part of ROS. Hydrogen peroxide retarded the fungal growth due to antifungal activity and contributed to the amplification of phenoxy-radicals in plant cells [61-62]. Although the breakdown of the membrane is started by Lipid peroxidation [63]. Conversely, antioxidant defense is a major immune system in plants that protects them from oxidative stress and suppresses the partial production of ROS [64-65].

Impact of climatic factors on the incidence of Fusarium wilt of *E. camaldulensis*

Climate change is an issue of global concern [66]. Climate change has the potential to initiate positive and negative interacting processes which affect forests [67]. For example, maximum concentrations of CO2 in the atmosphere increase the tree's growth rate and water use efficiency [68], and it influences the host-pathogen interactions. Hence, this changing climate also impacted the behavior and distribution of pathogens. Some pathogens (including fungi and bacteria), directly and indirectly, are affected by climate. Under favorable environmental conditions, the pathogen's life cycle is directly affected by temperature and moisture because most pathogens are sensitive to precipitation and moisture. While the rate of reproduction, spreading, and infection are higher when conditions become conducive for them. Similarly, when plants are under stress, pathogens are indirectly affected by environmental factors to their host plants [68-69].

Meanwhile, it's very difficult to judge whether climate alone causes tree mortality, as the number of reports regarding dieback, decline and mortality attributed to climate drivers is increasing. If a tree died due to heat and drought, this phenomenon is associated with climate change, but irregular droughts have long been involved in mortality [69]. If an increase in mortality occurred in all trees, then it may be due to climate change. Some forested ecosystems of the world may already be affected by climate change, and a further rise in mortality rate is assumed to be linked to a scarcity of water [70]. Changing atmospheric composition and climate can also modify the plant canopy. Elevated CO_2 fertilization stimulates plant growth morphology, physiology, chemical composition and gene expression [71]. These morphological changes can include increased plant height, number of branches and tillers, thickness, and area of leaves.

The growth of plants is influenced by minimum soil and air temperature (^oC), soil moisture (%), wind speed and (km/h) rainfall (mm) which are the vital causes that affect the plants for the infection growth [72-73]. Sudden changes in climatic factors can boost the resistance and

susceptibility of host plant [74-75]. As these environmental factors bring various changes in pathogens, such as reproduction, growth rate, host-pathogen interaction, infection, and dissemination [10, 76]. Results of the current study showed a significant relationship between temperature (maximum and minimum), pan evaporation, wind speed and rainfall with disease development which is supported by the findings of Cherian and Varghese [77]. These findings also agree with [78-80, 30, 56], who reported that hot and humid weather positively impacted the prevalence of disease. A similar finding was also reported by [81-83, 12]. Increase in temperature amplified the multiplication rate of the pathogen [84]. This rapid multiplication of pathogens causes a direct effect on roots which results in the destruction of plants [85-86]. It was also observed that when the temperature was raised from 35°C, the amount of HNO_2 and NH_3 was also raised after the disease attack [86]. Furthermore, the production of HNO_2 and NH_3 increased the pathogen's intensity and revitalized the pathogen's virulence [87-88].

The results of the present study are helpful for Eucalyptus growers in managing fusarium wilt disease through the knowledge of conducive environmental variables. Because before contemporary study, no work was done on determining suitable environmental conditions for this disease. No doubt, the results of the current study are useful for farmers. Still, it is need of the hour to design such a type of study on mass scale to know the intensity and severity for developing the most reliable disease-predictive model and to validate the results under field conditions. It requires at least three to five years of data of disease and environmental factors.

Conclusion

Conclusively we can say that Fusarium wilt of *Eucalyptus camaldulensis* can be managed by plant activators, macro (NPK) and micronutrients (Fe, Zn, Cu, B, Mn) by keeping in view the environmental factors. Among all the treatments, salicylic acid and a combination of compound + nutriotop exhibited minimum disease incidence and the best growth response. Climate has a key role in the dissemination of Fusarium species, so the findings of the current study showed maximum disease incidence between 35 – 40 °C (max. T), 6-25°C (mini. T), 70-80% relative humidity, 1.5-2.5 km/h wind speed while pan evaporation expressed weak correlation with disease development.

Commented [A9]: Weak corrected

Future implications of recent revelations for growers

Growers' community can cope with Fusarium wilt of *Eucalyptus camaldulensis*, a formidable challenge to agroforestry by using currently investigated fruitful management tool.

Acknowledgements

We are thankful to the nursery staff of the Department of Forestry and Range Management and the Postgraduate Lab. of the Department of Plant Pathology, University of Agriculture, Faisalabad.

References

1. Minhas, P. S.; Yadav, R.K.; Bali, A. Perspectives on reviving waterlogged and saline soils through plantation forestry. Agricultural Water Management 2020, 232, p.1060-63.
2. Dawar, S.; Younus, S. M.; Tariq, M.; Zaki, M. J. Use of Eucalyptus sp., in the control of root infecting fungi on mung bean and chickpea. Pakistan Journal of Botany 2007, 39, 975-979.
3. Ferreira, V.; Boyero, L.; Calvo, C. A Global Assessment of the Effects of Eucalyptus Plantations on Stream Ecosystem Functioning. Ecosystem 2019, 22,629-642. doi.org/10.1007/s10021-018-0292-7.
4. Jagger, P. A.; Pender, J. The Role of Trees for Sustainable Management of Less-Favored Lands: The Case of Eucalyptus in Ethiopia. Forest Policy Economics 2003, 5, 83-95.
5. Joshi, M. K. Impact of eucalyptus plantations on ground water availability in south Karnataka. ICID 21st International Congress on Irrigation and Drainage 2011, Tehran, Iran.
6. Inouye, S.; Takizawa, T.; Yamaguchi, H. Antibacterial activity of essential oils and their major constituents against respiratory tract pathogens by gaseous contact. Journal of Antimicrobial and Chemotherapy 2001, 47, 565-573.
7. Salerno, M. I.; Gianinazzi, S.; Gianinazzi-Pearson, V. Effects on growth and comparison of root tissue colonization patterns of *Eucalyptus viminalis* by pathogenic and nonpathogenic strains of *Fusarium oxysporum*. The New Phytologist 2000, 146(2), 317-324.
8. Iori, G. A microsimulation of traders activity in the stock market: the role of heterogeneity, agents' interactions and trade frictions. Journal of Economic Behavior & Organization 2002, 49(2), 269-285.
9. Misra, A. K.; Yadav, S. B.; Mishra, S. K.; Tripathi, M. K. Impact of meteorological variables and climate change on plant diseases. In Plant Pathogens 2020, 313-327. Apple Academic Press.

10. Saremi, H.; Burgess, L. W.; Backhouse, D. Temperature effect on the relative abundance of Fusarium species in a model plant soil ecosystem. Soil Biology and Biochemistry 1999, 31, 941-947.

11. Madhavi, G. B.; Bhattiprolu, S. L. Evaluation of fungicides, soil amendment practices and bioagents against *Fusarium solani* - causal agent of wilt disease in chilli. Journal of horticultural science 2011, 6, 141-144.

12. Land, B. B.; Cortes, J. A. N.; Diaz, M. J. Integrated management of Fusarium wilt of Chickpea with sowing date, host resistance and biological control. Journal of Phytopathology 2006, 94, 946-960.

13. Larkin, R. P.; Fravel, D. R. Effects of Varying Environmental Conditions on Biological Control of Fusarium Wilt of Tomato by Nonpathogenic Fusarium spp. Phytopathology 2002, 92(11):1160-6.

14. Kamal, A. M.; Elyousr, A.; Hashem, M. M. Biological control of Fusarium wilt in tomato by plant growth promoting yeasts and rhizobacteria. Plant Pathology Journal 2009, 25,199-204.

15. Nelson, R.; Wiesner-Hanks, T.; Wisser, R.; Balint-Kurti, P. Navigating complexity to breed disease-resistant crops. Nature Reviews Genetics 2018, 19(1), 21-33.

16. Singh, S.; Kumar, V.; Datta, S.; Dhanjal, D. S.; Singh, J. Plant disease management by bioactive natural products. In Natural bioactive products in sustainable agriculture 2020, 15-29. Springer, Singapore.

17. Koo, Y. M.; Heo, A. Y.; Choi, H. W. Salicylic acid as a safe plant protector and growth regulator. The Plant Pathology Journal 2020, 36(1), 1.

18. Huang, J. S.; Hsu, H. T. Induced resistance in plants In: Huang HC and Acharya SN (eds). Advances in Plant Diseases and pest Management 2003, 237-258.

19. Tuladhar, P.; Sasidharan, S.; Saudagar, P. Role of phenols and polyphenols in plant defense response to biotic and abiotic stresses. In Biocontrol Agents and Secondary Metabolites 2021, 419-441. Woodhead Publishing.

20. Asma, S.; Shahnaz, D.; Marium, T.; Zaki, M. J. Management of root diseases by combination of different soils with fertilizers. Pakistan Journal of Botany 2009, 41(6), 3219-3225.

21. Stenger, L. D.; Libardoni, G.; Wagner, A.; Zanela, J.; Alves, L.T.; Varpechoski, G.O.; Lozano, E.R.; Potrich, M. Essential oils in pathogen resistance induction of *Eucalyptus benthamii* Maiden et Cambage. Ciência Rural 2021, 51.

22. Shrestha, J.; Kandel, M.; Subedi, S.; Shah, K. K. Role of nutrients in rice (*Oryza sativa* L.): A review. Agrica 2020, 9(1), 53-62.

23. Saleem, M. H.; Wang, X.; Ali, S.; Zafar, S.; Nawaz, M.; Adnan, M.; Fahad, S.; Shah, A.; Alyemeni, M. N.; Hefft, D. I.; Ali, S. Interactive effects of gibberellic acid and NPK on morpho-physio-biochemical traits and organic acid exudation pattern in coriander (*Coriandrum sativum* L.) grown in soil artificially spiked with boron. Plant Physiology and Biochemistry 2021, 167, 884-900.

24. Pempee.; Mahabeer S.; Satyadev P.; Poonam K. Effects of Different Temperature, pH and Relative Humidity on the Growth of *Fusarium oxysporum* f. sp. *ciceri* causing Chickpea Wilt. International Journal of Current Microbiology and Applied Sciences 2020, 9, 1381-1388.

25. Soesanto, L.; Utami, D. S.; Rahayuniati, R. F. Morphological characteristics of four Trichoderma isolates and two endophytic Fusarium isolates. Journal of Scientific and Industrial Research 2011, 2, 294-306.

26. Paulino, J. F. D. C.; Almeida, C. P. D.; Gonçalves, G. D. M. C.; Bueno, C. J.; Carbonell, S. A. M.; Chiorato, A. F.; Bechimol-Reis, L. L. Assessment of resistance in common bean to *Fusarium oxysporum* f. sp. phaseoli using different inoculation and evaluation methods. Crop Breeding and Applied Biotechnology 2020, 20.

27. Haq, I. U.; Ijaz, S.; Khan, I. A. A Journey From Koch's Postulates to Molecular System Biology. In Phytomycology and Molecular Biology of Plant–Pathogen Interactions 2022, 1-25. CRC Press.

28. Ashfaq, M.; Iqbal, S.; Mukhtar, T.; Shah, H. Screening for resistance to cucumber mosaic cucumo virus in chilli pepper. Journal of Animal and Plant Sciences 2014, 24(3), 791-795.

29. Steel, R. Analysis of variance I: The one-way classification. Principles and procedures of statistics a biometrical approach 1997, 139-203.

30. Matthew, A.; Lawal, A.; Chindo, P.; Olalekan, B. Outbreak of basal stem root and wilt disease of pepper in northern Nigeria. Journal of Plant Protection Research 2006, 46, 7-14.

31. Rekah, Y.; Shtienberg, D.; Katan, J. Papulation dynamics of *Fusarium oxysporum* f.sp. radices-*lycopersici* in relation to the onset of Fusarium crown and root rot of tomato. European Journal of Plant Pathology 2010, 107, 367-375.

32. Naik, M. K.; Rani, G. S. D.; Madhukar, H. M. Identification of resistant sources against wilt of chilli (*Capsicum annum* L.) caused by *Fusarium solani* (Mart.) Journal of Mycopath Research 2008, 46, 93-96.

33. Saremi, H.; Amiri, M. E. Exploration of potato cultivar resistant to the major fungal pathogen on the potato wilting disease in Iran. Journal of Food Agriculture and Environment 2010, 8, 821-826.

34. Naik, M. K.; Madhukar, H. M.; Rani, G. S. D. Evaluation of fungicides against *Fusarium solani*, the causal agent of wilt of chilli. International journal of vegetable science 2007, 34, 173-176.

35. Stewart, M. W.; Dibb, W. D.; Johnston, E. A.; Smyth, J. T. The contribution of commercial fertilizer nutrients to food production. Agronomy Journal 2005, 97, 1-6.

36. Khan, A.; Shah, S. N. M.; Rab, A.; Sajid, M.; Ali, K.; Ahmed, A.; Faisal, S. Influence of nitrogen and potassium levels on growth and yield of chillies (*capsicum annum* L.). International Journal of Farming and Allied Sciences 2014, 3, 260-264.

37. Saikia, R.; Varghese, S.; Singh, B. P.; Arora, D. K. Influence of mineral amendment on disease suppressive activity of Pseudomonas fluorescens to Fusarium wilt of chickpea. African Journal of Microbiological Research 2009, 164, 365-373.

38. Allabi, D. A. Effect of fertilizer phosphorus and poultry droppings treatments on growth and nutrition components of pepper (capsicum annum L). African Journal of Biotechnology 2006 5(8), 671-677.

39. Suharaja; Sutarno. Biomass Chlorophyll and nitrogen content of leaves of two chilli pepper varieties (Capsicum annum) in different fertilizer treatments. Nusantara Bioscience 2009, 1, 6-16.

40. Sanjeev, K.; Eswaran, A. Efficacy of Micronutrients on Banana fusarium Wilt. (*Fusarium oxysporum* f. sp. *cubense*) and it's Synergistic Action with *Trichoderma viride*. Notulae Botanicae Horti Agrobotanici Cluj-Napoca 2008, 36(1), 52-54.

41. Guertal, E. A. Preplant slow-release nitrogen fertilizer produce similar bell pepper yields as split applications of soluble fertilizers. Agronomy Journal 2000, 92, 388-393.

42. Chellemi, D. O.; Lazarovits, G. Effects of organic fertilizer application on growth yield and pest of vegetable crop. Proceedings of Florida State Horticultural Society 2002, 115, 315-321.

43. Cakmak, I. M. Possible roles of zinc in protecting plant cell from damage by reactive oxygen species. New Phytologist 2000, 146, 185-205.

44. Mengel, K.; Kirkby, E. A. Principles of Plant Nutrition, 5th (ed.) Kluwer, Amsterdam, Netherlands 2001, pp 847.

45. Grewal, H. S.; Graham, R. D.; Rengel, Z. Genotypic variation in zinc efficiency and resistance to crown rot disease (*Fusarium graminearum* Schw. Group 1) in wheat. South African Journal of Plant and Soil 1996, 186, 219-226.

46. Orr, R. P.G.; Dennis ,Y.; Wong ,D.J.; Browne, M.; Cooper, H.W.; Birt, H.R.; Lapis-Gaza, A.B.; Pattison, P.N. Nitrogen fertilizer rate but not form affects the severity of Fusarium wilt in banana. Frontiers in plant science 2022, 13,907-819.

47. Abd-El-Rahman, S.; Mazen, M.; Khalil, A. Integrated suppressive effect (s) of micronutrients and compost against chickpea Fusarium wilt in relation to microbial activity. Egyptian Journal of Phytopathology. 2014, 42, 59-77.

48. Jalali, B. L.; Bhargava, S.; & Kamble, A. Signal transduction and transcriptional regulation of plant defense responses. Journal of Phytopathology 2006, 154, 65-74.

49. Inoue, I.; Namiki, F.; Tsuge, T. Plant colonization by the vascular wilt fungus *Fusarium oxysporum* requires FOW1, a gene encoding a mitochondrial protein. The Plant Cell 2002, 14(8), 1869-1883.

50. Elwan, M. W. M.; EL-Hamahmy, M. A. M. Improved productivity and quality associated with Salicylic acid application in greenhouse pepper. Scintia Horticulturea 2009, 122, 521-526.

51. Hayat, S.; Fariduddin, Q.; Ali, B.; Ahmad, A. Effect of salicylic acid on growth and enzyme activities of wheat seedlings. Acta Agronomica Hungarica 2005, 53, 433-437.

52. Khan, W.; Prithviraj, B.; Smith, D. L. Photosynthetic responses of corn and soybean to foliar application of salicylates. Journal of Plant Physiology 2003, 160, 485-492.

53. Hanieh, A.; Mojtaba, D.; Zobihollah, Z.; Vahid, A. Effect of pre-sowing salicylic acid seed treatments on seed germination and growth of greenhouse sweet pepper plants. Indian Journal of Science and Technology 2013, 6, 3868-3871.

54. Ali, Z.; Smith, I.; Guest, D. I. Combinations of potassium, phosphonate and bion (Acibenzolar-S-methyl) reduce root infection and dieback of *Pinus radiata*, *Banksia integrifolia* and *Isopogon cuneatus* caused by *Phytophthora cinnamomi*. Australas Plant Pathology 2000, 29, 59-63.

55. Colson-Hanks, E. S.; Allen, S. J.; Deverall, B. J. Effect of 2, 6-dichloroisonicotinic acid or benzothiadiazole on Alternaria leaf spot, bacterial blight and Verticillium wilt in cotton under field conditions. Australas Plant Pathology 2000, 29, 170-177.

56. Agrios, G. N. Plant Pathology 4th edition. Academic Press, London 2005, p. 442-445.

57. Govindappa, M.; Rai, V. R.; Lokesh, S. In-vitro and in-vivo responses of different treating agents against wilt disease of safflower. Journal of Cereals and Oilseeds 2011, 2, 16-25.

58. Hayat, S.; Ahmad, A. Salicylic acid: A plant hormone 2007, Springer. 401.

59. EL- Yazied, A. A. Effect of foliar application of salicylic acid and chelated Zinc on growth and productivity of sweet pepper (*capsicum annum* L.) under autumn planting. Research Journal of Agriculture and Biology 2011, 7, 423-433.

60. Biswas, S. K.; Paandey, N. K.; Rajik, M. Induction of defense response in tomato against Fusarium wilt through inorganic chemicals as inducers. Journal of Plant pathology and Microbiolog 2012, 4, 128-135.

61. Inbar, M.; Doostdar, H.; Sonoda, K. M.; Leibee, G. L.; Mayer, R. T. Elicitors of plant defensive system reduced insect densities and disease incidence. Journal of chemical Ecology 1998, 24, 135-149.

62. Huynh, Q.K.; Borgmeyer, J. R.; Smith, C. E.; Bell, L. D.; Shah, D. M. Isolation and characterization of a 30 kDa protein with antifungal activity from leaves of *Engelmannia pinnatifida*. Journal of Biology Chemistry 2001, 316, 723-727.36.

63. Lee, J.; Leslie, J. F.; Bowden, R. L. Expression and function of sex pheromones and receptors in the homothallic ascomycete *Gibberella zeae*. Eukaryotic Cell 2006, 7, 1211-1121.

64. Sitara, U.; Hasan, N. Studies on the efficacy of chemical and non-chemical treatments to control mycoflora associated with chilli seed. Pakistan Journal of Botany 2011, 43, 95-110.

65. Torres-Castillo, J. A.; Sinagawa-GarcIa, S. R.; Martinez-Avila, G. C. G.; Lopez-Flores, A. B.; Sanchez-Gonzalez, E. I.; Aguir-re-Arzola, V. E.; Torres-Acosta, R. I.; Olivares-Saenz, E.; Osorio-Hernandez, E.; Gutierrez-Diez, A. *Moringa oleifera:* phyto-chemical detection, antioxidants enzymes and antifungal properties. Phyton 2013, 82, 193-202.

66. Bhatti, J. S.; Lal, R., Apps M. J.; Price, MA. Climate Change and Managed Ecosystems. Boca Raton. Florida 2006, USA, 201-216.

67. Williams, A. P.; Allen, C. D.; Millar, C. I.; Swetnam, T. W.; Michaelsen, J.; Still, C. J.; Leavitt, S. W. Forest responses to in-creasing aridity and warmth in the southwestern United States. Proceedings of the National Academy of Sciences 2010, 107, 21289-21294.

68. Boisvenue, C.; Running, S. W. Simulations show decreasing carbon stocks and potential for carbon emissions in Rocky Mountain forests over the next century. Ecology Application 2010, 20(5),1302-1319.

69. Van Mantgem, P. J.; Stephenson, N. L.; Byrne, J. C.; Daniels, L. D.; Franklin, J. F.; Fulé, P. Z.; Veblen, T. T. widespread in-crease of tree mortality rates in the western United States. Journal of Science 2009, 323, 521-524.

70. Allen, C. D.; Macalady, A. K.; Chenchouni, H.; Bachelet, D.; McDowell, N., Vennetier, M.; Gonzalez, P. A global overview of drought and heat-induced tree mortality reveals emerging climate change risks for forests. Forest Ecology and Management 2010, 259(4), 660-684.

71. Matros, A.; Amme, S.; Kettig, B.; Buck0Sorlin, G. H.; Sonnewald, U. W. E.; Mock, H. P. Growth at elevated CO2 concentrations leads to modified profiles of secondary metabolites in tobacco cv. SamsunNN and to increased resistance against infection with potato virus Y. Plant Cell Environment 2006, 29, 126-137.

72. Larkin, R. P.; Fravel, D. R. Effects of varying environmental conditions on biological control of Fusarium wilt of tomato by nonpathogenic Fusarium spp. Journal of Phytopathology 2002, 92, 1160-1166.

73. Bakhsh, A.; Iqbal, S. M.; Haq, I.K. Evolution of chickpea germplasm for wilt resistance. Pakistan journal of Botany 2007, 39(2), 583-593.

74. Chakaraborty, S.; Pangga, I. B. Plant disease and climate change. In: Gillings M, Homles A, eds. Plant Microbiology, London UK, BIOS Scientific Publication 2004, 163-180.

75. Ahmad, I.; Khan, R.A.; Siddiqui, M. T. Incidence of dieback disease following fungal inoculations of sexually and asexually propagated Shisham (*Dalbergia sissoo*). Forest Pathology 2013, 43, 77-82.

76. Ghini, R.; Hamada, E.; Bettio, W. Climate change and plant diseases. Journal of the Science of Food and Agriculture 2008, 65, 98-107.

77. Cherian, T.T.; Varghese, M. K. I. Post-harvest fungal rot of chili (*Capsicum annum* L.) fruits. Journal of Mycology and Plant Pathology 2005, 35, 97-99.

78. Zakaria, M. J.; Lockwood, J. L. Reduction in Fusarium populations in soil by oilseed meal amendments. Journal of Phyto-pathology 1980, 70, 240-243.

79. Abawi, G. S.; & Barker, K. R. Effect of cultivar, soil temperature and levels of Meloidogyne incognita on root necrosis and Fusarium wilt of tomatoes. Journal of Phytopathology 1984, 74, 433-438.

80. Khilare, V. C.; Ahmed, R. Effect of different media, pH and temperatures on the growth of *Fusarium oxysporum* f. sp. *ciceri* causing chickpea wilt. International Journal of Advance Biological Research 2012, 2, 99-102.

81. Westerlund, J. F. V.; Campbell, R. N.; Kimble, K. A. Fungal root rots and wilt of chickpea in California. Journal of Phyto-pathology 1974, 64, 432–436.

82. Karimi, R.; Owuoche, J. O.; Silim, S. N. Importance and management of Fusarium wilt (*Fusarium udum* Butler) of pigeonpea. International Journal of Advance Agricultural Research 2012, 2, 1-14.

83. Mehmood, Y.; Khan, M.A.; Javed, N.; Arif, M.J. Effect of soil and environmental factors on chickpea wilts disease caused by *Fusarium oxysporum* f. sp. *ciceris*. Pakistan Journal of Phytopathology 2013, 25, 52-58.

84. Burgess, L. W.; Summerell, B. A.; Walsh, J.; Petrovic, T.; Backhouse, D. Mycogeography, ecology and taxonomy of Fusarium. Journal of Plant Pathology 2008, 90, 3-7.

85. Emberger, G.; Weltry, R. E. Effect of soil water matric potential on resistance to *Fusarium oxysporum* f. sp. *medicaginis* in alfalfa. Journal of Phytopathology 1983, 73, 208-212.

86. Senthilkumar, G.; Madhanraj, P.; Panneerselvam, A. Studies on saprophytic survival of *Fusarium oxysporum* using pre-colonized paddy straw bits. Journal of Natural Product and Plant Resource 2011, 1, 15-19.

87. Megie, C. A.; Pearson, R. W.; Mitbold, A. E. Toxicity of decomposing crop residues to cotton germination and seedling growth. Agronomy Journal 1967, 59, 197-199.

88. Benjumea, A. B.; Basallote-Ureba, M. J.; Abbasi, P. A.; Lazarovits, G.; Melero-Vara, J. M. Effects of incubation temperature on the organic amendment-mediated control of Fusarium wilt of tomato. Annals of Applied Biology 2014, 164, 453-463.

89.

PLOS ONE Reviewer comments

Reviewer 1

Reviewer comments	Responses
1. The title needs to be clarified.	Incorporated
2. I highly recommend reviewing the grammar of the paper.	Grammatical Errors are corrected.
3. Introduction section;<br>(a) Line 50, start the Intro with reference No. [16], How?<br>(b) The references need to be rearranged.	All the references in the text are rearranged according to your prescription.
4. Material and Methods section;<br>(a) Line 106 "ml" change to mL and so on.<br>(b) What specific synthesis techniques are most effective for combining silicon and copper nanoparticles?<br>(c) This method of combination isn't mentioned.<br>(d) Why did you choose these three concentrations (0.025, 0.05, and 0.075 g).<br>(e) Line 182 "ntreated" changed to untreated.	a. Changed to mL and so on.<br>b. Simply the fine powder of both nanoparticles (Cu and Si) was mixed in a beaker and further used for their assessment under laboratory, greenhouse and field conditions.<br>c. It is stated in line 117.<br>d. Nanoparticles cause toxicity to plants at higher concentrations. That's why lower concentrations were most effective for the best results.<br>e. Incorporated.
5. Results;<br>(a) Please, add the UV-vis and SEM figures.	The SEM figures are attached. UV-vis pictures are not available.

Reviewer 2

Reviewer comments	Responses
1. Throughout the manuscript check for English typos	Thoroughly proofread was done to remove any English typos errors.
2. The title is too long, maximum should be 15 words.	Short title will not represent the focus of the research paper clearly, that's why the title is quite longer.
3. the introduction needs more recent literatures, I suggested some.	As per your suggestions, literature provided is recent. Kindly have a look on it. Most of the references are above the year 2020 despite four references.
4. the method should be referenced	Complete methodology is referenced.
5. the figures and tables presenting the data very well, but the discussion should explain why the clear zone is	The suggestion is incorporated and explained why the clear zone is increased with increase in time.

increasing by the time	
6. Statistical analysis should be done with two ways ANOVA to compare within the time interval	The statistical analysis was performed using three ways ANOVA to compare it with time and concentration.
7. The author did response to the comments but all the comments in the attached reviewed manuscript are missing.<br>Check and address all the comments	In this revision, all the relevant comments that were missed in the previous revision are addressed properly with comments section using track changes. Kindly check it.
8. The author responded partially to the comments, as there are several comments in the attached reviewed manuscript that are not yet addressed in the submitted revised manuscript	In this revision, all the relevant comments missing in previous revision are addressed properly with comments section.

Reviewer 3

Reviewer comments	Responses
1. The authors will need to present the results for the characterization of the nanoparticles before application. The scanning electron microscope for the nanoparticles were not presented.	SEM for the characterization of nanoparticles is represented in the figures.
2. Experiments are better presented as three biological replicates rather than three replicates.	Suggestions are incorporated.
3. The zone of inhibitions represented on plate was not interpreted statistically.	Incorporated.
4. The statistical application presented in the treatment figure 4 and figure 5 should be revisited.	Suggestions are corrected.

Reviewer 4

Reviewer comments	Responses
1. Abstract: The abstract is informative but could benefit from a more concise summary of the results and implications. Abbreviations (CuNPs, SiNPs, Cu-Si, etc.) should be expanded upon first use. Consider highlighting key findings and their significance in a more straightforward manner.	Suggestions are incorporated.
2. Introduction: While the introduction provides a solid background, it would be beneficial to include more recent	The introduction is up to date. Kindly have a look on all references. Just 2-3 references are below to year 2020. All the remaining are of year 2020, 2021, 2022, 2023, and 2024.

	references to strengthen the context of your research. The introduction is not up to date, and proper citations are missing. For example:<br>(a) Line 48: Pakistan ranks 8th in what? If you are referring to wheat, it is ranked 7th. Confirm this with the USDA and add the latest reference.<br>(b) Line 50: It appears that your references are not arranged correctly. Citations should start from 1, not 16. Follow the proper rules of citation.<br>(c) Additionally, clearly stating the research objectives at the end of this section would provide a clearer focus for readers.	a. It is confirmed that Pakistan stands at number 8th with respect to wheat production globally.<br>b. All the references are rearranged as per their appearance in the text.<br>c. Objectives are clearly stated at the end of introduction.
3.	Methodology: The methods used for synthesizing and testing nanoparticles are well described. However, additional details regarding the controls used in experiments would enhance reproducibility. Clarify how concentrations were determined and provide justification for the selected dosages.	The control is referred to as distilled water during the whole experiment. To get three concentrations (0.025, 0.05, and 0.075 %) of NPs, 0.025, 0.05, and 0.075 g of (Cu and Si) powder alone and in combination (Cu + Si) were added in bottle (250 mL) containing 100 mL distilled water separately. The nanoparticles cause toxicity to plants if applied at higher concentrations that's why small dosages are recommended.
4.	Results and Discussion: The results are presented clearly, but the discussion could be expanded to include comparisons with existing literature. Discussing potential mechanisms behind observed effects would provide deeper insights into your findings.	In the discussion, the current findings are supported by latest studies and compared with the existing literature. The potential mechanism behind the action of both copper and silicon nanoparticles is described very well.
5.	Figures: Ensure that all figures and tables are clearly labeled and referenced in the text. Some figures lack sufficient legends (e.g., Fig. 4, 5A, and 4B) explaining their significance. The photo quality is low; please upload high-resolution images (at least 300 dpi).	The Fig. 4, 5A and 5B are representing the data very well and their resolution is also at least 300 dpi.
6.	Tables: The table format is not suitable for publication. Please use the standard table format.	Table is removed from the manuscript emphasizing the formatting of the manuscript.
7.	Conclusion: The conclusion summarizes the findings effectively but should also	Future research directions are updated.

address future research directions or potential applications of your findings in agricultural practices.	
8. Statistical Analysis: Please provide more details regarding the statistical methods used for data analysis, including the software and specific tests applied.	Statistical analysis is performed by employing ANOVA (Analysis of Variance) with LSD test at $p \leq 0.05$ in software named as "Statistix 8.1". The graphical representation was performed on "Origin 2024b".
9. Side Effects: As parthenium is a causative agent of Parthenium dermatitis, what are the side effects of parthenium on wheat and ultimately on humans?	Parthenium dermatitis is one of the main constraints faced by human health as a prolonged exposure to *Parthenium hysterophorus* L. It can also cause respiratory risks like asthma and bronchitis.
10. The authors have incorporated the suggestions. However, in some comments, the authors did not follow the instructions. For example:<br>1. Pakistan ranks 7th in wheat production instead of 8th (https://fas.usda.gov/data/production/commodity/0410000).<br>2. The wheat contribution to agricultural value added and GDP is incorrect. The authors must update their manuscript according to the recent reference (Investigating the Impact of Agricultural Credit on Wheat Farming: Evidence from Pakistan, https://doi.org/10.3390/agriculture14122200).<br>I advise the authors to review the above information and verify it with the latest online or published data (if available). The purpose of the revision is to improve and provide globally accepted and authenticated data for publication.	All the comments raised during previous review were addressed in a scientific way.<br>1. This comment was also suggested to review and confirm the status of the Pakistan ranking for wheat production. It is confirmed by the data of FAOSTAT-2022 where it is clearly written that Pakistan ranked eighth in wheat production with respect to the globe. You can confirm this with this link of FAOSTAT (https://www.fao.org/faostat/en/#rankings/countries_by_commodity). Kindly check it. It will represent that Pakistan is ranked eighth in production of wheat.<br>2. The agricultural value added by the wheat and its role in GDP of Pakistan is confirmed with the official economic survey of Pakistan under agriculture section. Kindly confirm this using the given link to Economic survey of Pakistan (https://www.finance.gov.pk/survey/chapter_24/2_agriculture.pdf).
