# Supplementary material for: Multimodal impact of copper-silicon hybrid nanotools towards bacterial leaf streak, wheat biochemistry and productivity parameters
Source: PLoS One. 2025 Jul 15;20(7):e0327021. doi: 10.1371/journal.pone.0327021 (PMC12262875; doi:10.1371/journal.pone.0327021)
Supplement: Attachment — Submitted filename: Response to Reviewers (1).docx [file pone.0327021.s009.docx]

**PLOS ONE Reviewer comments**

**Reviewer 1**

| **Reviewer comments** | **Responses** |
| --- | --- |
| 1. The title needs to be clarified. | Incorporated |
| 2. I highly recommend reviewing the grammar of the paper. | Grammatical Errors are corrected. |
| 3. Introduction section; (a) Line 50, start the Intro with reference No. [16], How? (b) The references need to be rearranged. | All the references in the text are rearranged according to your prescription. |
| 4. Material and Methods section; (a) Line 106 “ml” change to mL and so on. (b) What specific synthesis techniques are most effective for combining silicon and copper nanoparticles? (c) This method of combination isn’t mentioned. (d) Why did you choose these three concentrations (0.025, 0.05, and 0.075 g). (e) Line 182 “ntreated” changed to untreated. | a. Changed to mL and so on. b. Simply the fine powder of both nanoparticles (Cu and Si) was mixed in a beaker and further used for their assessment under laboratory, greenhouse and field conditions. c. It is stated in line 117. d. Nanoparticles cause toxicity to plants at higher concentrations. That’s why lower concentrations were most effective for the best results. e. Incorporated. |
| 5. Results; (a) Please, add the UV-vis and SEM figures. | The SEM figures are attached. UV-vis pictures are not available. |

**Reviewer 2**

| **Reviewer comments** | **Responses** |
| --- | --- |
| 1. Throughout the manuscript check for English typos | Thoroughly proofread was done to remove any English typos errors. |
| 2. The title is too long, maximum should be 15 words. | Short title will not represent the focus of the research paper clearly, that’s why the title is quite longer. |
| 3. the introduction need more recent literatures, I suggested some. | As per your suggestions, literature provided is recent. Kindly have a look on it. Most of the references are above the year 2020 despite four references. |
| 4. the method should be referenced | Complete methodology is referenced. |
| 5. the figures and tables presenting the data very well, but the discussion should explain why the clear zone is increasing by the time | The suggestion is incorporated and explained why the clear zone is increased with increase in time. |
| 6. Statistical analysis should be done with two ways ANOVA to compare within the time interval | The statistical analysis was performed using three ways ANOVA to compare it with time and concentration. |
| 7. The author did response to the comments but all the comments in the attached reviewed manuscript are missing. Check and address all the comments | In this revision, all the relevant comments that were missed in the previous revision are addressed properly with comments section using track changes. Kindly check it. |

**Reviewer 3**

| **Reviewer comments** | **Responses** |
| --- | --- |
| 1. The authors will need to present the results for the characterization of the nanoparticles before application. The scanning electron microscope for the nanoparticles were not presented. | SEM for the characterization of nanoparticles is represented in the figures. |
| 2. Experiments are better presented as three biological replicates rather than three replicates. | Suggestions are incorporated. |
| 3. The zone of inhibitions represented on plate was not interpreted statistically. | Incorporated. |
| 4. The statistical application presented in the treatment figure 4 and figure 5 should be revisited. | Suggestions are corrected. |

**Reviewer 4**

| **Reviewer comments** | **Responses** |
| --- | --- |
| 1. Abstract: The abstract is informative but could benefit from a more concise summary of the results and implications. Abbreviations (CuNPs, SiNPs, Cu-Si, etc.) should be expanded upon first use. Consider highlighting key findings and their significance in a more straightforward manner. | Suggestions are incorporated. |
| 2. Introduction: While the introduction provides a solid background, it would be beneficial to include more recent references to strengthen the context of your research. The introduction is not up to date, and proper citations are missing. For example: (a) Line 48: Pakistan ranks 8th in what? If you are referring to wheat, it is ranked 7th. Confirm this with the USDA and add the latest reference. (b) Line 50: It appears that your references are not arranged correctly. Citations should start from 1, not 16. Follow the proper rules of citation. (c) Additionally, clearly stating the research objectives at the end of this section would provide a clearer focus for readers. | The introduction is up to date. Kindly have a look on all references. Just 2-3 references are below to year 2020. All the remaining are of year 2020, 2021, 2022, 2023, and 2024.   a. It is confirmed that Pakistan stands at number 8^th^ with respect to wheat production globally. b. All the references are rearranged as per their appearance in the text. c. Objectives are clearly stated at the end of introduction. |
| 3. Methodology: The methods used for synthesizing and testing nanoparticles are well described. However, additional details regarding the controls used in experiments would enhance reproducibility. Clarify how concentrations were determined and provide justification for the selected dosages. | The control is referred to as distilled water during the whole experiment. To get three concentrations (0.025, 0.05, and 0.075 %) of NPs, 0.025, 0.05, and 0.075 g of (Cu and Si) powder alone and in combination (Cu + Si) were added in bottle (250 mL) containing 100 mL distilled water separately. The nanoparticles cause toxicity to plants if applied at higher concentrations that’s why small dosages are recommended. |
| 4. Results and Discussion: The results are presented clearly, but the discussion could be expanded to include comparisons with existing literature. Discussing potential mechanisms behind observed effects would provide deeper insights into your findings. | In the discussion, the current findings are supported by latest studies and compared with the existing literature. The potential mechanism behind the action of both copper and silicon nanoparticles is described very well. |
| 5. Figures: Ensure that all figures and tables are clearly labeled and referenced in the text. Some figures lack sufficient legends (e.g., Fig. 4, 5A, and 4B) explaining their significance. The photo quality is low; please upload high-resolution images (at least 300 dpi). | The Fig. 4, 5A and 5B are representing the data very well and their resolution is also at least 300 dpi. |
| 6. Tables: The table format is not suitable for publication. Please use the standard table format. | Table is removed from the manuscript emphasizing the formatting of the manuscript. |
| 7. Conclusion: The conclusion summarizes the findings effectively but should also address future research directions or potential applications of your findings in agricultural practices. | Future research directions are updated. |
| 8. Statistical Analysis: Please provide more details regarding the statistical methods used for data analysis, including the software and specific tests applied. | Statistical analysis is performed by employing ANOVA (Analysis of Variance) with LSD test at *p* ≤ 0.05 in software named as “Statistix 8.1”. The graphical representation was performed on “Origin 2024b”. |
| 9. Side Effects: As parthenium is a causative agent of Parthenium dermatitis, what are the side effects of parthenium on wheat and ultimately on humans? | Parthenium dermatitis is one of the main constraints faced by human health as a prolonged exposure to *Parthenium hysterophorus* L. It can also cause respiratory risks like asthma and bronchitis. |
| 10. The authors have incorporated the suggestions. However, in some comments, the authors did not follow the instructions. For example: 1. Pakistan ranks 7th in wheat production instead of 8th (https://fas.usda.gov/data/production/commodity/0410000). 2. The wheat contribution to agricultural value added and GDP is incorrect. The authors must update their manuscript according to the recent reference (Investigating the Impact of Agricultural Credit on Wheat Farming: Evidence from Pakistan, https://doi.org/10.3390/agriculture14122200). I advise the authors to review the above information and verify it with the latest online or published data (if available). The purpose of the revision is to improve and provide globally accepted and authenticated data for publication. | All the comments raised during previous review were addressed in a scientific way.   1. This comment was also suggested to review and confirm the status of the Pakistan ranking for wheat production. It is confirmed by the data of FAOSTAT-2022 where it is clearly written that Pakistan ranked eighth in wheat production with respect to the globe. You can confirm this with this link of FAOSTAT   (<https://www.fao.org/faostat/en/#rankings/countries_by_commodity>). Kindly check it. It will represent that Pakistan is ranked eighth in production of wheat.   2. The agricultural value added by the wheat and its role in GDP of Pakistan is confirmed with the official economic survey of Pakistan under agriculture section. Kindly confirm this using the given link to Economic survey of Pakistan (<https://www.finance.gov.pk/survey/chapter_24/2_agriculture.pdf>). |
