# Supplementary material for: Multimodal impact of copper-silicon hybrid nanotools towards bacterial leaf streak, wheat biochemistry and productivity parameters
Source: PLoS One. 2025 Jul 15;20(7):e0327021. doi: 10.1371/journal.pone.0327021 (PMC12262875; doi:10.1371/journal.pone.0327021)
Supplement: Attachment — Submitted filename: Response to Reviewers.docx [file pone.0327021.s014.docx]

**PLOS ONE Reviewer comments**

**Additional Comments**

Modifications in the following references are done.

| **Reviewer comments** | **Responses** |
| --- | --- |
| The title is too long, max 15 words | Multimodal Impact Of Copper-Silicon Hybrid Nanotools On Bacterial Leaf Streak Management, Wheat Biochemistry And Productivity Parameters |
| Ref No. 5: Hussain D, Asrar M, Khalid B, Hafeez F, Saleem M, Akhter M, et al. Insect pests of economic importance attacking wheat crop (*Triticum aestivum* L.) in Punjab, Pakistan. Int J Trop Insect Sci. 2022 Feb;42(1): 9-20. doi: <https://doi.org/10.1007/s42690-021-00574-9> | Kaur N, Muslim Q, Dali VF, Anshu A, Siddharth T, Zienab FA. CRISPR/Cas9: A sustainable technology to enhance climate resilience in major staple crops. Front Genome Ed. 2025 March 18;  7: 1533197. <https://doi.org/10.3389/fgeed.2025.1533197> |
| Ref No. 10: Ledman KE, Curland RD, Ishimaru CA, Dill-Macky R. *Xanthomonas translucens* pv. *undulosa* identified on common weedy grasses in naturally infected wheat fields in Minnesota. Phytopathol. 2021 Jul 30;111(7): 1114-1121. doi: <https://doi.org/10.1094/PHYTO-08-20-0337-R> | Hussain S, Aqleem A, Maratab A, Israt J, Muhammad J, Muhammad A.A, et al. Diversity of Alternaria-derived toxins and their toxicodynamic and toxicokinetic characteristics in the food chain. Food Front. 2025; 6(1): 185-217. <https://doi.org/10.1002/fft2.507> |
| Ref No. 17: Shafqat U, Maqsood A, Ishfaq A, Mustafa S, Rasheed Y, Mahmood F, et al. Green nanotechnology for plant bacterial diseases management in cereal crops: a review on metal-based nanoparticles. Notulae Bot Horti Agrobotan Cluj-Napoca. 2023 Sep 27;51(3): 13333. doi: <https://doi.org/10.15835/nbha51313333> | Asif A, Maratab A, Muslim Q, Rajmohan K, Zora S, Ravjit K, et al. Enhancing crop resilience by harnessing the synergistic effects of biostimulants against abiotic stress. Front Plant Sci. 2023 Dec 18; 14: 1276117. <https://doi.org/10.3389/fpls.2023.1276117> |
| Ref No. 22: Kashyap P, Shirkot P, Das R, Pandey H, Singh D. Biosynthesis and characterization of copper nanoparticles from *Stenotrophomonas maltophilia* and its effect on plant pathogens and pesticide degradation. J Agri Food Res. 2023 Sep 1;13: 100654. doi: <https://doi.org/10.1016/j.jafr.2023.100654> | Francis DV, Abdalla AK, Mahakham W, Sarmah AK, Ahmed ZF. Interaction of plants and metal nanoparticles: Exploring its molecular mechanisms for sustainable agriculture and crop improvement. Environ Int. 2024 Aug; 190: 108859. doi: <https://doi.org/10.1016/j.envint.2024.108859> |
| Ref No. 27: Ali S, Ulhassan Z, Shahbaz H, Kaleem Z, Yousaf MA, Ali S, et al. Magnesium oxide nanoparticles as novel sustainable approach in enhancing crop tolerance to abiotic and biotic stresses. Environ Sci: Nano. 2024 Jul 5;11(8): 3250-3267. doi: <https://doi.org/10.1039/D4EN00417E> | Francis DV, Anam A, Zienab FRA. Nanoparticle-enhanced plant defense mechanisms harnessed by nanotechnology for sustainable crop protection. In: Masudulla K, Jen-Tsung C, editor. Nanoparticles in plant biotic stress management. Springer Nature; 2024 June 08. pp. 451-491. doi: <https://doi.org/10.1007/978-981-97-0851-2_19> |
| Ref No. 30: Kralova K, Jampilek J. Applications of nanomaterials in plant disease management and protection. In: Ingle AP, editor. Nanotechnology in agriculture and agroecosystems. Micro Nano Tech. 2023 Jan 1: 239-296. doi: <https://doi.org/10.1016/B978-0-323-99446-0.00013-1> | Francis DV, Abdul S, Abdel-Hamid IM, Abdelmoneim KA, Zienab FRA. Optimizing germination conditions of Ghaf seed using ZnO nanoparticle priming through Taguchi method analysis. Sci Rep. 2024 July 10;14: 15946. doi: <https://doi.org/10.1038/s41598-024-67025-6> |
