# Supplementary material for: Multimodal impact of copper-silicon hybrid nanotools towards bacterial leaf streak, wheat biochemistry and productivity parameters
Source: PLoS One. 2025 Jul 15;20(7):e0327021. doi: 10.1371/journal.pone.0327021 (PMC12262875; doi:10.1371/journal.pone.0327021)
Supplement: Attachment — Submitted filename: PLOS ONE Reviewer comments (16-4-2025).docx [file pone.0327021.s013.docx]

**PLOS ONE Reviewer comments**

| **Reviewer comments** | **Responses** |
| --- | --- |
| Shafqat U, Maqsood A, Ishfaq A, Mustafa S, Rasheed Y, Mahmood F, et al. Green nanotechnology for plant bacterial diseases management in cereal crops: a review on metal-based nanoparticles. Notulae Bot Horti Agrobotan Cluj-Napoca. 2023 Sep 27;51(3): 13333. doi: https://doi.org/10.15835/nbha51313333 | The highlighted reference is Replaced with:  Francis DV, Abdalla AK, Mahakham W, Sarmah AK, Ahmed ZF. Interaction of plants and metal nanoparticles: Exploring its molecular mechanisms for sustainable agriculture and crop improvement. Environ Int. 2024 Aug; 108859. doi: <https://doi.org/10.1016/j.envint.2024.108859> |
| Ameta SK, Ameta SC. Eco-friendly approaches of using weeds for sustainable plant growth and production. In: Husen A, editor. Plant performance under environmental stress. Springer, Singapore; 2021. pp. 559-592. doi: <https://doi.org/10.1007/978-3-030-78521-5> | The highlighted reference is Replaced with:  Francis DV, Abdalla AK, Mahakham W, Sarmah AK, Ahmed ZF. Interaction of plants and metal nanoparticles: Exploring its molecular mechanisms for sustainable agriculture and crop improvement. Environ Int. 2024 Aug; 108859. doi: <https://doi.org/10.1016/j.envint.2024.108859> |
| Javaid A, Munir R, Khan IH, Shoaib A. Control of the chickpea blight, Ascochyta rabiei, with the weed plant, Withania somnifera. Egypt J Biol Pest Cont. 2020 Dec;30: 114. doi: https://doi.org/10.1186/s41938-020-00315-z | The highlighted reference is Replaced with  Asif A, Ali M, Qadir M, Karthikeyan R, Singh Z, Khangura R, Di Gioia F, Ahmed ZF. Enhancing crop resilience by harnessing the synergistic effects of biostimulants against abiotic stress. Front Plant Sci. 2023 Dec; 14: 1276117. doi: <https://doi.org/10.3389/fpls.2023.1276117> |
